# Supplementary material for: Resolving and Rescuing Developmental Miswiring in a Mouse Model of Cognitive Impairment
Source: Neuron. 2020 Jan 8;105(1):60–74.e7. doi: 10.1016/j.neuron.2019.09.042 (PMC6953432; doi:10.1016/j.neuron.2019.09.042)
Supplement: Document S1. Figures S1–S9 and Table S1 [file mmc1.pdf]

Neuron, Volume *105*

Supplemental Information

Resolving and Rescuing Developmental Miswiring in a Mouse Model of Cognitive Impairment

Mattia Chini, Jastyn A. Pöpplau, Christoph Lindemann, Laura Carol-Perdiguer, Marilena Hnida, Victoria Oberländer, Xiaxia Xu, Joachim Ahlbeck, Sebastian H. Bitzenhofer, Christoph Mulert, and Ileana L. Hanganu-Opatz

Resolving and rescuing developmental miswiring in a mouse model of cognitive impairment

Mattia Chini[1], Jastyn A. Pöpplau[1], Christoph Lindemann[1], Laura Carol-Perdiguer[1], Marilena Hnida[1], Victoria Oberländer[1,†], Xiaxia Xu[1], Joachim Ahlbeck[1,††], Sebastian H. Bitzenhofer[1,†††], Christoph Mulert[2,§] & Ileana L. Hanganu-Opatz[1,3*]

Inventory of Supplemental Information	page
Figure S1, related to Fig. 1	2
Figure S2, related to Fig. 1	4
Figure S3, related to Fig. 2-5	6
Figure S4, related to Fig. 2-3	8
Figure S5, related to Fig. 2	10
Figure S6, related to Fig. 3	11
Figure S7, related to Fig. 4	12
Figure S8, related to Fig. 5-6	14
Figure S9, related to Fig. 8	16
Data Files S1	17
Table S1	18

SUPPLEMENTARY INFORMATION

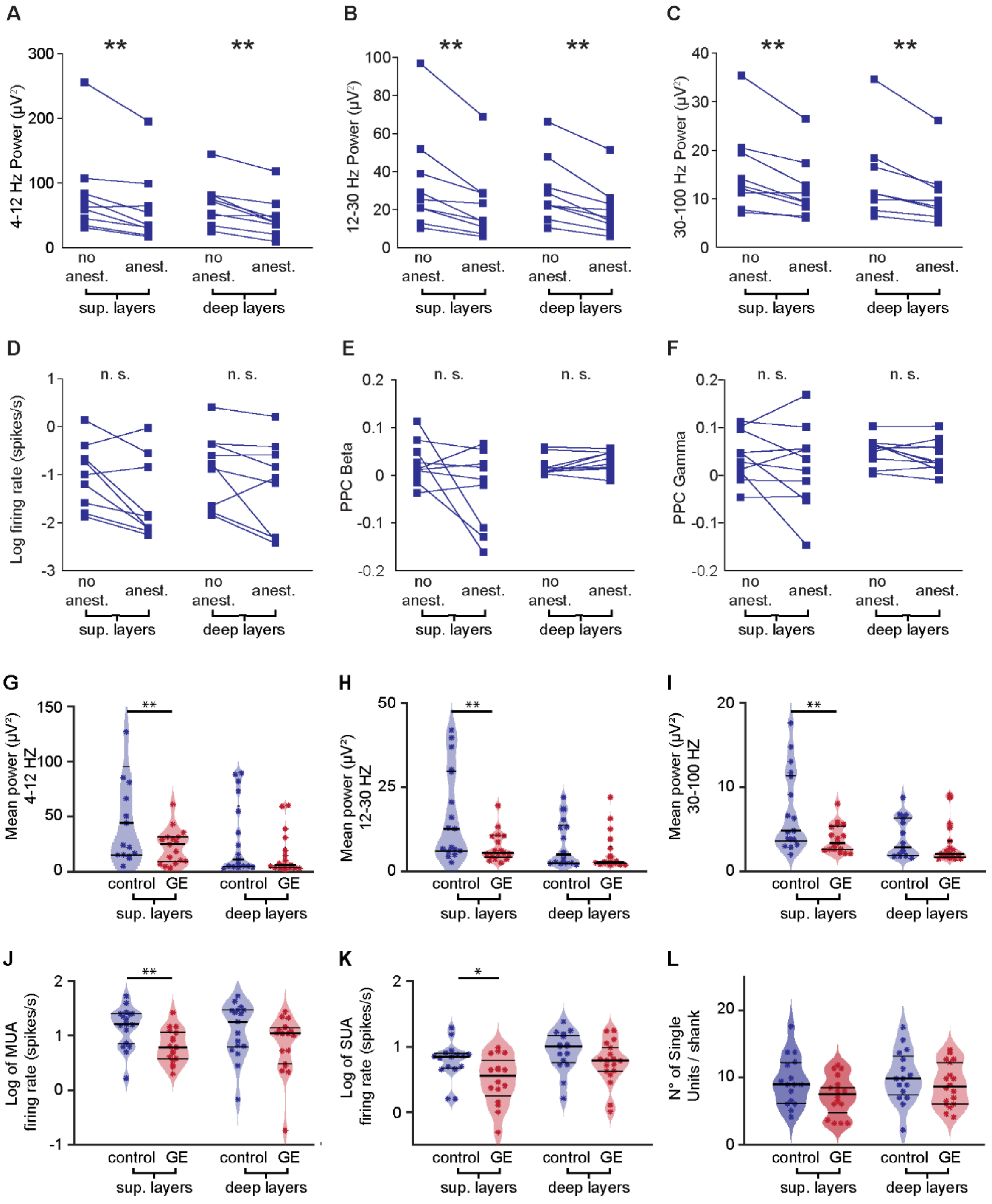

Figure S1. Related to Figure 1. Effect of urethane anesthesia on network oscillations and firing of neonatal control and GE mice. (A) Scatter plot displaying the oscillatory power in theta frequency band for superficial and deep layers of the prelimbic cortex before (left, n=9 mice) and after urethane anesthesia (right, n=9 mice). **(B-F)** Same as (A) for power in beta (B) and gamma (C) frequency bands, MUA firing rate (D), as well as pairwise phase consistency for beta (E) and gamma (F) oscillations. **(G)** Violin plot displaying the power for theta frequency band in superficial and deep layers of the prelimbic cortex of non-anesthetized control (blue, n=16) and GE (red, n=18) mice. **(H-L)** Same as (G) for power in beta (H) and gamma (I)

frequency bands, MUA (J) and SUA (K) firing rate, and number of single units per shank (L). In scatter plots (A-F), single data points are presented as squares. In violin plots (G-L), data is presented as median with 25th and 75th percentile and single data points are shown as asterisks. *P<0.05, **P<0.01 and ***P<0.001, Wilcoxon signed-rank test (A-F), ANCOVA with age as covariate (G-L), and yuen's bootstrap test (J-L) with 20% level of trimming for the mean.

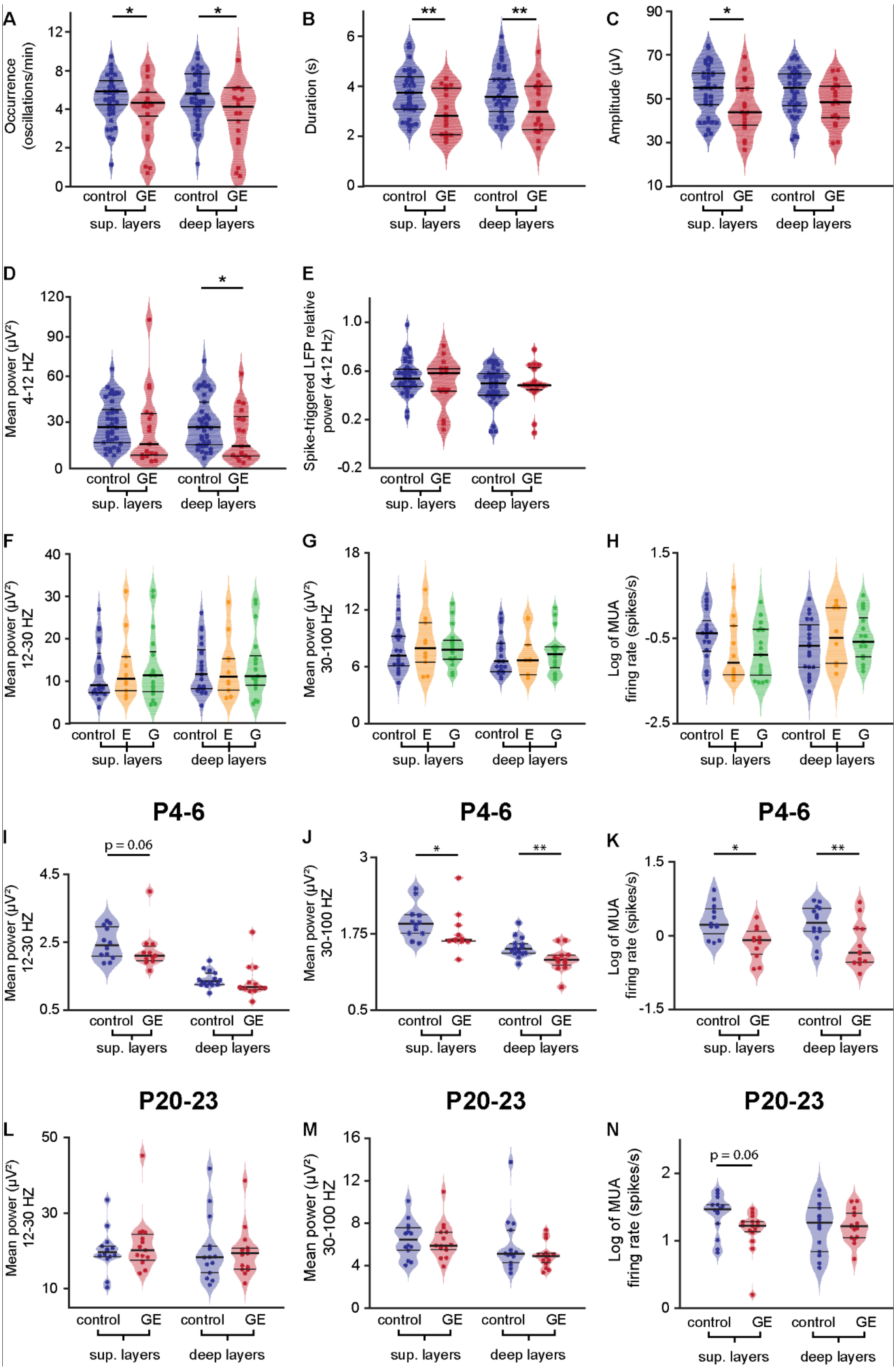
A
Occurrence (oscillations/min)
B
Duration (s)
C
Amplitude (µV)
D
Mean power (µV²) 4-12 HZ
E
Spike-triggered LFP relative power (4-12 Hz)
F
Mean power (µV²) 12-30 HZ
G
Mean power (µV²) 30-100 HZ
H
Log of MUA firing rate (spikes/s)
P4-6
I
p = 0.06
Mean power (µV²) 12-30 HZ
J
Mean power (µV²) 30-100 HZ
K
Log of MUA firing rate (spikes/s)
P20-23
L
Mean power (µV²) 12-30 HZ
M
Mean power (µV²) 30-100 HZ
N
p = 0.06
Log of MUA firing rate (spikes/s)
control
GE
E
G
sup. layers
deep layers

Figure S2. Related to Figure 1. Properties of discontinuous oscillatory activity and neuronal firing over the layers of PL from neonatal one-hit and dual-hit GE mice as well as P4-6 and P20-23 control and dual-hit GE mice. (A) Violin plot displaying the occurrence of oscillations in prelimbic superficial and deep layers of control (blue, n=38) and GE (red, n=18) mice. **(B-E)** Same as (A) for oscillation duration (B), amplitude (C), power in theta frequency band (D), the relative power of spike-triggered LFP in theta band (E). **(F)** Violin plot displaying the mean power (12-30 Hz) of oscillations in prelimbic superficial and deep layers of control (blue, n=21), E (yellow, n=10) and G (green, n=17) neonatal mice. (**G,H**) Same as (F) for mean power (30-100 Hz) (G) and MUA firing rate (H). (**I**) Violin plot displaying the mean power (12-30 Hz) in prelimbic superficial and deep layers of control (blue, n=14) and GE (red, n=13) P4-6 mice. (**J,K**) Same as (I) for mean power (30-100 Hz) (J) and MUA firing rate (K). (**L**) Violin plot displaying the mean power (12-30 Hz) in prelimbic superficial and deep layers of control (blue, n=14) and GE (red, n=15) P20-23 mice. (**M,N**) Same as (L) for mean power (30-100 Hz) (M) and MUA firing rate (N). Data is presented as median with 25th and 75th percentile and single data points are shown as asterisks. The shaded area represents the probability distribution of the variable. *P<0.05, **P<0.01, ANCOVA with age as covariate (A-D, F-K), and yuen's bootstrap test (E, I-N) with 20% level of trimming for the mean.

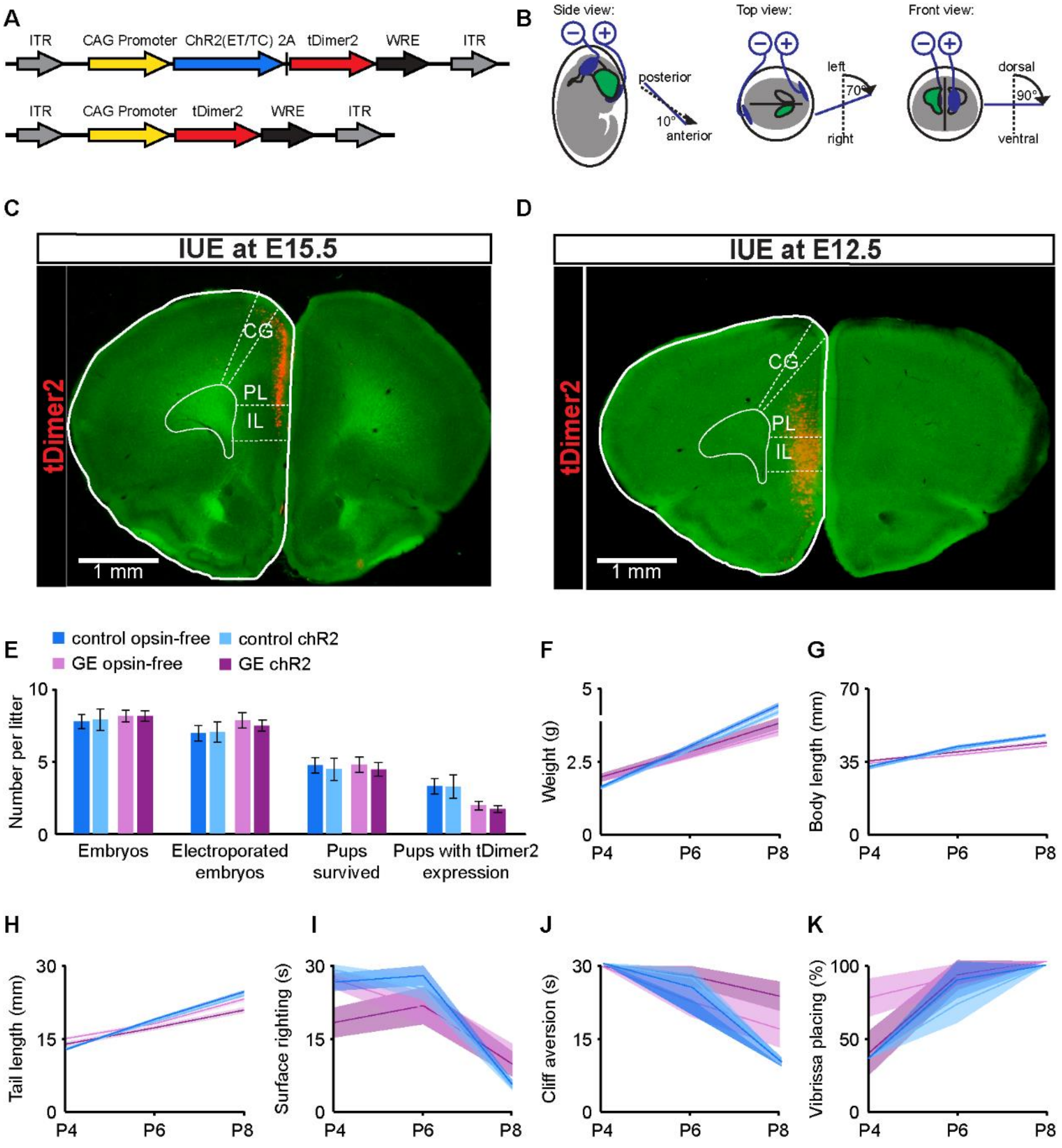

Figure S3. Related to Figures 2-5. Cell- and layer-specific transfection of neonatal prelimbic cortex by site-directed in utero electroporation. (a) Structure of the ChR2(ET/TC)-containing and opsin-free plasmids. **(B)** Schematic drawing illustrating the orientation of electrode paddles for specific targeting of $PYRs_{SUP}$ and $PYRs_{DEEP}$. **(C)** TDimer2-expressing cells (red) in a 50 µm-thick coronal section of a P10 mouse at the level of PFC after IUE at E15.5. Note that the transfection is restricted to neurons located in superficial layers. **(D)** Same as (C) after IUE at E12.5. Note that the transfection is mainly restricted to neurons located in deep layers. **(E)** Bar diagram displaying the mean number of embryos, electroporated embryos, surviving pups, and positively transfected pups when ChR2(ET/TC)-containing (control, dark blue, n=13 litters; GE, dark purple, n=10 litters) and opsin-free constructs (control, light blue, n=5 litters; GE, light purple, n=8 litters) were transfected by IUE at E15.5. **(F-H)** Line plots displaying the developmental profile of somatic growth: weight (F), body length (G), tail length (H) of P2-8 pups expressing ChR2(ET/TC) (control, dark blue, n=31; GE, dark purple, n=26) or opsin-free constructs (control, light blue, n=11; GE, light purple, n=7) in $PYRs_{SUP}$. **(I-K)** Line plots displaying the development profile of reflexes: surface righting (I), cliff aversion (J), and vibrissa placing (K) of P2-8 pups expressing ChR2(ET/TC)

(control, dark blue, n=31; GE, dark purple, n=26) or opsin-free constructs (control, light blue, n=11; GE, light purple, n=7) in $PYRs_{SUP}$. Data are displayed as mean ± SEM. *$p < 0.05$, one-way repeated-measures analysis of variance (ANOVA) with Bonferroni-corrected post hoc analysis.

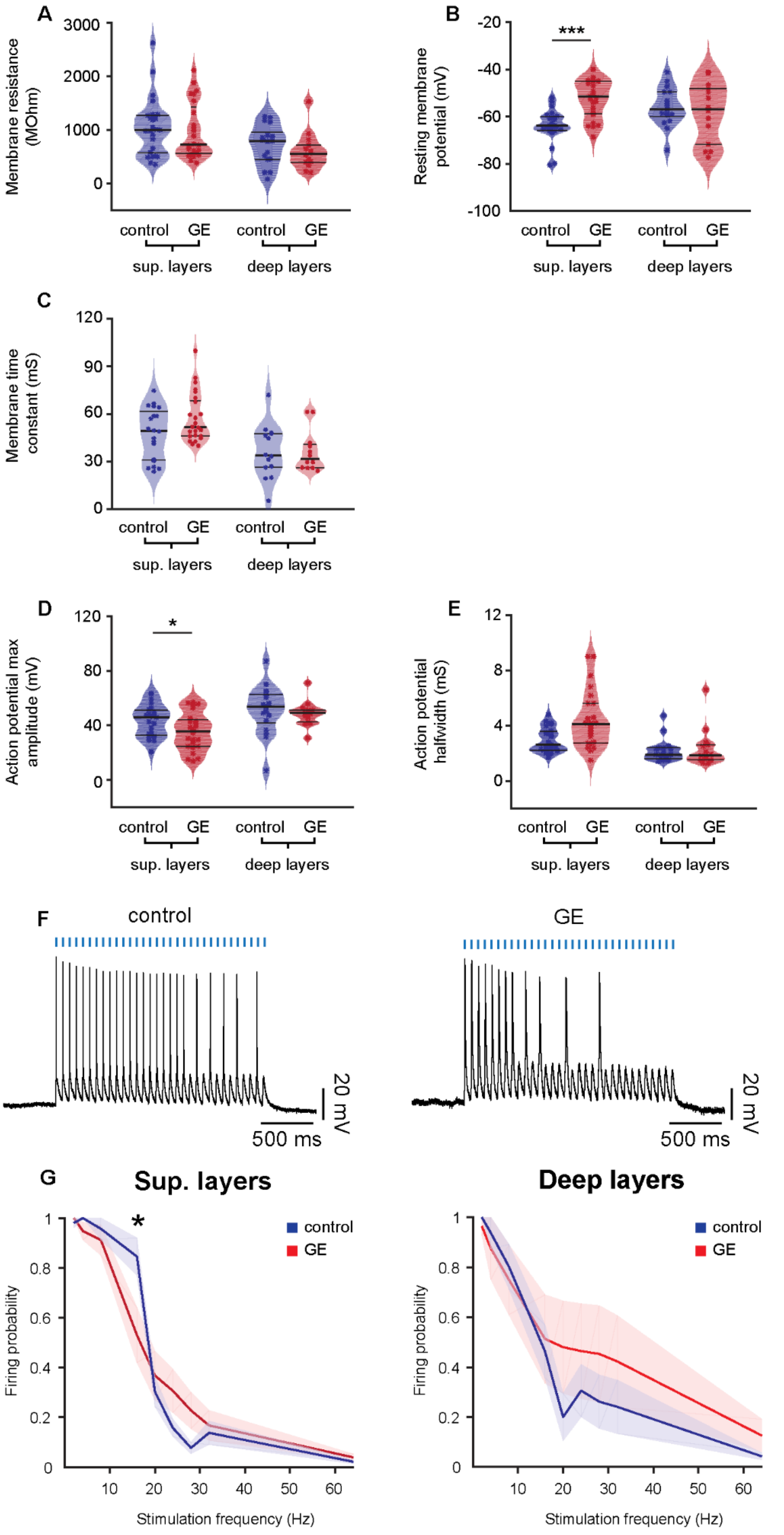

Figure S4. Related to Figures 2-3. Optogenetic activation of PYRs$_{SUP}$ and PYRs$_{DEEP}$ in control and dual-hit GE mice in vitro. (A) Violin plot displaying the membrane resistance for

$PYRs_{SUP}$ and $PYRs_{DEEP}$ of control (blue, n=25 and n=24, respectively) and GE mice (red, n=17 and n=14, respectively). **(B-E)** Same as (A) for resting membrane potential (B), membrane time constant (C), maximum light-triggered action potential amplitude (D), and light-triggered action potential half-width (E). **(F)** Representative voltage responses of a transfected $PYRs_{SUP}$ from a P9 control (left) and a P9 GE mouse (right) to repetitive trains of 3 ms-long light pulses at 16 Hz. **(G)** Graph displaying the mean firing probability of superficial (left) and deep layers (right) transfected control and GE neurons in response to repetitive light stimuli of different frequencies. In (G) data is presented as mean ± sem. In (A-E) data is presented as median with 25th and 75th percentile and single data points are shown as asterisks. The shaded area represents the probability distribution of the variable. *P<0.05, **P<0.01 and ***P<0.001, one-way repeated-measures analysis of variance (ANOVA) with Bonferroni-corrected post hoc analysis (F), and bootstrap yuen test with 20% level of trimming for the mean and 5000 bootstrap samples.

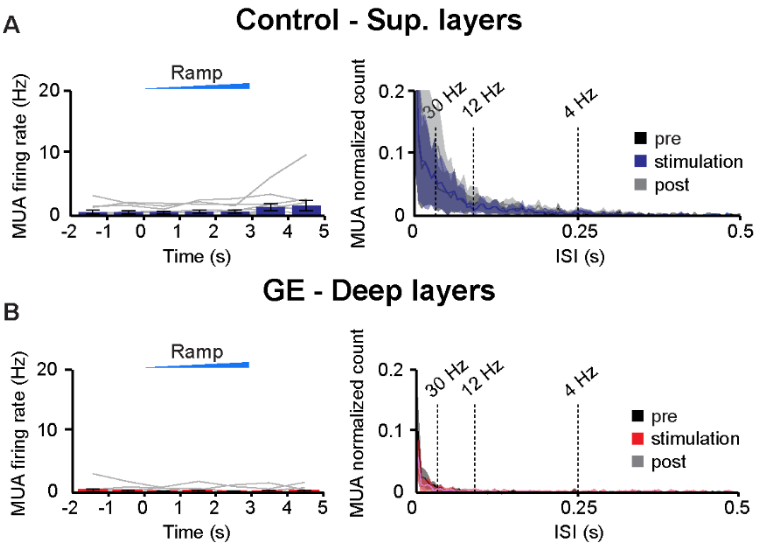

Figure S5. Related to Figure 2. In vivo spiking of PYRs$_{SUP}$ transfected by IUE with opsin-free constructs. (A) Left, bar diagram displaying the mean MUA firing rate in transfected PYRs$_{SUP}$ of control mice in response to ramp illumination. Right, occurrence rate of interspike intervals averaged for 3 s before light stimulation (pre, black), 3 s during ramp stimulation (stimulation, blue) and 3 s after light stimulation (post, grey, n=11 recording sites from 11 pups). **(B)** Same as (A) for PYRs$_{SUP}$ of GE mice (n=10 recording sites from 8 pups). Data is presented as mean ± s.e.m. P>0.05, one-way repeated-measures analysis of variance (ANOVA) with Bonferroni-corrected post hoc analysis.

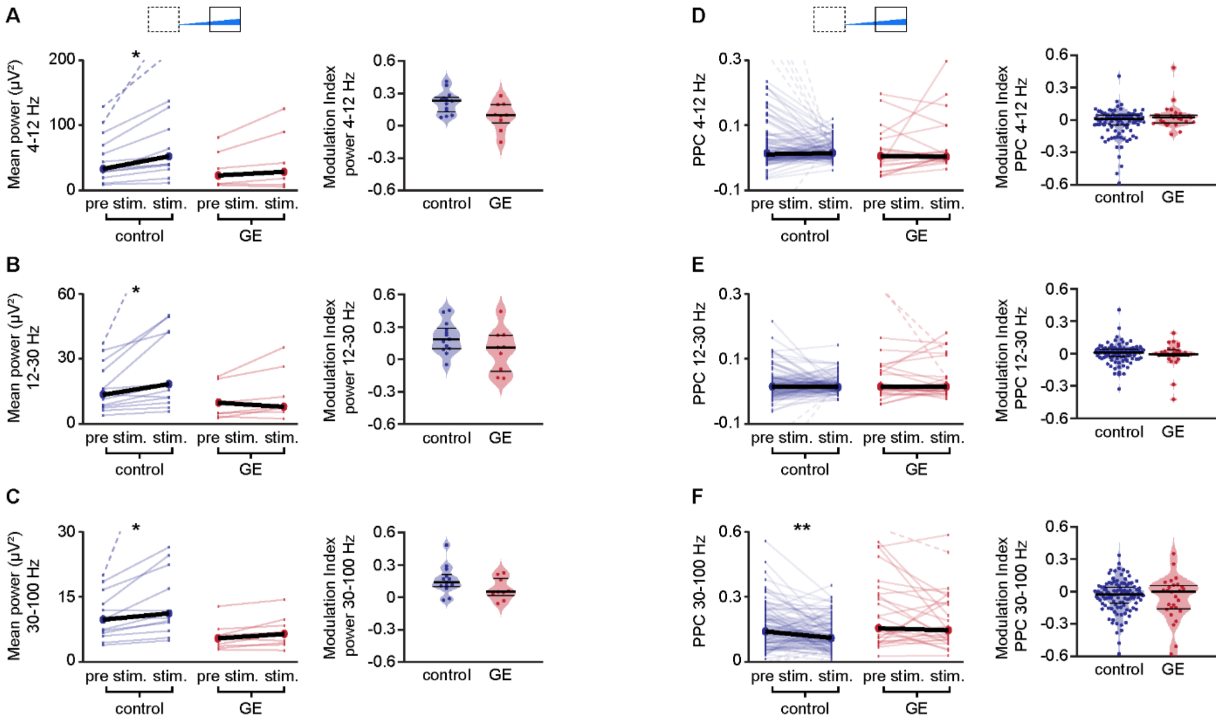

Figure S6. Related to Figure 3. Network activity after optogenetic activation of PYRs$_{DEEP}$ in control and dual-hit GE mice in vivo. (A) Left, scatter plot displaying the LFP power in the theta (4–12 Hz) frequency band for control (blue, n=13) and GE (red, n=9) mice before (pre stim., 1.5 s) and during the second half (stim., 1.5 s) of ramp stimulation. Right, violin plot displaying the stimulation modulation index of LFP power in the theta frequency band for control and GE mice. **(B,C)** Same as (A) for beta (12-30 Hz) and gamma (30-100) frequency bands. **(D-F)** Same as (A-C) for PPC of PYRs$_{DEEP}$ in control (n=116 recording sites from 13 pups) and GE (n=27 recording sites from 6 pups) mice. In scatter plots (A-F) data is presented as median, and individual values are displayed as thin dots and lines. In violin plots (A-F) data is presented as median with 25th and 75th percentile and single data points are shown as asterisks. The shaded area represents the probability distribution of the variable. *P<0.05, **P<0.01 and ***P<0.001, yuen's bootstrap test (A-F) with 20% level of trimming for the mean and linear mixed-effect model with animal as a random effect (D-F).

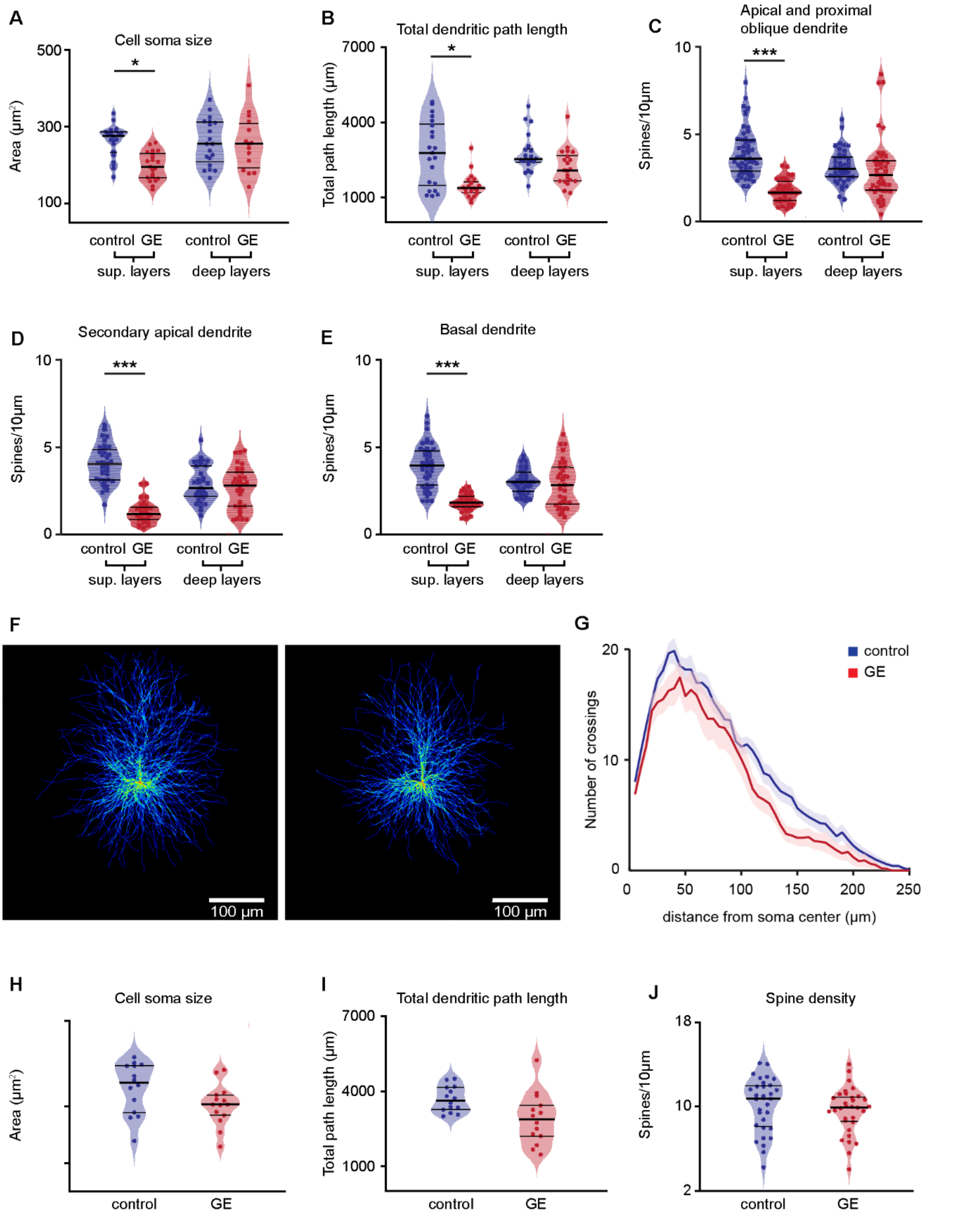

Figure S7. Related to Figure 4. Reduced soma size, dendritic path length and spine density in PYRs$_{SUP}$ of neonatal but not pre-juvenile dual-hit GE mice. (A) Left, violin plot displaying the average soma size from PYRs$_{SUP}$ of control (blue, n=21 neurons) and GE (red, n=21 neurons) mice. Right, same as left for PYRs$_{DEEP}$ from control (blue, n=21 neurons) and GE (red, n=15 neurons) mice. **(B)** Same as (A) for total dendritic path length. **(C)** Same as (A) for spine density on apical and proximal oblique dendrites from PYRs$_{SUP}$ of control (blue, n=53 dendrites from 13 neurons) and GE (red, n=35 dendrites from 10 neurons) mice. Right, same as left for PYRs$_{DEEP}$ from control (blue, n=43 dendrites from 12 neurons) and GE (red, n=44

dendrites from 12 neurons) mice. **(D,E)** same as (C) for secondary apical (B) and basal dendrites (C). **(F)** Heatmaps displaying an overlay of all traced dendrites of transfected $PYRs_{SUP}$ in pre-juvenile control (left) and GE (right) mice. **(G)** Graph displaying the average number of dendritic intersections within a 250 μm radius from the soma center of $PYRs_{SUP}$ in pre-juvenile control (blue, n=14 neurons from 2 pups) and GE (red, n=14 neurons from 2 pups) mice. **(H)** Violin plot displaying the average soma size from $PYRs_{SUP}$ of pre-juvenile control (blue, n=14 neurons) and GE (red, n=21 neurons) mice. (**I**) Same as (H) for total dendritic path length. (**J**) Violin plot displaying the average spine density on dendrites from $PYRs_{SUP}$ of pre-juvenile control (blue, n=24 dendrites from 8 neurons) and GE (red, n=24 dendrites from 8 neurons) mice. In (G) data is presented as mean ± s.e.m. In (A-E, H-J) data is presented as median with 25th and 75th percentile and single data points are shown as asterisks. The shaded area represents the probability distribution of the variable. ***$P<0.001$, linear mixed-effect model with animal as a random effect.

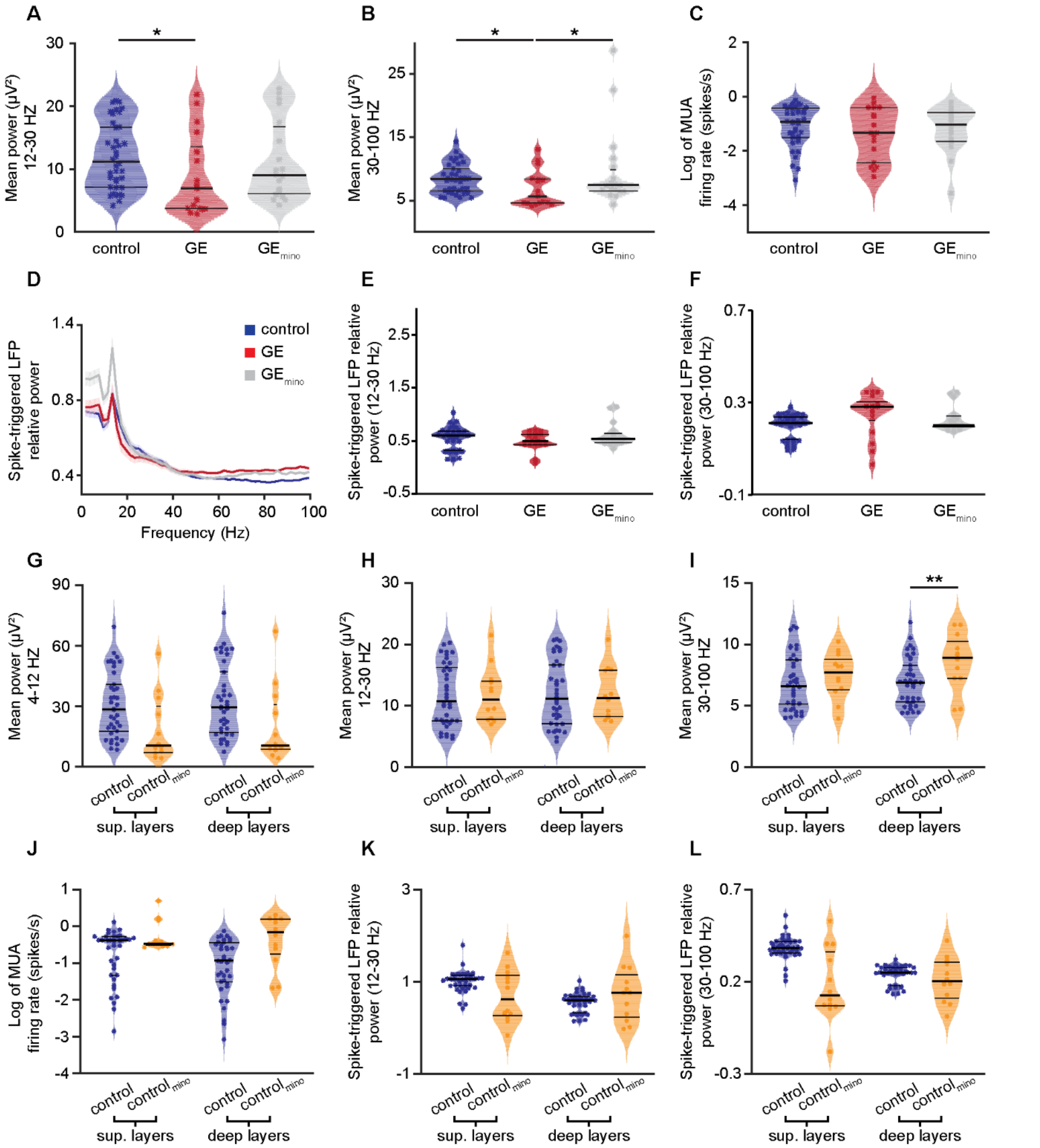

Figure S8. Related to Figure 5-6. Minocycline effect on neonatal control and dual-hit GE mice. (A) Violin plot displaying the power in beta frequency band of oscillations in deep layers of the PL of control (blue, n=38), GE (red, n=18), and GE_{mino} mice (gray, n=18). **(B-C)** Same as (A) for power in gamma (B) frequency bands and MUA firing rate (C). **(D)** Plots of frequency-dependent relative power of spike-triggered LFP in deep layers (top) of control (blue), GE (red), and GE_{mino} mice (gray). **(E)** Violin plot displaying the relative power of spike-triggered LFP in beta band for deep layers of control (blue, n=38), GE (red, n=18), and GE_{mino} mice (gray, n=18). **(F)** Same as (E) for the LFP in gamma band. **(G)** Violin plot displaying the power in theta frequency band of oscillations in superficial and deep layers of the prelimbic cortex of control (blue, n=38) and $control_{mino}$ (yellow, n=12) mice. **(H-L)** Same as (G) for the power in beta (H) and gamma (I) frequency band, MUA firing rate (J), relative power of spike-triggered LFP in beta (K) and gamma (L) band. In (D) data is presented as mean ± sem. In (A-C,E-L) data is presented as median with 25th and 75th percentile and single data points are shown as

asterisks. The shaded area represents the probability distribution of the variable. *P<0.05, **P<0.01, ANCOVA with age as covariate and 20% level of trimming for the mean (A-C), robust ANOVA with 20% level of trimming for the mean (E-F) with Bonferroni-Holm post-hoc analysis, and yuen’s bootstrap test (G-L) with 20% level of trimming for the mean.

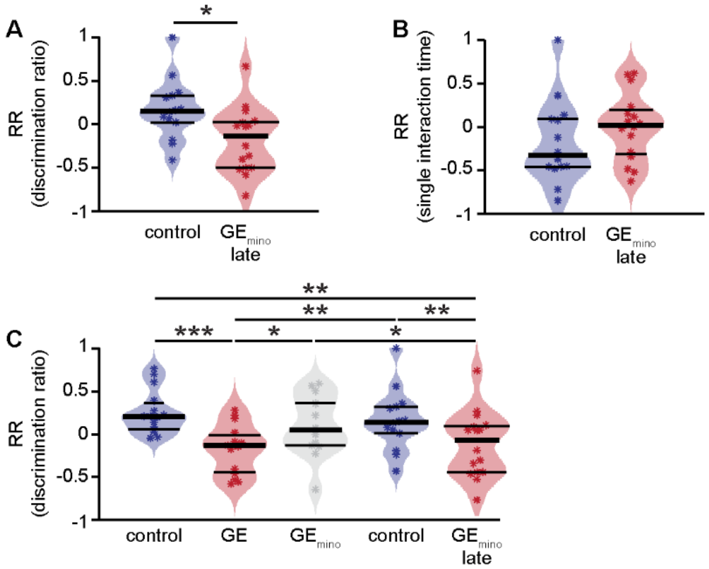

Figure S9. Related to Figure 8. Late minocycline treatment does not rescue behavioral deficits in dual-hit GE mice. (A) Violin plot displaying RR discrimination ratio of control (blue, n=14) and GE_{mino}late (red, n=15). **(B)** Same as (A) for single interaction time. **(C)** Same as (A) for control (blue, n=16), GE (red, n=15), GE_{mino} (gray, n=16), control (blue, n=14) and GE_{mino}late (red, n=15). Data is presented as median with 25th and 75th percentile and single data points are shown as asterisks. The shaded area represents the probability distribution of the variable. *P<0.05, robust, bootstrapped ANOVA with 20% level of trimming for the mean.

Data files S1. Related to Figures 1-8. Detailed statistical results.

	Superficial layers			*Deep layers*		
	**Control**	**GE**	**GE+mino**	**Control**	**GE**	**GE+mino**
Occurrence (oscillations/min)	6.6 ± 0.02	5.4 ± 0.2	6.7 ± 0.1	6.5 ± 0.05	5.2 ± 0.3 *	7 ± 0.1
Duration (s)	3.6 ± 0.1	3 ± 0.3 *	3.2 ± 0.03	3.7 ± 0.01	3 ± 0.26 *	3.2 ± 0.02
Amplitude (μV)	54.5 ± 1.1	45.7 ± 2.3 *	51.2 ± 0.4	54.3 ± 0.9	48.3 ± 1.6 *	49.8 ± 0.6
Theta power (μV^2)	28.9 ± 2.2	19.5 ± 4.4 **	23.6 ± 3.8	29.7 ± 2.5	19.3 ± 3.6 **	25.0 ± 4.8
Beta power (μV^2)	13.7 ± 0.7	7.0 ± 1.3 */°	10.2 ± 1.3	15.2 ± 0.7	6.9 ± 1.2 **	10.1 ± 1.4
Gamma power (μV^2)	6.6 ± 0.3	4.8 ± 0.4 **/°°	6.6 ± 0.7	6.7 ± 0.3	5.2 ± 0.5 **/°°	6.2 ± 0.5
Log firing rate (spikes/s)	-0.61 ± 0.04	-2-1 ± 0.1 ***/°°°	-0.9 ± 0.1	-0.95 ± 0.05	-1.3 ± 0.2	-0.96 ± 0.2
Spike triggered LFP relative power (4-12 Hz)	0.81 ± 0.02	0.89 ± 0.05 °°	1.3 ± 0.07 ^^	0.73 ± 0.03	0.67 ± 0.05 °	0.89 ± 0.05 ^
Spike triggered LFP relative power (12-30 Hz)	1.1 ± 0.03	0.64 ± 0.06 ***/°°	1.4 ± 0.17 ^	0.60 ± 0.04	0.50 ± 0.03	0.53 ± 0.03
Spike triggered LFP relative power (30-100 Hz)	0.38 ± 0.07	0.26 ± 0.03 ^/ */°	0.39 ± 0.02	0.25 ± 0.08	0.32 ± 0.02	0.24 ± 0.08

Table S1. Related to Figures 1, 6. Properties of oscillations in superficial and deep layers of PL in control, GE and GE_{mino} mice, as well as of their MUA firing and its timing to network oscillations. Asterisks (*) indicate significance level between control and GE mice. Circles (°) indicate significance level between GE and GE_{mino} mice. Carets (^) indicate significance level between control and GE_{mino} mice. ^/ */° p<0.05, ^^/ **/°° p<0.005, ^^^/ ***/°°° p<0.001.
