# Supplementary material for: Resolving and Rescuing Developmental Miswiring in a Mouse Model of Cognitive Impairment
Source: Neuron. 2020 Jan 8;105(1):60–74.e7. doi: 10.1016/j.neuron.2019.09.042 (PMC6953432; doi:10.1016/j.neuron.2019.09.042)
Supplement: Document S2. Article plus Supplemental Information [file mmc3.pdf]

Article

Neuron

Resolving and Rescuing Developmental Miswiring in a Mouse Model of Cognitive Impairment

Graphical Abstract

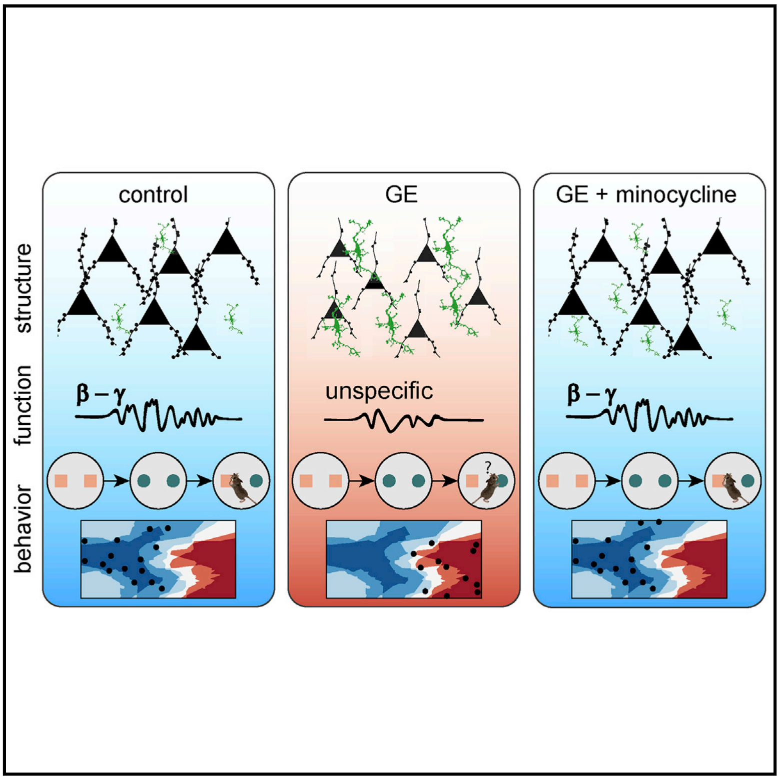

Authors

Mattia Chini, Jastyn A. Pöpplau, Christoph Lindemann, ..., Sebastian H. Bitzenhofer, Christoph Mulert, Ileana L. Hanganu-Opatz

Correspondence

hangop@zmnh.uni-hamburg.de

In Brief

In a mouse model of mental disorders, Chini et al. dissect an early-emerging prefrontal network dysfunction that subsequently gives rise to cognitive deficits. They show that this deficiency can be rescued by minocycline administration, thus identifying a potential biomarker amenable for future therapies.

Highlights

- Mice mimicking the etiology of mental illness have dysregulated prefrontal network
- Weaker beta activation of prefrontal circuits results from superficial layers deficits
- Rescue of microglial function restores developing prefrontal function and behavior
- Early prefrontal dysfunction relates to later-emerging cognitive performance

Chini et al., 2020, Neuron *105*, 60–74
January 8, 2020 © 2019 The Authors. Published by Elsevier Inc.
https://doi.org/10.1016/j.neuron.2019.09.042

CellPress

Neuron
Article

Resolving and Rescuing Developmental Miswiring in a Mouse Model of Cognitive Impairment

Mattia Chini,[1] Jastyn A. Pöpplau,[1] Christoph Lindemann,[1] Laura Carol-Perdiguer,[1] Marilena Hnida,[1] Victoria Oberländer,[1,3] Xiaxia Xu,[1] Joachim Ahlbeck,[1,4] Sebastian H. Bitzenhofer,[1,5] Christoph Mulert,[2,6] and Ileana L. Hanganu-Opatz[1,7,*]

[1]Developmental Neurophysiology, Institute of Neuroanatomy, University Medical Center Hamburg-Eppendorf, 20246 Hamburg, Germany
[2]Psychiatry Neuroimaging Branch (PNB), Department of Psychiatry and Psychotherapy, University Medical Center Hamburg-Eppendorf, 20246 Hamburg, Germany
[3]Present address: Department of Neuroscience, University of Helsinki, Helsinki 00014, Finland
[4]Present address: Department of Neurophysiology and Pathophysiology, University Medical Center Hamburg-Eppendorf, 20246 Hamburg, Germany
[5]Present address: Center for Neural Circuits and Behavior, Department of Neurosciences, University of California, San Diego, La Jolla, CA 92093, USA
[6]Present address: Centre for Psychiatry and Psychotherapy, Justus Liebig University, Giessen, Germany
[7]Lead Contact
*Correspondence: hangop@zmnh.uni-hamburg.de
https://doi.org/10.1016/j.neuron.2019.09.042

SUMMARY

Cognitive deficits, core features of mental illness, largely result from dysfunction of prefrontal networks. This dysfunction emerges during early development, before a detectable behavioral readout, yet the cellular elements controlling the abnormal maturation are still unknown. Here, we address this open question by combining *in vivo* electrophysiology, optogenetics, neuroanatomy, and behavioral assays during development in mice mimicking the dual genetic-environmental etiology of psychiatric disorders. We report that pyramidal neurons in superficial layers of the prefrontal cortex are key elements causing disorganized oscillatory entrainment of local circuits in beta-gamma frequencies. Their abnormal firing rate and timing relate to sparser dendritic arborization and lower spine density. Administration of minocycline during the first postnatal week, potentially acting via microglial cells, rescues the neuronal deficits and restores pre-juvenile cognitive abilities. Elucidation of the cellular substrate of developmental miswiring causing later cognitive deficits opens new perspectives for identification of neurobiological targets amenable to therapies.

INTRODUCTION

Cortical function relies on the precise wiring and activation of diverse populations of pyramidal cells and interneurons that are entrained in oscillatory rhythms. Although recent studies have revealed several assembling rules of cortical microcircuits in the adult brain (Harris and Shepherd, 2015), their ontogeny is still poorly understood. Given the uniqueness of the developing brain in its spatial and temporal organization of coordinated activity (Brockmann et al., 2011; Khazipov et al., 2004), the depolarizing action of GABA (Kirmse et al., 2015) and the formation of transient connectivity patterns (Marques-Smith et al., 2016), the functional coupling within immature microcircuits is likely to bear equally unique traits. Elucidating the features of such immature networks is of paramount importance in the context of neurodevelopmental disorders, as their early disruption is thought to underlie the later emergence of devastating symptoms that characterize these diseases (Marín, 2016).

We started to elucidate the mechanisms of functional coupling within the developing brain and have shown that pyramidal neurons in the superficial layers of the prefrontal cortex (PFC) play a fundamental role in generating beta/low-gamma oscillations in the neonatal mouse (Bitzenhofer et al., 2017). At adulthood, coordinated activity in gamma-frequency band is instrumental to cognitive processing (Bosman et al., 2014) and relates to the pathophysiology of psychiatric disorders (Cho et al., 2015; Uhlhaas and Singer, 2015). Disturbed gamma activity has been observed long before the onset of psychosis in high-risk humans (Leicht et al., 2016) and during neonatal development in animal models (Hartung et al., 2016). However, the circuit dysfunction underlying such abnormalities is still unknown.

To address this knowledge gap, we interrogate the developing prefrontal network in a mouse model mimicking both the genetic (mutation of the intracellular hub of developmental processes Disrupted-In-Schizophrenia 1 [DISC1] gene; Brandon and Sawa, 2011) and the environmental (challenge by maternal immune activation [MIA]) background that has been related to mental illness (dual-hit genetic-environmental [GE] mice). At adult age, these mice mimic, to a large extent, the network dysfunction as well as memory and attention deficits identified in human psychiatric disorders (Abazyan et al., 2010). The impairment of prefrontal-hippocampal circuits underlying

 © 2019 The Authors. Published by Elsevier Inc.
This is an open access article under the CC BY license (http://creativecommons.org/licenses/by/4.0/).

poorer cognitive performance emerges early in life only when both risk factors converge and is absent in neonatal mice challenged with the genetic or environmental stressor alone (Hartung et al., 2016). To elucidate the mechanisms of developmental dysfunction, we focus on neonatal age (end of 1st–beginning of 2nd postnatal week) of rodents that roughly corresponds to the second/third trimester of human pregnancy, a period of high vulnerability for mental disorders (Selemon and Zecevic, 2015). We combine *in vivo* and *in vitro* electrophysiology with optogenetics, pharmacology, behavioral testing, and confocal microscopy-based structural investigations of the prelimbic subdivision (PL) of the prefrontal cortex. We show that pyramidal neurons in superficial layers exhibit major morphological, synaptic, and functional deficits and lack the ability to organize the beta-gamma entrainment of local prelimbic circuits in neonatal dual-hit GE mice, while deep layers neurons are largely unaffected. Transient administration of minocycline, potentially modulating microglia inflammatory response (Kobayashi et al., 2013), rescues electrophysiological and structural deficits, as well as cognitive abilities at juvenile age. Moreover, we propose that early disruption of prefrontal networks might be predictive of memory impairment at juvenile age.

RESULTS

Layer- and Frequency-Specific Dysfunction of Local Circuits in the Prelimbic Cortex of Dual-Hit GE Mice

To get first insights into the source of prelimbic dysfunction in dual-hit GE mice, we performed extracellular recordings of the local field potential (LFP) and multiple-unit activity (MUA) over prelimbic layers using four-shank 16 site electrodes in lightly anesthetized (Chini et al., 2019) postnatal day (P) 8–10 control (n = 38 pups from 13 litters) and GE mice (n = 18 pups from 6 litters). This developmental stage corresponds to the initiation of hippocampus-driven entrainment of prelimbic circuitry (Ahlbeck et al., 2018; Brockmann et al., 2011). The exact position of recording sites covering superficial and deep layers was confirmed by the reconstruction of electrode tracks *post mortem* (Figure 1A). In line with our previous findings (Bitzenhofer et al., 2015; Brockmann et al., 2011; Cichon et al., 2014; Hartung et al., 2016), the first patterns of network activity in the neonatal PL of all investigated control and dual-hit GE mice were discontinuous, i.e., spindle-shaped oscillations switching between theta and beta-gamma frequency components alternated with long periods of network silence (Figure 1B). The firing of prelimbic neurons was strongly timed by the oscillatory rhythms. As previously reported (Chini et al., 2019), the patterns of network oscillations and neuronal firing in the PL were similar in urethane-anesthetized and non-anesthetized neonatal pups, yet the magnitude of activity decreased in the presence of anesthesia (Figure S1). The similarities might be due to the ability of urethane to mimic sleep conditions (Clement et al., 2008), the dominant behavioral state of neonatal mice (Cirelli and Tononi, 2015). Although dual-hit GE mice have been reported to have profoundly altered network activity and neuronal firing at neonatal age when compared with controls (Hartung et al., 2016), it is still unclear whether the dysfunction equally affects the local prelimbic circuits. To address this question, we first monitored the layer-specific differences between oscillatory patterns of control and dual-hit GE mice. Major differences in the occurrence, duration, and broadband power of oscillatory events were detected when comparing the two groups of mice (Figures 1C, 1D, and S2A–S2E; Table S1). However, these detected differences were similar across layers. This might be due, on the one hand, to a layer-unspecific overall damping of entrainment in dual-hit GE mice and, on the other hand, to non-specific conduction synchrony within a rather small tissue volume (300- to 400-μm radius). To discriminate between the two sources, in a second step, we investigated the layer-specific firing rate and timing by oscillatory phase, which are not contaminated by non-specific volume conduction. The firing of neurons in prelimbic superficial layers in GE mice (log -2.1 ± 0.1 spikes/s) was significantly ($p < 10^{-7}$) reduced when compared to controls (0.61 ± 0.04 spikes/s; Figure 1E). In contrast, neurons in deep layers similarly fired in control (-0.95 ± 0.05 spikes/s) and GE mice (-1.3 ± 0.2 spikes/s). The timing of neuronal firing in relation to beta (12–30 Hz) and gamma (30–100 Hz) frequency was also disturbed and lost its precision in superficial layers ($p < 1 \times 10^{-4}$ and $p = 0.021$, respectively), but not deep-layer neurons of GE mice when compared to controls (Figures 1F–1H). The timing of spiking by theta oscillations in both superficial and deep layers was similar in control and dual-hit GE mice (Figure S2E). To verify that our results were not biased by anesthesia, we recorded a set of non-anesthetized P8–P10 control (n = 16) and GE (n = 18) mice and confirmed that GE mice have reduced broadband LFP power. The decreased MUA and single-unit activity (SUA) firing rates were limited to neurons in superficial layers (Figures S1G–S1L). In contrast to the significant perturbation of prelimbic activity in neonatal dual-hit GE mice, the oscillatory and firing patterns of one-hit genetic (G) (i.e., only DISC1) or environmental (E) (i.e., only MIA) mice were similar to those of control pups (Figures S2F–S2H). Furthermore, the layer-specific dysfunction seems to be characteristic to the investigated developmental stage P8–P10. In mice of 4–6 days of age, a time window in which neurons are still migrating (Ignacio et al., 1995), deficits are present, yet the layer specificity is lacking (Figures S2I–S2K). On the other hand, in line with our previous data (Hartung et al., 2016), the properties of prelimbic network oscillations were similar in control and dual-hit GE mice at pre-juvenile age (P20–P23; Figures S2L–S2N).

These results demonstrate abnormal beta-gamma band oscillations and entrainment of superficial layers of PL in dual-hit GE mice during this defined developmental period (P8–P10).

Beta-Gamma Band Dysfunction of Prelimbic Circuits in Dual-Hit GE Mice Results from Abnormal Activation of Superficial Layers Pyramidal Neurons

In developing circuits, beta-gamma band oscillatory activity has been recently shown to require the activation of pyramidal neurons in superficial ($PYRs_{SUP}$), but not deep, layers ($PYRs_{DEEP}$) of PL (Bitzenhofer et al., 2017). Therefore, the weaker beta-gamma entrainment of prelimbic circuits and coupling of neuronal firing to fast oscillations identified in GE mice might result from dysfunction of $PYRs_{SUP}$. To test this hypothesis, we monitored the effects of light activation of prelimbic neurons

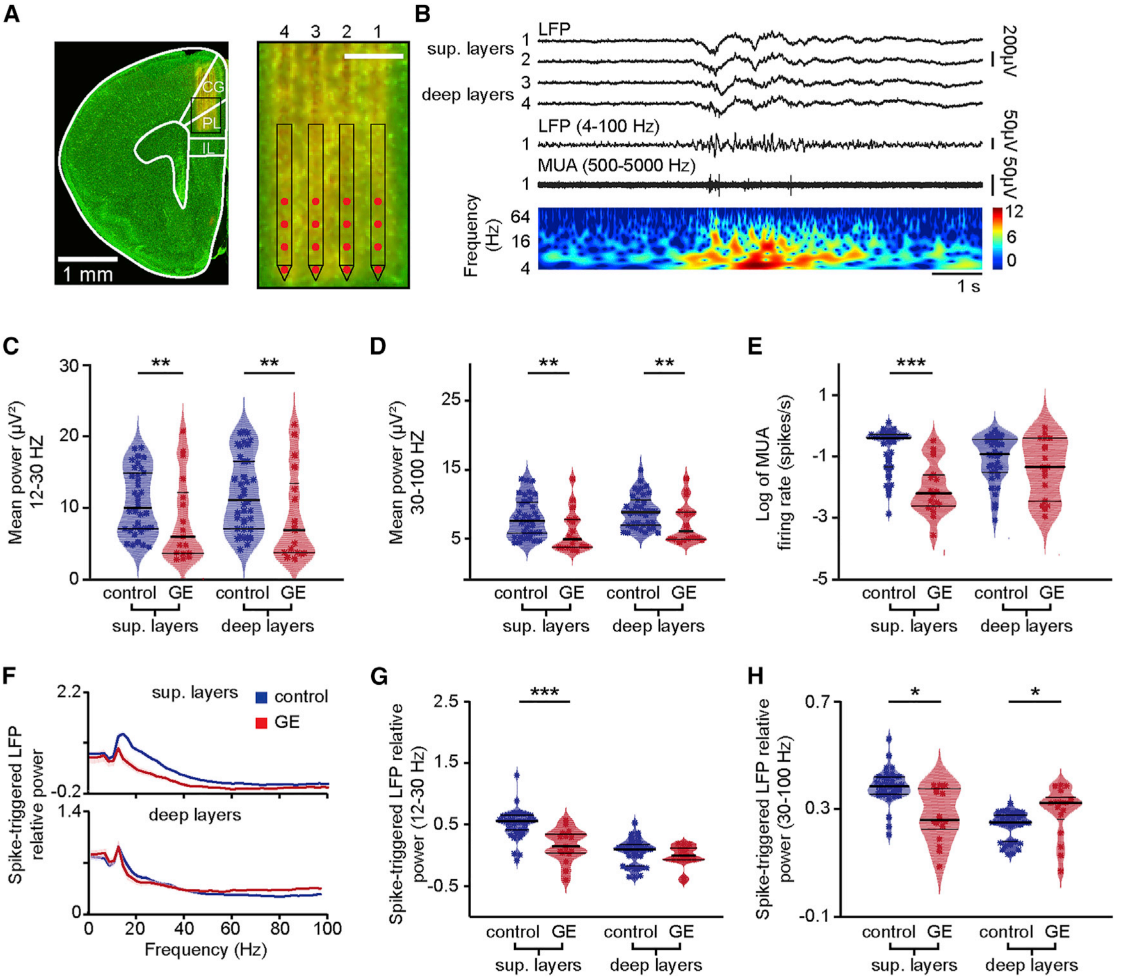

Figure 1. Abnormal Patterns of Discontinuous Oscillatory Activity and Neuronal Firing over the Layers of Prelimbic Cortex of Neonatal Dual-Hit GE Mice

(A) Digital photomontage reconstructing the position of a 4-shank DiI-labeled recording electrode in the PL of a Nissl-stained 100-µm-thick coronal section (green) from a P9 mouse. Inset, the position of recording sites (red) over the prelimbic layers is displayed at higher magnification. Scale bar, 200 µm.

(B) Characteristic discontinuous oscillatory activity recorded in superficial and deep layers of PL before (top) and after band pass (4–100 Hz) filtering (middle; recording site 1 in superficial layers) and the corresponding MUA after band pass (500–5,000 Hz) filtering (bottom; recording site 1 in superficial layers). Color-coded frequency plot shows the wavelet of the LFP (recording site 1) at identical timescale.

(C) Violin plot displaying the power in beta frequency band of oscillations in superficial and deep layers of the prelimbic cortex of control (blue; n = 38) and GE (red; n = 18) mice.

(D and E) Same as (C) for the power in gamma frequency band (D) and MUA firing rate (E).

(F) Plots of frequency-dependent relative power of spike-triggered LFP in superficial (top) and deep layers (bottom) of control (blue) and GE (red) mice.

(G) Violin plot displaying the relative power of spike-triggered LFP in beta band for superficial and deep layers of control (blue; n = 38) and GE (red; n = 18) mice.

(H) Same as (G) for the LFP in gamma band.

For (C)–(E), (G), and (H), data are presented as median with 25th and 75th percentile, and single data points are shown as asterisks. The shaded area represents the probability distribution of the variable. *$p < 0.05$, **$p < 0.01$, and ***$p < 0.001$; analysis of covariance (ANCOVA) with age as covariate (C–E) and Yuen's bootstrap test (G and H) with 20% level of trimming for the mean.

that were transfected with light-sensitive proteins and the red fluorescent protein tDimer2. Using our recently established protocol for optogenetic manipulation of developing circuits (Bitzenhofer et al., 2017), we achieved cell-type-, layer-, and area-specific transfection of neurons by *in utero* electroporation (IUE) (Figures S3A and S3B). Constructs coding for the double mutant channelrhodopsin E123 T159 (ChR2(ET/TC)) were transfected by IUE at embryonic day (E) 15.5 and E12.5 for selective targeting of superficial and deep layers, respectively (Figures S3C and S3D). Staining for NeuN showed that a similar fraction of neurons was transfected in control (34.7% ± 0.8%; n = 13 pups) and GE mice (32.0% ± 0.7%; n = 8 pups). The pyramidal-like shape and orientation of primary dendrites confirmed that the expression constructs were exclusively integrated into cell lineages of pyramidal neurons. Omission of ChR2(ET/TC) from the expression construct (i.e., opsin-free) yielded similar

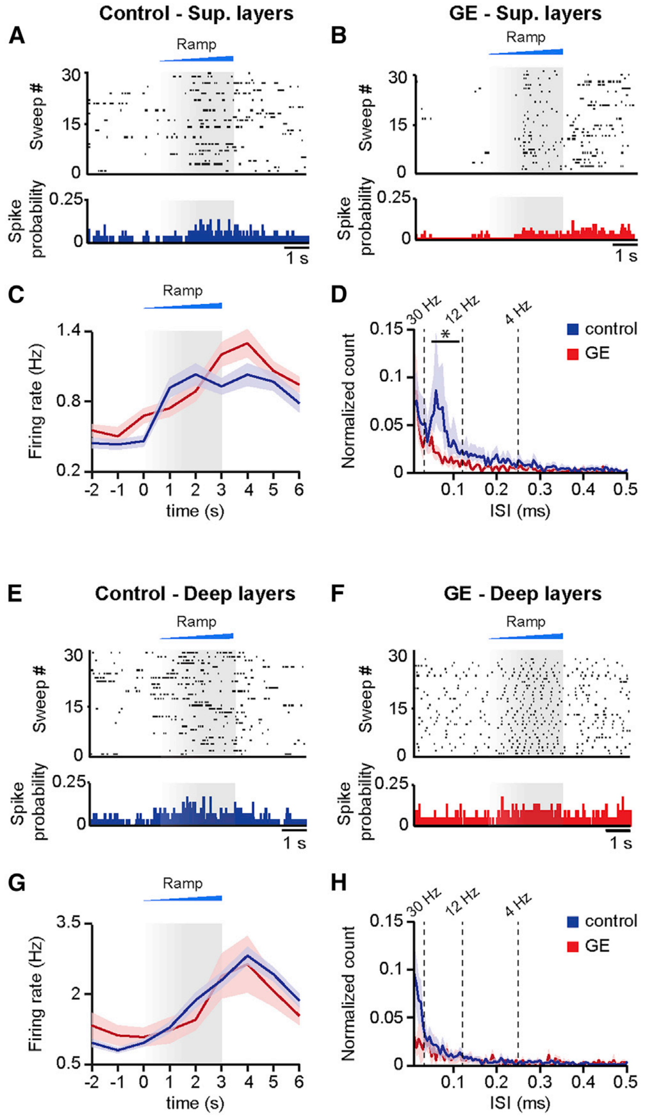

Figure 2. Firing Patterns after Optogenetic Activation of $PYRs_{SUP}$ and $PYRs_{DEEP}$ in Control and Dual-Hit GE Mice *In Vivo*

(A) Representative raster plot and corresponding spike probability histogram displaying the firing of a $PYRs_{SUP}$ from a control mouse in response to 30 sweeps of ramp stimulation (473 nm; 3 s).

(B) Same as (A) for transfected $PYRs_{SUP}$ from GE mice.

(C) Line plot displaying the mean MUA firing rate in transfected $PYRs_{SUP}$ of control (blue; n = 43 recording sites from 13 pups) and GE (red; n = 40 recording sites from 10 mice) mice in response to ramp illumination.

(D) Same as (C) for inter-spike interval within 10- to 500-ms range normalized to all ISIs.

(E–H) Same as (A)–(D) for transfected $PYRs_{DEEP}$ from control (n = 116 recording sites from 13 pups) and GE mice (n = 27 recording sites from n = 6 pups). Data are presented as mean ± SEM. *$p < 0.05$; linear mixed-effect model with animal as a random effect.

expression rates and distribution of tDimer2-positive neurons. Moreover, the success rate of transfection by IUE was similar in control and dual-hit GE mice in the presence and absence of opsin (Figure S3E).

The transfection procedure by IUE had no major effects on the overall development of animals (Figures S3F–S3K). Although IUE caused significant reduction of litter size in both control and GE mice (non-electroporated: 8.3 ± 1.1 pups/litter; IUE: 4.6 ± 1.3 pups/litter; p = 0.03), all investigated pups had similar body length, tail length, and weight during the early postnatal period. Vibrissa placing, surface righting, and cliff aversion reflexes were also not affected by IUE or transfection of neurons with opsins (Figures S3I–S3K).

First, we assessed the efficiency of light stimulation in inducing action potentials (APs) in prelimbic neurons of control and dual-hit GE mice *in vitro*. For this, whole-cell patch-clamp recordings were performed from tDimer2-positive $PYRs_{SUP}$ (n = 42) and $PYRs_{DEEP}$ (n = 38) in coronal slices containing the PL from P8–P10 mice after IUE at E15.5 and E12.5, respectively. In line with the previously reported "inside-out" pattern of cortical maturation and, correspondingly, the more mature profile of neurons in deep versus superficial layers, $PYRs_{SUP}$ and $PYRs_{DEEP}$ in control mice significantly differed in some of their passive and active membrane properties (Bitzenhofer et al., 2017). However, in dual-hit GE mice, the resting membrane potential of $PYRs_{SUP}$ (−53.2 ± 0.37 mV) was more positive when compared with controls (−63.2 ± 0.3 mV; $p = 2 \times 10^{-4}$), and the maximum amplitude of action potentials decreased (44.8 ± 0.80 mV versus 29.2 ± 0.36 mV in controls; p = 0.018). These alterations of intrinsic neuronal properties might point to the immaturity of $PYRs_{SUP}$ in GE mice, even though membrane resistance, membrane time constant, and action potential half-width were not significantly different across conditions (Figures S4A–S4E). The passive and active properties of ChR2(ET/TC)-transfected neurons were similar to those previously reported for age-matched mice (Bitzenhofer et al., 2017). Pulsed light stimulation (3 ms, 473 nm, 5.2 mW/mm^2) depolarized transfected fluorescently labeled neurons and led to robust firing in all pups. The probability of triggering APs by pulsed light stimuli decreased with increasing stimulation frequency, yet it differed in its dynamics in control versus GE mice. Whereas $PYRs_{SUP}$ of control mice were able to reliably follow light stimulations up to 16 Hz, in GE mice, they had a significant firing drop already between 8 and 16 Hz (Figures S4E and S4F). Light stimulation of $PYRs_{DEEP}$ showed a similar decrease of firing probability with augmenting stimulation frequency in control and GE mice.

To elucidate the consequences of abnormal intrinsic firing preference for oscillatory network entrainment, we monitored the effects of light activation of either $PYRs_{SUP}$ or $PYRs_{DEEP}$ *in vivo*. In controls, activation of $PYRs_{SUP}$ selectively drove the neonatal prelimbic networks in beta-gamma frequency range, whereas activation of $PYRs_{DEEP}$ caused non-specific network activation. We reasoned that, if $PYRs_{SUP}$ are indeed the cause of the previously demonstrated disruption of beta-gamma activity in the PL of GE mice, then their light stimulation *in vivo* should not be able to selectively induce oscillations in this range.

Ramp light stimulation increased the neuronal firing of ChR2(ET/TC)-transfected $PYRs_{SUP}$ and $PYRs_{DEEP}$ in control and GE mice ($p < 10^{-4}$ for all conditions), but not of neurons transfected with opsin-free constructs (Figures 2A–2H and S5).

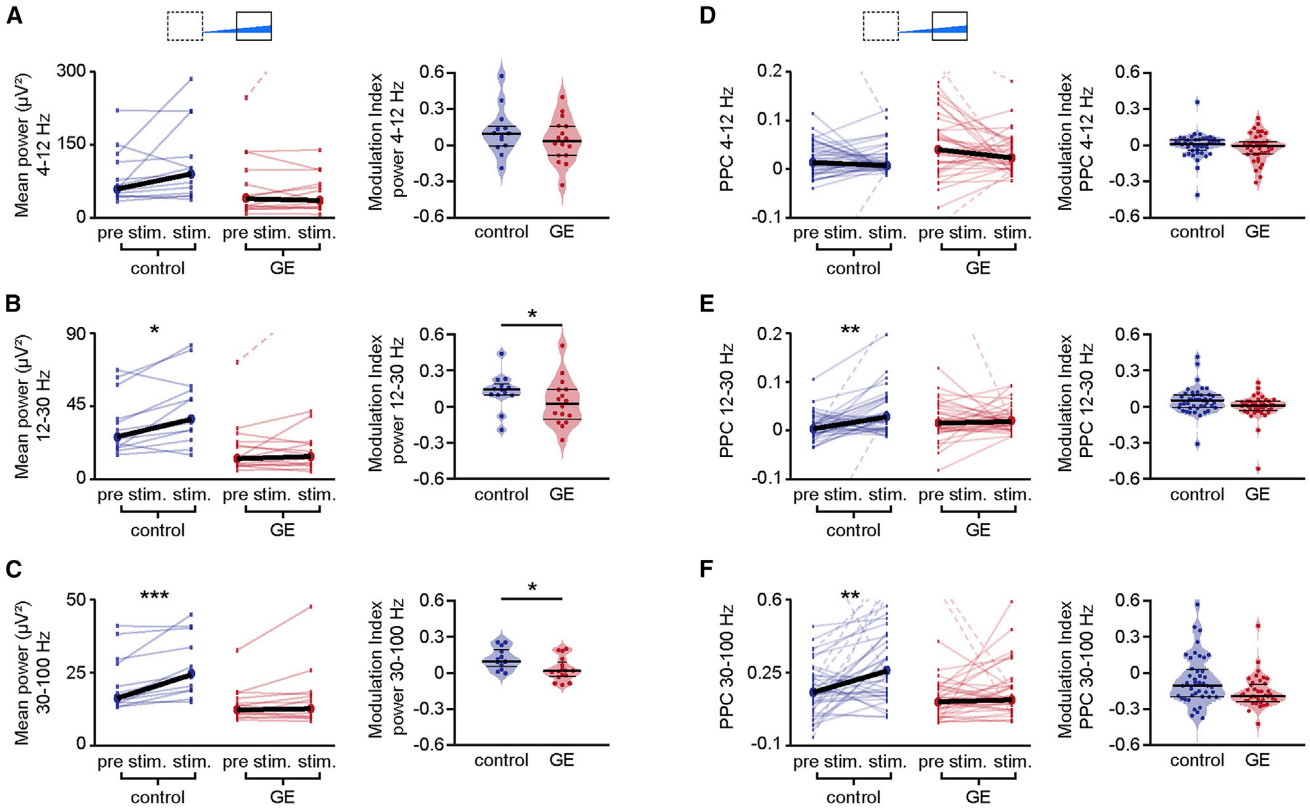

Figure 3. Network Activity after Optogenetic Activation of PYRs$_{SUP}$ in Control and Dual-Hit GE Mice *In Vivo*

(A) Left: scatterplot displaying the LFP power in the theta (4–12 Hz) frequency band for control (blue; n = 13) and GE (red; n = 16) mice before (pre stim.; 1.5 s) and during the second half (stim.; 1.5 s) of ramp stimulation. Right: violin plot displaying the stimulation modulation index of light-induced LFP power in the theta frequency band for control and GE mice is shown.

(B and C) Same as (A) for beta (12–30 Hz) and gamma (30–100) frequency bands.

(D–F) Same as (A)–(C) for pairwise phase consistency (PPC) of PYRs$_{SUP}$ in control (n = 43 recording sites from 13 pups) and GE (n = 40 recording sites from 10 pups) mice.

In scatterplots (A–F), data are presented as median, and individual values are displayed as thin dots and lines. In violin plots (A–F), data are presented as median with 25th and 75th percentile, and single data points are shown as asterisks. *p < 0.05, **p < 0.01, and ***p < 0.001; Yuen's bootstrap test (A–F) with 20% level of trimming for the mean and linear mixed-effect model with animal as a random effect (D–F).

The light-induced augmentation of firing was similar in the two groups of mice (p = 0.46 and p = 0.24 for PYRs$_{SUP}$ and PYRs$_{DEEP}$, respectively). The spike discharge initiated once the power exceeded a certain threshold. For some neurons, the firing decreased toward the end of the ramp stimulations, indicating that, similar to the *in vitro* conditions, their membrane potential reached a depolarizing plateau, preventing further spiking. However, for the majority of neurons, the firing rate after stimulus remained higher than before the stimulus (Figures 2C and 2G), suggesting that global network activation had been induced by light stimulation in the developing circuits. Major differences in the firing of prelimbic neurons from control and GE mice were detected. Although PYRs$_{SUP}$ in controls had a preferred inter-spike interval of ~60 ms, equivalent to a population firing at 16.7 Hz (Figures 2A and 2D), a coordinated frequency-tuned discharge pattern was absent in GE mice upon ramp stimulation of PYRs$_{SUP}$ (condition effect, p = 4 × 10^{-5}; p < 0.05 in the 15- to 20-Hz range with the exception of p = 0.059 at 16.7 Hz; Figures 2B and 2D). In contrast, the firing dynamics of PYRs$_{DEEP}$ was similar in control and GE mice (condition effect p = 0.11) and showed no frequency-specific concentration of firing during ramp stimulation (Figures 2E–2H).

To causally prove the contribution of abnormal firing of PYRs$_{SUP}$ to the weaker beta-gamma band entrainment previously identified in the PL of dual-hit GE mice, we tested the effects of ramp stimulations on the discontinuous network oscillations. When compared with pulsed stimulations, ramp stimulations have the advantage of not inducing power contamination by repetitive and fast large-amplitude voltage deflections resulting from simultaneous opening of light-activated channels and to trigger more physiological and not artificially synchronous firing patterns (Bitzenhofer et al., 2017). In control mice, the LFP power in beta- and gamma-frequency range significantly increased during ramp stimulation of PYRs$_{SUP}$ (p = 0.02 and p = 0.002, respectively), whereas the theta-band activity remained unaffected (p = 0.26). In contrast, PYRs$_{SUP}$ in GE mice lost their ability to boost neonatal prelimbic oscillations in a frequency-specific manner, because ramp

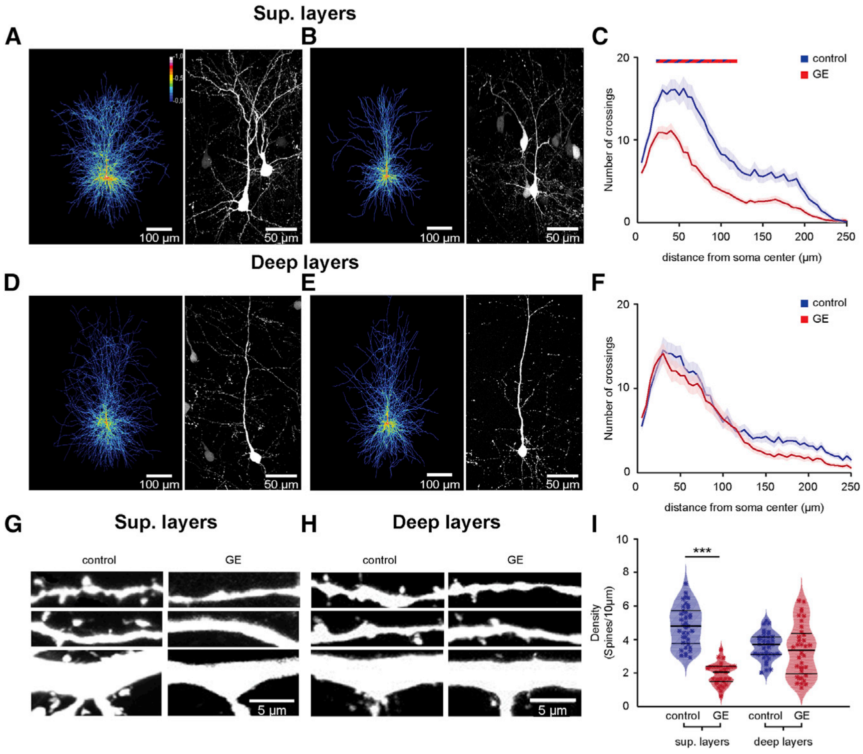

Figure 4. Simplified Dendritic Arborization and Reduced Spine Density in PYRs$_{\mathrm{SUP}}$ of Dual-Hit GE Mice

(A) Left: heatmap displaying an overlay of all traced dendrites of transfected PYRs$_{\mathrm{SUP}}$ in control mice. Right: photograph of a representative PYRs$_{\mathrm{SUP}}$ in a P10 mouse is shown.

(B) Same as (A) for a P10 dual-hit GE mouse.

(C) Graph displaying the average number of dendritic intersections within a 250-µm radius from the soma center of PYRs$_{\mathrm{SUP}}$ in control (blue; n = 21 neurons from 3 pups) and GE (red; n = 21 neurons from 3 pups) mice. Blue/red bar indicates significant difference between control and GE mice.

(D–F) Same as (A)–(C) for PYRs$_{\mathrm{DEEP}}$ from control (blue; n = 21 neurons from 3 pups) and GE (red; n = 21 neurons from 3 pups) mice.

(G) Photograph displays representative basal (top), secondary apical (middle), and proximal oblique and apical (bottom) dendrites of a PYRs$_{\mathrm{SUP}}$ from a P10 control mouse (left) and a P10 GE mouse (right).

(H) Same as (G) for PYRs$_{\mathrm{DEEP}}$.

(I) Violin plot displaying the average spine density on dendrites from PYRs$_{\mathrm{SUP}}$ of control (blue; n = 39 dendrites from 13 neurons) and GE (red; n = 30 dendrites from 10 neurons) mice.

In (C) and (F), data are presented as mean ± SEM. In (I), data are presented as median with 25th and 75th percentile, and single data points are displayed as asterisks. *$p < 0.05$, **$p < 0.01$, and ***$p < 0.001$; linear mixed-effect model with animal (C and F) and neuron (I) as random effects.

stimulations did not affect the LFP power (p = 0.49, p = 0.57, and p = 0.44 for theta-, beta-, and gamma-frequency band, respectively; Figure 3A). Moreover, stimulation of PYRs$_{\mathrm{SUP}}$ differently modulated power in beta- and gamma-frequency band between control and GE mice (p = 0.03 and p = 0.03, respectively). Not only the light-induced inter-spike interval and power of network oscillations were disrupted in GE mice, but also the timing of firing by the oscillatory phase was impaired. To quantify this relationship, we used pairwise phase consistency (PPC), a measure of synchrony that is not biased by firing rates (Vinck et al., 2010). In control mice, stimulation increased the PPC for beta (p = 0.008) and gamma oscillations (p = 0.003), but not for theta (p = 0.24). In contrast, the PPC for theta (p = 0.09), beta (p = 0.86), and gamma oscillations (p = 0.37) during stimulation of PYRs$_{\mathrm{SUP}}$ in GE mice did not change (Figures 3D–3F), indicating that the synchronization of spikes relative to the phase of these oscillations was not affected by light activations of PYRs$_{\mathrm{SUP}}$. However, due to high variability, no difference in PPC modulation between the two mouse groups achieved statistical significance (Figures 3D–3F).

In line with the frequency-unspecific augmentation of firing rate after light activation of PYRs$_{\mathrm{DEEP}}$ in control mice, the LFP power in all frequency bands increased during stimulation and remained at a high level even after it. In GE mice, optogenetic stimulation did not augment the power. No differences in power modulation between the two mouse groups were detected (Figure S6).

Thus, the reduced beta-gamma activity in the PL of neonatal dual-hit GE mice relates to the dysfunction of firing dynamics of PYRs$_{\mathrm{SUP}}$.

Pyramidal Neurons in the Superficial Layers of PL in Neonatal Dual-Hit GE Mice Show Major Morphological and Synaptic Deficits

The selective dysfunction of PYRs$_{\mathrm{SUP}}$ and the corresponding abnormal network activity in GE mice might relate to abnormal morphology and connectivity of these neurons at neonatal age. To test this hypothesis, we undertook a detailed histological examination of the cytoarchitecture of tDimer-labeled pyramidal neurons in superficial and deep layers of P10 control and GE mice. PYRs$_{\mathrm{SUP}}$, but not PYRs$_{\mathrm{DEEP}}$, of GE mice showed a significant reduction in the soma size when compared to neurons of controls (n = 21 neurons for every condition; p = 0.039 for PYRs$_{\mathrm{SUP}}$ and p = 0.95 for PYRs$_{\mathrm{DEEP}}$; Figure S7A). The complexity of dendritic branching was assessed by Sholl analysis of three-dimensionally reconstructed PYRs$_{\mathrm{SUP}}$ and PYRs$_{\mathrm{DEEP}}$. When compared to controls, PYRs$_{\mathrm{SUP}}$ of GE mice had major reduction in dendritic branching (condition effect $p < 1 \times 10^{-9}$; Figures 4A–4C). These deficits were particularly prominent within a radius of 20–115 µm from the cell soma center ($p < 0.05$ for all pairwise comparisons). In accordance with our electrophysiological results, we found no significant differences in the complexity of dendritic arborization for PYRs$_{\mathrm{DEEP}}$ of GE and control mice (condition effect p = 0.56; Figures 4D–4F). Accordingly, the total dendritic branch length was reduced in

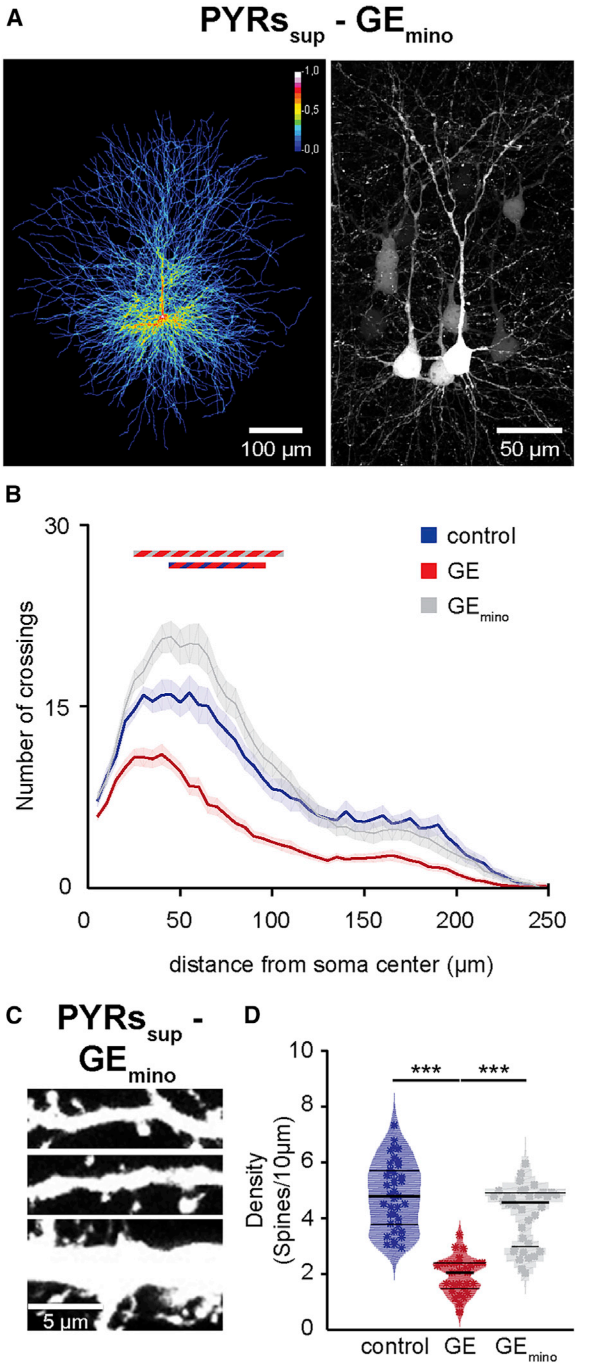

Figure 5. Minocycline Treatment Rescues the Abnormal Structure of PYRs$_{SUP}$ in GE Mice

(A) Left: heatmap displaying an overlay of all traced dendrites of transfected PYRs$_{SUP}$ in GE$_{mino}$ mice. Right: photograph of a representative PYR$_{SUP}$ in a P10 GE$_{mino}$ mouse is shown.

(B) Graph displaying the average number of dendritic intersections within a 250-µm radius from the soma center of PYRs$_{SUP}$ in control (blue; n = 21 neurons from 3 pups), GE (red; n = 21 neurons from 3 pups), and GE$_{mino}$ (gray; n = 21 neurons from 3 pups) mice. Blue/red and gray/red bars indicate significant difference between control and GE mice and GE and GE$_{mino}$ mice, respectively.

(C) Photograph displays representative basal (top), secondary apical (middle), and proximal oblique and apical (bottom) dendrites of a PYR$_{SUP}$ from a P10 GE$_{mino}$ mouse.

(D) Violin plot displaying the average spine density on dendrites from PYRs$_{SUP}$ of control (blue; n = 39 dendrites from 13 neurons), GE (red; n = 30 dendrites from 10 neurons), and GE$_{mino}$ (gray; n = 36 dendrites from 12 neurons) mice. In (B), data are presented as mean ± SEM. In (D), data are presented as median with 25th and 75th percentile, and single data points are displayed as asterisks. ***$p < 0.001$; linear mixed-effect model with animal (B) and neuron (D) as random effects.

PYRs$_{SUP}$, but not PYRs$_{DEEP}$, of GE mice (n = 21 neurons for every condition; p = 0.024 for PYRs$_{SUP}$ and p = 0.37 for PYRs$_{DEEP}$; Figure S7B).

Next, we examined the spine density along the dendrites of PYRs$_{SUP}$ and PYRs$_{DEEP}$, whose dendritic morphology we had previously analyzed. PYRs$_{SUP}$ of GE mice (n = 10 neurons) had significantly lower density when compared to controls (n = 13 neurons; condition effect $p = 7 \times 10^{-4}$), whereas the values were comparable for PYRs$_{DEEP}$ of control (n = 9 neurons) and GE mice (n = 9 neurons; condition effect p = 0.75; Figures 4G–4I). The magnitude of density reduction was similar for different types of dendrites (apical and proximal oblique dendrites, secondary apical dendrites, and basal dendrites; condition effect $p = 7 \times 10^{-4}$, $p = 5 \times 10^{-4}$, and p = 0.001, respectively; Figures S7C–S7E). In line with the network dysfunction, the prominent morphological/structural deficits seem to be largely confined to neonatal age. PYRs$_{SUP}$ of pre-juvenile (P21) GE mice had a normal dendritic arborization (n = 28 neurons; condition effect p = 0.99) and spine density (n = 16 neurons; condition effect p = 0.3). Only soma size and total dendritic path length were slightly decreased, yet not at significance level (n = 28 neurons; p = 0.088 and p = 0.055, respectively; Figures S7F–S7J).

The simplified dendritic arborization and the decreased spine density of PYRs$_{SUP}$, but not PYRs$_{DEEP}$, further confirm the layer-specific dysfunction in neonatal dual-hit GE mice.

Transient Minocycline Administration Rescues Prelimbic Deficits in Dual-Hit GE Mice

We next set out to determine whether the morphological and functional deficits of PYRs$_{SUP}$ in the PL of GE mice could be rescued during early development. Minocycline is a tetracycline antibiotic that exerts a variety of functions and has anti-inflammatory properties (Garrido-Mesa et al., 2013). Minocycline has recently shown promising results as an adjunct drug to treat depression (Emadi-Kouchak et al., 2016), bipolar disorder (Savitz et al., 2018), and schizophrenia (Zhang et al., 2018) and even to delay or prevent the incidence of schizophrenia (Sellgren et al., 2019). However, in the absence of mechanistic insights, its therapeutic potential remains controversial (Deakin et al., 2018; Kishimoto et al., 2018).

We administered minocycline from P1 to P8 by adding it to the drinking water of the dam (Dansie et al., 2013; Luzi et al., 2009) and analyzed the morphological, functional, and behavioral consequences in P8–P10 pups. First, Sholl analysis of three-dimensionally reconstructed tDimer-positive PYRs$_{SUP}$ (n = 21 neurons) from GE$_{mino}$ mice showed that the complexity of dendritic branching was fully restored after treatment, being

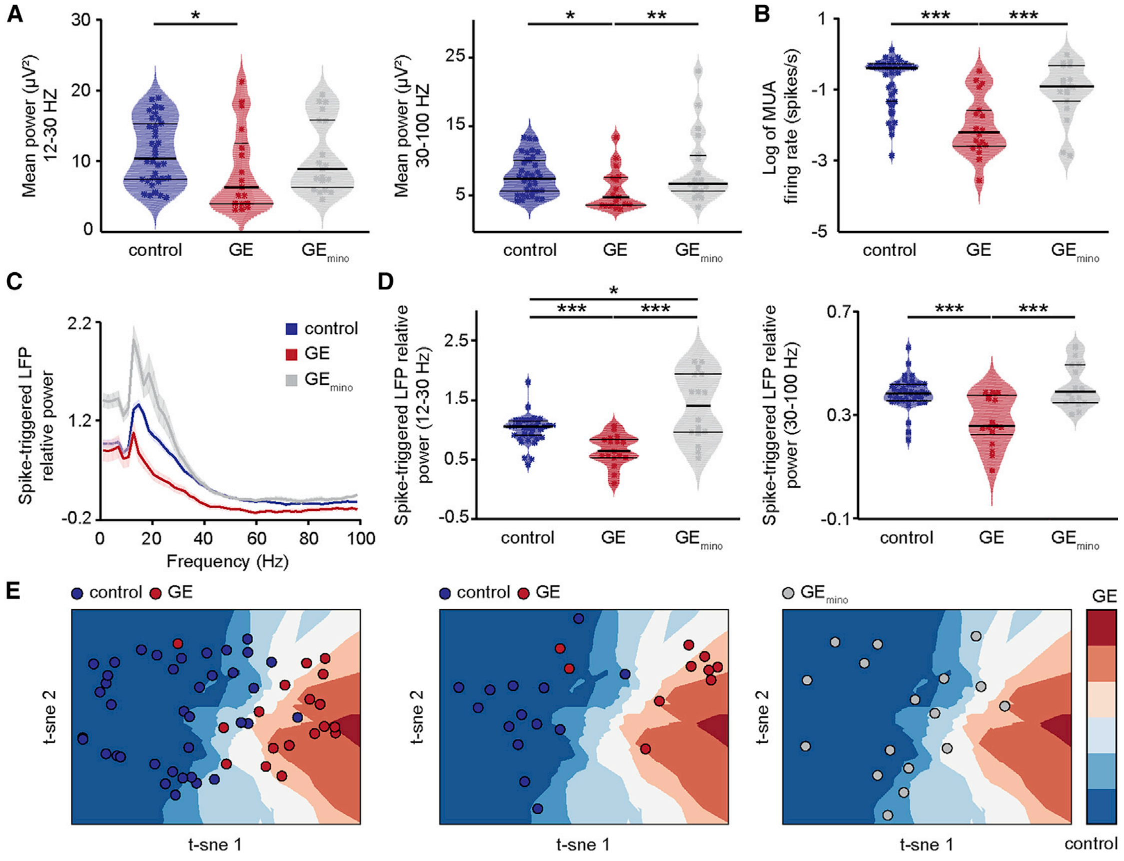

Figure 6. Minocycline Treatment Rescues Electrophysiological Core Dysfunctions in Dual-Hit GE Mice

(A) Violin plot displaying the beta (left) and gamma (right) band power of oscillations in superficial layers of the PL of control (blue; n = 38), GE (red; n = 18), and GE_{mino} (gray; n = 18) mice.

(B) Same as (A) for MUA firing rate.

(C) Plots of frequency-dependent relative power of spike-triggered LFP in superficial layers (top) of control (blue), GE (red), and GE_{mino} (gray) mice.

(D) Violin plot displaying the relative power of spike-triggered LFP in beta (left) and gamma (right) band for superficial layers of control (blue; n = 38), GE (red; n = 18), and GE_{mino} (gray; n = 18) mice.

(E) t-Distributed stochastic neighbor embedding (T-SNE) plot of superficial layers electrophysiological features of control (blue dots) and GE (red dots) mice in the training/cross-validation (left) and test (middle) set and GE_{mino} (gray dots; right).

The background represents an approximation of the decision space of the classifier. *p < 0.05, **p < 0.01, and ***p < 0.001; ANCOVA with age as covariate (A and B) and Yuen's bootstrap test (D) with 20% level of trimming for the mean.

similar to that of controls (condition effect p = 0.77; p > 0.05 for all pairwise comparisons; Figures 5A and 5B). Minocycline treatment rescued the synaptic deficits too. $PYRs_{SUP}$ from GE_{mino} mice (n = 12 neurons) had a similar spine density as those from control mice (condition effect p = 0.78) that was significantly increased when compared to GE mice (condition effect p = 5×10^{-5}; Figures 5C and 5D). The effect was similar across the different types of dendrites that were analyzed.

Second, we assessed the properties of prelimbic network oscillations and neuronal firing in GE_{mino} mice and compared them with those from control and GE mice. The power in beta and gamma band of prelimbic oscillations recorded in superficial layers was similar in control and minocycline-treated GE mice (p = 0.90 and p = 0.31, respectively; Figures 6A and 6B; Table S1). Similarly, the prelimbic firing rate and timing by oscillatory phase were rescued (Figures 6C and 6D). The firing rate of neurons in superficial layers was similar for controls (log values −0.61 ± 0.04) and GE_{mino} mice (log values −0.9 ± 0.1; p = 0.59). The timing of prelimbic firing in superficial layers of GE_{mino} mice, as measured by spike-triggered LFP power, was rescued (gamma band; p = 0.48) or even slightly increased (beta band; p = 0.026) when compared to controls (Figure 6D). In contrast to the profound changes observed in superficial layers after minocycline treatment, the network activity and neuronal firing in deep layers of PL from GE_{mino} mice remained largely unaffected (Figures S8A–S8F). Moreover, the neuronal and network properties in control mice ($control_{mino}$ n = 12) did not change after minocycline administration. Theta, beta, and gamma power of prelimbic oscillations as well as firing rate and spike-triggered relative LFP power in superficial layers were similar in controls and $control_{mino}$ (p = 0.68, p = 0.95, p = 0.36, p = 0.16, p = 0.1, and p = 0.06, respectively). The activity in deep layers was also largely unaffected, with only gamma power being significantly increased (p = 0.006; Figures S8G–S8L).

These data indicate that the abnormal firing and network coupling patterns in the PL of dual-hit GE mice are rescued by administration of minocycline during a defined developmental period.

Electrophysiological Features of Prelimbic Superficial Layers Are Sufficient to Distinguish Control from GE Mice

To test the robustness of conclusions above, we developed a machine-learning classification algorithm (k-nearest neighbors classification), to which we asked to predict whether mice belonged to the control or the GE group (Figure 6E). As input features, we used only the electrophysiological features characterized for neonatal PL: LFP power in beta- and gamma-frequency bands and firing rates of neurons in superficial layers and their spike-triggered LFP power in beta- and gamma-frequency bands. We first used 3-fold cross-validation and iteratively (n = 500) split the dataset of mice (n = 56 mice) into a training (n = 38 mice) and a cross-validation (n = 18) set. The training set was used to tune the algorithm hyper-parameters (further using 3-fold cross-validation), whereas the assessment of the prediction quality was carried out on the cross-validation set. By these means, we were able to obtain a median classification accuracy of 83% on the cross-validation set, thereby showing that superficial-layers-derived features are valid predictors for this classification task (Figure 6E, left). To confirm the robustness and generalizability of our findings, we tested the predictions of the pre-trained k-nearest neighbors classifier on an entirely new dataset (n = 24; test dataset), to which it had not been exposed during the training phase. On the test dataset, the machine-learning classification achieved high classification accuracy (median 80%; Figure 6E, middle). Moreover, when we asked the algorithm to predict to which class GE_{mino} mice belonged to, on average, all but one of them (94%) were classified as belonging to the control group (Figure 6E, right). These data show that superficial-layers-derived electrophysiological features are strong and robust predictors for distinguishing control and GE mice and further confirm the efficacy of the minocycline-administration rescue.

Transient Minocycline Treatment Rescues Abnormal Microglia Function in Dual-Hit GE Mice

Minocycline has been shown to block the stress-induced inflammatory responses of microglia (Kobayashi et al., 2013) and to reduce microglia overpruning in schizophrenic-patients-derived induced microglia-like cells (Sellgren et al., 2019). Therefore, their modulation might represent a possible mechanism explaining the observed minocycline effects. Microglia are key players during early brain development and have been reported to control synapse formation (Miyamoto et al., 2016) and to sculpt the developing circuits by engulfing and remodeling synapses in an activity-dependent manner (Schafer et al., 2012; Weinhard et al., 2018). Transient perturbations in the development of microglia, such as those induced by maternal immune activation (MIA), have far-reaching effects on adult neuronal function and behavior (Shin Yim et al., 2017) that have been linked to mental illness.

In accordance with this stream of evidence, microglia in the PL of neonatal GE mice are profoundly perturbed. When compared with controls, not only was microglia number significantly augmented (+47%; $p < 1 \times 10^{-5}$), but also morphological features, such as area and cell spread, were likewise significantly increased by 29% (n = 1,250 cells for control; n = 1,173 cells for GE mice; $p < 1 \times 10^{-6}$) and 25% ($p < 1 \times 10^{-13}$), respectively (Figures 7A–7D). Moreover, microglia cell perimeter and roundness, but not eccentricity, were also substantially changed in dual-hit GE mice (Figures 7E–7G). These deficits were observed throughout the entire prelimbic cortex and had no layer specificity. Although minocycline-treated GE (GE_{mino}) mice had no reduction in the number of microglial cells (−13%; p = 0.17), microglia showed a reduced area (−35%; n = 1,614 cells; p = 0.015) and cell spread (−11%; $p = 8 \times 10^{-4}$) when compared to GE mice (Figures 7A–7D).

To get insights into the mechanisms that enable microglia to control neuronal function in developing PL, we quantified microglia phagocytosis of pre-synaptic terminals, identified as VGLUT-1-positive puncta. Quantitative analysis revealed that, in GE mice (n = 52 cells), both the number as well as the volume of engulfed VGLUT-1-positive puncta were increased in comparison to control (n = 54 cells; p = 0.006 and p = 0.006, respectively) and, to a less amount, to GE_{mino} (n = 56 cells; p = 0.084 and p = 0.084, respectively) mice. In contrast, there was no difference between controls and GE_{mino} mice (p = 0.287 and p = 0.296, respectively; Figures 7H–7K). High-definition morphological analysis confirmed that GE mice have over all prelimbic layers microglia cells with larger distal volume (condition effect p = 0.019) in comparison to GE_{mino} (p = 0.008) and, to a lesser extent, to controls (p = 0.072).

These data confirm that minocycline has an effect on microglia cells and that it partially restores the phenotype of these cells in GE mice. Although minocycline is a pleiotropic drug, part of its effect on GE mice might therefore be mediated by microglia modulation.

Dysfunction of Prelimbic Superficial Layers and Its Rescue Relates to Later Cognitive Performance

Previous investigations showed that compromised function of PFC in neonatal dual-hit GE mice has behavioral impact on later cognitive abilities. In line with these results, we monitored the novelty detection and recognition memory, which have been shown to rely on functional communication within prefrontal-hippocampal networks. Novel object recognition (NOR) and recency recognition (RR) are based on the innate preference of mice to explore novel or less familiar objects over more familiar ones (Figures 8A and 8C). Therefore, their testing requires no prior training or deprivation and can be achieved shortly after full maturation of sensory and motor abilities (i.e., P17 to P18). All three groups of mice, control, GE, and GE_{mino} were tested using a custom-design arena and objects of different size, color, and texture. We quantified the relative amount of time spent interacting with the novel/less recent object when compared to the familiar/more recent one (discrimination ratio), as well as the relative duration of single interactions with the two objects. During the familiarization trial of NOR test, all mice (P17 to P18) spent equal time investigating the two objects in the arena.

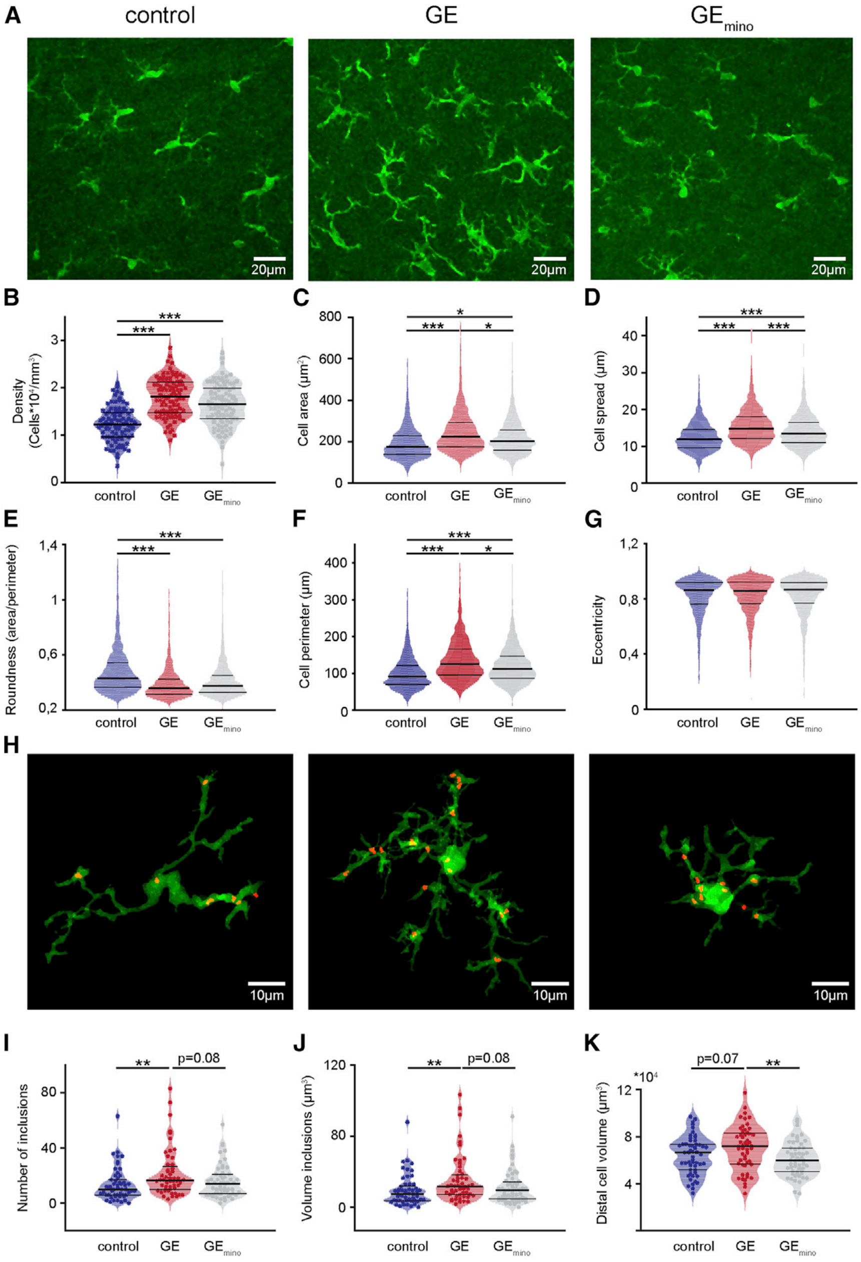

Figure 7. Altered Microglial Cell Morphology and Phagocytic Activity in Dual-Hit GE Mice Are Partially Restored by Minocycline Treatment

(A) Photographs of Iba-1-stained microglial cells in the PL of a P10 control mouse (left), of a P10 GE mouse (center), and of a P10 GE_{mino} mouse (right).

(B) Violin plot displaying the average density of Iba-1-stained cells in the PL of control (blue; n = 64 images from 4 pups), GE (red; n = 64 images from 4 pups), and GE_{mino} mice (gray; n = 64 images from 4 pups).

(C–G) Same as (B) for cell area (C), cell spread (D), roundness (E), perimeter (F), and eccentricity (G). For (C)–(G), n = 1,250, 1,738, and 1,614 cells, respectively, from 12 sections of 4 pups for all three conditions.

(H) Photographs of Iba-1-stained microglial cells and phagocyted VGLUT-1 puncta in the PL of a P8 control mouse (left), of a P8 GE mouse (center), and of a P8 GE_{mino} mouse (right).

(I) Violin plot displaying the number of inclusions per microglia cell in the PL of control (blue; n = 53 cells from 4 pups), GE (red; n = 52 cells from 4 pups), and GE_{mino} mice (gray; n = 55 cells from 4 pups).

(J and K) Same as (I) for the volume of inclusions per microglia cell (J) and the distal volume of microglia cells (K). Data are presented as median with 25th and 75th percentile.

In (B) and (I)–(K), single data points are displayed as asterisks, whereas in (C)–(G), single data points are omitted due to their high number. *$p < 0.05$, **$p < 0.01$, and ***$p < 0.001$; linear mixed-effect model with animal as a random effect (B–G) and robust, bootstrapped ANOVA with 20% level of trimming for the mean (I–K).

During the testing phase, GE mice (n = 15) showed poorer recognition abilities as mirrored by the lower discrimination ratio index and single interaction time when compared with control (n = 15) and GE_{mino} mice (n = 16; Figure 8B). Despite this trend, the differences did not reach significance levels, most likely due to high inter-animal variability reported for NOR test. During RR task, mice (P19–P22) had to process temporal information by recognizing the object with which they most recently interacted. GE mice had a significantly poorer discrimination ratio (condition effect p = 0.013) when compared to both control ($p < 10^{-4}$) and GE_{mino} mice (p = 0.038; Figure 8D). The exploratory and anxiety behavior was similar for all three groups of mice, indicating that the poor performance of GE mice does not result from lower motor abilities or fear to approach the objects. Taking into account the similarity of behavioral performance in control and GE_{mino} mice, we suggest that the recognition abilities of pre-juvenile GE mice are rescued after transient treatment with minocycline during early postnatal development. Importantly, the timing of minocycline administration is crucial for the rescue. When we administered minocycline from P9 to P16 to GE mice (GE_{mino}late; n = 16), the RR deficits persisted, the discrimination ratio being significantly decreased when compared to controls (n = 14; p = 0.03; Figure S9A). Moreover, when we considered the entire behavioral dataset, we found that GE_{mino}late mice have a RR deficit even when compared to GE_{mino} mice (p = 0.037) and are not significantly different from untreated GE mice (p = 0.85).

To confirm the link between prefrontal dysfunction throughout development and behavioral performance at pre-juvenile age, we resorted to a machine-learning classification algorithm with different inputs (discrimination ratios and single interaction time for the two tasks) but a similar architecture (k-nearest neighbors classification) to the one discussed above (Figure 7E). As

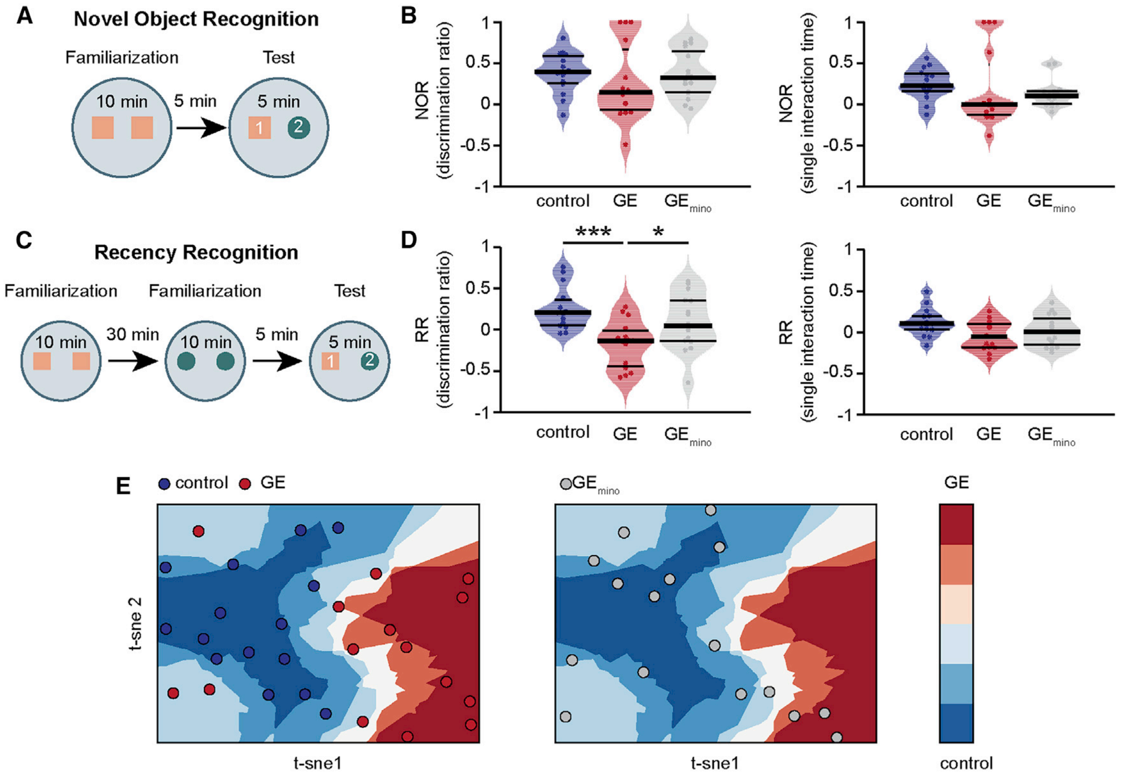

Figure 8. Minocycline Treatment Rescues Behavioral Deficits in Dual-Hit GE Mice

(A) Schematic diagram of the experimental protocol for NOR.

(B) Violin plot displaying NOR discrimination ratio (middle) and single interaction time (right) of control (blue; n = 15), GE (red; n = 15), and GE_{mino} (gray; n = 16) mice.

(C and D) Same as (A) and (B) for RR.

(E) T-SNE plot of behavioral features of control (blue dots) and GE (red dots) mice in the training/cross-validation set (left) and GE_{mino} (gray dots; right).

In (B) and (D), data are presented as median with 25th and 75th percentile, and single data points are shown as asterisks. *$p < 0.05$ and ***$p < 0.001$; robust, bootstrapped ANOVA with 20% level of trimming for the mean (B and D).

before, we iteratively (n = 500) used 3-fold cross-validation (n = 20 mice in the training set and n = 10 in the cross-validation set) to tune the algorithm hyper-parameters (training set) and assess its accuracy (cross-validation set). By these means, we were able to obtain a median classification accuracy of 83% on the cross-validation set. When we asked the algorithm to predict to which class GE_{mino} mice belonged to, 75% of them were classified as belonging to the control group (Figure 8E). These data show that, on a group level, early disruption of prefrontal networks is predictive of later impaired cognition. Accordingly, early rescue of such deficits is associated with restored cognitive development.

DISCUSSION

Although neurodevelopmental miswiring has been postulated to result in major functional and behavioral deficits at adulthood, the mechanisms of early impairment are still largely unresolved. A major consequence of this knowledge gap is the poor understanding of disease pathophysiology that hampers the development of tailored therapies for mental illness. Toward the aim of elucidating the substrate of developmental dysfunction, the present study uncovers layer- and cell-type-specific deficits in the PL of neonatal mice reproducing the gene-environment interactions involved in the pathogenesis of psychiatric disorders. We show that, in dual-hit GE mice, (1) the lower entrainment of neonatal prefrontal circuits in beta-gamma oscillations relates to structural and functional deficits of superficial layers pyramidal neurons; (2) minocycline administration during the first postnatal week restores the morphology, synaptic function, and firing and oscillatory patterns in local prefrontal circuits; and (3) at group level, early prefrontal network activity is predictive of pre-juvenile cognitive abilities. These findings highlight the major contribution of glutamatergic dysfunction to the abnormal refinement of circuits during development and support the hypothesis that such deficits emerge already at neonatal age. Moreover, the results demonstrate the efficacy of minocycline in preventing the emergence of developmental circuit dysfunction with relevance for cognitive disabilities. A limitation of the current study is that major conclusions rely on investigations at group level. Future longitudinal recordings across entire postnatal development, which are still currently technically demanding, will prove whether these conclusions hold also on an individual level.

Wiring of Prefrontal Circuits at Neonatal Age: Checkpoint of Cognitive Maturation

Anatomical investigations revealed that, although the PFC develops according to a similar time schedule as other neocortical areas, some maturation events (e.g., volumetric decline and growth and pruning of afferents and efferents) are protracted (van Eden et al., 1990). Correspondingly, the prefrontal patterns of coordinated activity share the general spatial and temporal organization of early neocortical oscillations (Hanganu-Opatz, 2010), yet they emerge later and have a frequency-specific structure. In rodents, the discontinuous oscillatory activity of PFC appears 1 to 2 days later than in the hippocampus and 2 to 3 days later than in primary visual and somatosensory cortices (Brockmann et al., 2011). The neonatal oscillations are detectable and have similar organization in both urethane-anesthetized and non-anesthetized rats and mice, yet their magnitude decreased under anesthesia, as shown by the present and previous studies (Chini et al., 2019).

The present findings show that frequency-specific communication within local prefrontal circuits emerges very early. Such precise interactions might represent the pre-requisite for functional entrainment of adult networks and cognitive performance. Abundant literature highlighted the link between theta-band hippocampal activation and fast oscillatory entrainment of prefrontal circuits during various cognitive tasks at adulthood (Sirota et al., 2008). Currently, only few attempts have been made to directly prove the role of timed interactions during development for the later emergence of network function and adult behavior. Recently, the key role of vasoactive intestinal peptide (VIP) and cholecystokinine (CCK)-positive interneurons for cortical circuit development has been demonstrated (Batista-Brito et al., 2017; Del Pino et al., 2017). In the same line, our findings identify pyramidal neurons in superficial layers as generators of early activity, facilitating the coupling within local neocortical circuits and glutamatergic communication between upper and deeper layers (Anastasiades and Butt, 2012), whose early function later impacts cognitive abilities. Identification of key cellular elements controlling circuit development opens new perspectives for the interrogation of long-term network effects.

Mechanisms of Abnormal Wiring in Prefrontal Circuits of Neonatal Dual-Hit GE Mice

The pathogenesis of cognitive dysfunction in major psychiatric disorders has been reported to involve interactions between a large number of susceptibility genes and environmental factors that might act at diverse stages of development (van Os et al., 2008). In the present study, we combine the abnormally translocated DISC1 gene with viral infection causing maternal immune activation (Meyer et al., 2005). This dual hit has a clear link to human pathology (Ayhan et al., 2009). Although each of the factors (i.e., either genetic or environmental) leads to structural, functional, and cognitive deficits of weak to moderate magnitude, it is only their combination that has been found to produce a neurobehavioral phenotype at adulthood that resembles aspects of mental illness. For example, the prefrontal-hippocampal networks accounting for mnemonic and executive abilities at adult age show major developmental deficits when both hits co-occur (abnormal DISC1 and maternal immune activation), whereas single-hit models show a largely normal network development (Hartung et al., 2016). In dual-hit GE mice, the patterns of coordinated activity in PFC and hippocampus appeared disorganized, and the long-range coupling between them was weaker at neonatal age.

Although these data demonstrate the developmental origin of dysfunction in dual-hit GE mice, they do not mechanistically explain the network and behavioral deficits. These deficits might result from either abnormal maturation of local prefrontal networks, a weaker theta activity in hippocampus, or sparser connectivity between the two areas. The present results fill the knowledge gap and identify the pyramidal neurons of superficial layers as key players of developmental miswiring, whereas pyramidal neurons in deeper cortical layers are indistinguishable in their structure and function in controls and dual-hit GE mice. The disorganized patterns of oscillatory activity in the PL result from superficial layers neurons that lost their timed firing and cannot generate the entrainment of local circuits in beta-low gamma frequencies. In turn, the neuronal spiking is controlled by the inputs that these neurons receive. Taking into account the oversimplified dendritic arborization and reduced number of spines, it is likely that pyramidal neurons in superficial layers of PL from dual-hit GE mice receive fewer inputs, which are randomly timed. The cell-type- and layer-dependent structural abnormalities in PFC (e.g., decreased dendritic spine density) have also been detected in other mouse models of schizophrenia (Koukouli et al., 2017) and in schizophrenia patients, in which it has been related to abnormalities in the excitatory transmission (Kolluri et al., 2005). Despite the integrity of pyramidal neurons in deep layers both at morphological and functional level, the local prefrontal circuitry relying on dense vertical and horizontal interactions between upper and deeper layers is compromised in dual-hit GE mice. Therefore, the theta hippocampal drive targeting deep layers pyramidal neurons cannot optimally entrain the PFC. Although some properties of oscillatory activity and neuronal firing over prefrontal layers are similar in P8–P10 GE mice and P4–P6 control mice, the overall properties of activity patterns suggest that the neonatal dysfunction is not solely the result of a delayed maturation caused by abnormal DISC1 and environmental stressors.

The selective structural deficits and dysfunction of pyramidal neurons in prefrontal superficial layers was prevented by minocycline administration during the first, but not the second, postnatal week. Minocycline is a pleiotropic drug that, among having other functions, is a potent inhibitor of microglial activation (Dean et al., 2012). Altered number of activated microglia has been found in the brain of MIA offspring (Borrell et al., 2002). Resulting from maternal infection, the stimulation of cytokine pathways (Meyer et al., 2005) and microglia overpruning of synapses (Neniskyte and Gross, 2017) have been proposed to interfere with developmental processes, such as neuronal proliferation, differentiation, and synaptogenesis (Neniskyte and Gross, 2017). A similar mechanism, microglia excessively engulfing the synaptic terminals, might represent the mechanism underlying the deficits reported here for dual-hit GE mice. During the first postnatal week, these MIA-induced

deficits alone seem to have no functional readout, because neonatal one-hit environmental mice (i.e., MIA offspring) have largely normal firing and network activity patterns (Hartung et al., 2016). Solely the combination with genetic risk factors, such as mutant DISC1, causes early circuit miswiring, as reported in the present study. DISC1 has a key role in neuronal proliferation and migration as well as in development and maintenance of synapses. However, the phenotypes in the mouse models of mutated DISC1 are rather modest (Brandon and Sawa, 2011). They become potentiated by the synergistic combination with MIA. This could be due to the fact that mutated DISC1 might modulate the basal or polyI:C-induced cytokine production by interfering with glycogen synthase kinase-3 (Beurel et al., 2010). Alternatively, DISC1 might confer neuronal vulnerability, making pyramidal neurons more susceptible to environmental stressors.

Minocycline has been found to be neuroprotective in numerous pathologies (Hinwood et al., 2013). In particular, its use alone or as adjunctive therapy to antipsychotics improved the behavioral and cognitive performance of schizophrenia patients (De Picker et al., 2017; Miyaoka et al., 2008). Although the mode of action of minocycline in the adult brain has been well characterized, only few studies focused on its preventive potential during development, before the onset of disease symptoms. Recently, minocycline use during adolescence was associated with a reduction in the incidence of psychosis, most likely by reducing microglia-mediated synapse uptake (Sellgren et al., 2019). In mice, when administrated during the course of peripubertal stress exposure, minocycline has been found to prevent the emergence of multiple behavioral abnormalities relevant to human cognitive dysfunction (Giovanoli et al., 2016), yet the mechanisms underlying the behavioral rescue are largely unknown. Here, we show that, already during neonatal development, minocycline is effective in preventing prelimbic structural, functional, and behavioral deficits. One possible mechanism of these effects is its action on microglia. However, the pathways that selectively link pyramidal neurons in superficial layers of PFC with the anti-inflammatory action of minocycline remain to be investigated in detail and in a more mechanistic manner. Of note, superficial layers neurons are thought of being more dependent on microglia activity than those of deep layers (Neniskyte and Gross, 2017). This might contribute to the layer-specific differences that we identified in the present study.

Because the efficacy of minocycline fades if it is administered at a later point of development, the question arises why the PFC during the investigated time window (i.e., P8–P10) is particularly sensitive to perturbations. Future studies need to address the role of hippocampal projections that drive the initial beta-gamma entrainment of prefrontal circuits (Brockmann et al., 2011; Ahlbeck et al., 2018) as well as of neuromodulators, such as dopamine with D1 receptors first appearing at this age (Leslie et al., 1991).

Relevance for Human Mental Illness

The relevance of animal models for human mental disorders has often been questioned, because they do not fulfill the validity criteria used for other pathologies. Optimally, animal models recapitulate etiologic processes (i.e., construct validity) or symptom features (i.e., face validity). In case of mental disorders, such as schizophrenia, bipolar disorder, or depression, the available mouse models have either excellent construct validity (e.g., models mimicking the genetic background) but limited face validity or vice versa (e.g., models of hippocampal damage or pharmacological models). Dual-hit models mimic both genetic and environmental risk factors and recapitulate some of the structural and circuit deficits observed in patients. For example, lower spine density in upper layers of PFC as well as dysfunctional prefrontal gamma-band oscillations, which have been reported here, have also been described for schizophrenia patients (Senkowski and Gallinat, 2015). Similarly, microglia abnormalities and resulting synaptic deficits have been related to several brain pathologies (Neniskyte and Gross, 2017). Therefore, we propose that dual-hit GE mice recapitulate both the etiology (construct validity) as well as the general rules of neuronal, glial, and circuit dysfunction (face validity) that relate to cognitive impairment in mental disorders. They appear highly instrumental for the identification of cellular key players of disease that, for ethical and technical reasons, are not accessible in humans of comparable age. This brings us closer to one of the major goals of circuit psychiatry that is the identification of key neurobiological targets amenable to tailored therapies (Gordon, 2016) that not only treat but also prevent disease-related cognitive and behavioral deficits.

STAR★METHODS

Detailed methods are provided in the online version of this paper and include the following:

- KEY RESOURCES TABLE
- LEAD CONTACT AND MATERIALS AVAILABILITY
- EXPERIMENTAL MODEL AND SUBJECT DETAILS
  - Mice
  - Minocycline administration
- METHOD DETAILS
  - In utero electroporation
  - Developmental milestones
  - Behavioral experiments
  - In vitro electrophysiology and optogenetics
  - In vivo electrophysiology and optogenetics
  - Histology
- QUANTIFICATION AND STATISTICAL ANALYSIS
  - Image analysis
  - In vitro electrophysiology
  - In vivo electrophysiology
  - K-nearest neighbors classifiers
  - Statistical analysis
- DATA AND CODE AVAILABILITY

SUPPLEMENTAL INFORMATION

Supplemental Information can be found online at https://doi.org/10.1016/j.neuron.2019.09.042.

ACKNOWLEDGMENTS

We thank Dr. Joseph Gogos for providing the DISC1 mice, Drs. S. Wiegert and T. Oertner for providing opsin derivates, and A. Marquardt, A. Dahlmann, I. Ohmert, and K. Titze for excellent technical assistance. This work was funded by grants from the European Research Council (ERC-2015-CoG 681577 to I.L.H.-O.) and the German Research Foundation (Ha4466/10-1, Ha4466/11-1, Ha4466/12-1, SPP 1665, and SFB 936 B5 to I.L.H.-O. and C6 to C.M.). I.L.H.-O. is a founding member of the FENS Kavli Network of Excellence.

AUTHOR CONTRIBUTIONS

I.L.H.-O. conceived the study. M.C. and I.L.H.-O. designed the experiments. M.C., J.A.P., C.L., L.C.-P., M.H., V.O., J.A., and S.H.B. carried out experiments. M.C. and X.X. analyzed the data. M.C., C.M., and I.L.H.-O. interpreted the data. M.C. and I.L.H.-O. wrote the article. All authors discussed and commented on the manuscript.

DECLARATION OF INTERESTS

The authors declare no competing interests.

Received: March 28, 2019
Revised: August 14, 2019
Accepted: September 24, 2019
Published: November 13, 2019

REFERENCES

Aarts, E., Verhage, M., Veenvliet, J.V., Dolan, C.V., and van der Sluis, S. (2014). A solution to dependency: using multilevel analysis to accommodate nested data. Nat. Neurosci. *17*, 491–496.

Abazyan, B., Nomura, J., Kannan, G., Ishizuka, K., Tamashiro, K.L., Nucifora, F., Pogorelov, V., Ladenheim, B., Yang, C., Krasnova, I.N., et al. (2010). Prenatal interaction of mutant DISC1 and immune activation produces adult psychopathology. Biol. Psychiatry *68*, 1172–1181.

Ahlbeck, J., Song, L., Chini, M., Bitzenhofer, S.H., and Hanganu-Opatz, I.L. (2018). Glutamatergic drive along the septo-temporal axis of hippocampus boosts prelimbic oscillations in the neonatal mouse. eLife *7*, e33158.

Anastasiades, P.G., and Butt, S.J. (2012). A role for silent synapses in the development of the pathway from layer 2/3 to 5 pyramidal cells in the neocortex. J. Neurosci. *32*, 13085–13099.

Ayhan, Y., Sawa, A., Ross, C.A., and Pletnikov, M.V. (2009). Animal models of gene-environment interactions in schizophrenia. Behav. Brain Res. *204*, 274–281.

Batista-Brito, R., Vinck, M., Ferguson, K.A., Chang, J.T., Laubender, D., Lur, G., Mossner, J.M., Hernandez, V.G., Ramakrishnan, C., Deisseroth, K., et al. (2017). Developmental dysfunction of VIP interneurons impairs cortical circuits. Neuron *95*, 884–895.e9.

Bellesi, M., de Vivo, L., Chini, M., Gilli, F., Tononi, G., and Cirelli, C. (2017). Sleep loss promotes astrocytic phagocytosis and microglial activation in mouse cerebral cortex. J. Neurosci. *37*, 5263–5273.

Beurel, E., Michalek, S.M., and Jope, R.S. (2010). Innate and adaptive immune responses regulated by glycogen synthase kinase-3 (GSK3). Trends Immunol. *31*, 24–31.

Bitzenhofer, S.H., Sieben, K., Siebert, K.D., Spehr, M., and Hanganu-Opatz, I.L. (2015). Oscillatory activity in developing prefrontal networks results from theta-gamma-modulated synaptic inputs. Cell Rep. *11*, 486–497.

Bitzenhofer, S.H., Ahlbeck, J., Wolff, A., Wiegert, J.S., Gee, C.E., Oertner, T.G., and Hanganu-Opatz, I.L. (2017). Layer-specific optogenetic activation of pyramidal neurons causes beta-gamma entrainment of neonatal networks. Nat. Commun. *8*, 14563.

Borrell, J., Vela, J.M., Arévalo-Martin, A., Molina-Holgado, E., and Guaza, C. (2002). Prenatal immune challenge disrupts sensorimotor gating in adult rats. Implications for the etiopathogenesis of schizophrenia. Neuropsychopharmacology *26*, 204–215.

Bosman, C.A., Lansink, C.S., and Pennartz, C.M. (2014). Functions of gamma-band synchronization in cognition: from single circuits to functional diversity across cortical and subcortical systems. Eur. J. Neurosci. *39*, 1982–1999.

Brandon, N.J., and Sawa, A. (2011). Linking neurodevelopmental and synaptic theories of mental illness through DISC1. Nat. Rev. Neurosci. *12*, 707–722.

Brockmann, M.D., Pöschel, B., Cichon, N., and Hanganu-Opatz, I.L. (2011). Coupled oscillations mediate directed interactions between prefrontal cortex and hippocampus of the neonatal rat. Neuron *71*, 332–347.

Chini, M., Gretenkord, S., Kostka, J.K., Pöpplau, J.A., Cornelissen, L., Berde, C.B., Hanganu-Opatz, I.L., and Bitzenhofer, S.H. (2019). Neural correlates of anesthesia in newborn mice and humans. Front. Neural Circuits *13*, 38.

Cho, K.K., Hoch, R., Lee, A.T., Patel, T., Rubenstein, J.L., and Sohal, V.S. (2015). Gamma rhythms link prefrontal interneuron dysfunction with cognitive inflexibility in Dlx5/6(+/-) mice. Neuron *85*, 1332–1343.

Cichon, N.B., Denker, M., Grün, S., and Hanganu-Opatz, I.L. (2014). Unsupervised classification of neocortical activity patterns in neonatal and pre-juvenile rodents. Front. Neural Circuits *8*, 50.

Cirelli, C., and Tononi, G. (2015). Cortical development, electroencephalogram rhythms, and the sleep/wake cycle. Biol. Psychiatry *77*, 1071–1078.

Clement, E.A., Richard, A., Thwaites, M., Ailon, J., Peters, S., and Dickson, C.T. (2008). Cyclic and sleep-like spontaneous alternations of brain state under urethane anaesthesia. PLoS ONE *3*, e2004.

Dansie, L.E., Phommahaxay, K., Okusanya, A.G., Uwadia, J., Huang, M., Rotschafer, S.E., Razak, K.A., Ethell, D.W., and Ethell, I.M. (2013). Long-lasting effects of minocycline on behavior in young but not adult Fragile X mice. Neuroscience *246*, 186–198.

De Picker, L.J., Morrens, M., Chance, S.A., and Boche, D. (2017). Microglia and brain plasticity in acute psychosis and schizophrenia illness course: a meta-review. Front. Psychiatry *8*, 238.

Deakin, B., Suckling, J., Barnes, T.R.E., Byrne, K., Chaudhry, I.B., Dazzan, P., Drake, R.J., Giordano, A., Husain, N., Jones, P.B., et al.; BeneMin Study team (2018). The benefit of minocycline on negative symptoms of schizophrenia in patients with recent-onset psychosis (BeneMin): a randomised, double-blind, placebo-controlled trial. Lancet Psychiatry *5*, 885–894.

Dean, O.M., Data-Franco, J., Giorlando, F., and Berk, M. (2012). Minocycline: therapeutic potential in psychiatry. CNS Drugs *26*, 391–401.

Del Pino, I., Brotons-Mas, J.R., Marques-Smith, A., Marighetto, A., Frick, A., Marín, O., and Rico, B. (2017). Abnormal wiring of CCK$^+$ basket cells disrupts spatial information coding. Nat. Neurosci. *20*, 784–792.

Emadi-Kouchak, H., Mohammadinejad, P., Asadollahi-Amin, A., Rasoulinejad, M., Zeinoddini, A., Yalda, A., and Akhondzadeh, S. (2016). Therapeutic effects of minocycline on mild-to-moderate depression in HIV patients: a double-blind, placebo-controlled, randomized trial. Int. Clin. Psychopharmacol. *31*, 20–26.

Garrido-Mesa, N., Zarzuelo, A., and Gálvez, J. (2013). Minocycline: far beyond an antibiotic. Br. J. Pharmacol. *169*, 337–352.

Giovanoli, S., Weber-Stadlbauer, U., Schedlowski, M., Meyer, U., and Engler, H. (2016). Prenatal immune activation causes hippocampal synaptic deficits in the absence of overt microglia anomalies. Brain Behav. Immun. *55*, 25–38.

Gordon, J.A. (2016). On being a circuit psychiatrist. Nat. Neurosci. *19*, 1385–1386.

Hanganu-Opatz, I.L. (2010). Between molecules and experience: role of early patterns of coordinated activity for the development of cortical maps and sensory abilities. Brain Res. Brain Res. Rev. *64*, 160–176.

Harris, K.D., and Shepherd, G.M. (2015). The neocortical circuit: themes and variations. Nat. Neurosci. *18*, 170–181.

Hartung, H., Cichon, N., De Feo, V., Riemann, S., Schildt, S., Lindemann, C., Mulert, C., Gogos, J.A., and Hanganu-Opatz, I.L. (2016). From shortage to surge: a developmental switch in hippocampal-prefrontal coupling in a

gene-environment model of neuropsychiatric disorders. Cereb. Cortex *26*, 4265–4281.

Hinwood, M., Tynan, R.J., Charnley, J.L., Beynon, S.B., Day, T.A., and Walker, F.R. (2013). Chronic stress induced remodeling of the prefrontal cortex: structural re-organization of microglia and the inhibitory effect of minocycline. Cereb. Cortex *23*, 1784–1797.

Ignacio, M.P.D., Kimm, E.J., Kageyama, G.H., Yu, J., and Robertson, R.T. (1995). Postnatal migration of neurons and formation of laminae in rat cerebral cortex. Anat. Embryol. (Berl.) *191*, 89–100.

Khazipov, R., Sirota, A., Leinekugel, X., Holmes, G.L., Ben-Ari, Y., and Buzsáki, G. (2004). Early motor activity drives spindle bursts in the developing somatosensory cortex. Nature *432*, 758–761.

Kirmse, K., Kummer, M., Kovalchuk, Y., Witte, O.W., Garaschuk, O., and Holthoff, K. (2015). GABA depolarizes immature neurons and inhibits network activity in the neonatal neocortex in vivo. Nat. Commun. *6*, 7750.

Kishimoto, T., Horigome, T., and Takamiya, A. (2018). Minocycline as a treatment for schizophrenia: is the discussion truly finished? Lancet Psychiatry *5*, 856–857.

Kobayashi, K., Imagama, S., Ohgomori, T., Hirano, K., Uchimura, K., Sakamoto, K., Hirakawa, A., Takeuchi, H., Suzumura, A., Ishiguro, N., and Kadomatsu, K. (2013). Minocycline selectively inhibits M1 polarization of microglia. Cell Death Dis. *4*, e525.

Kolluri, N., Sun, Z., Sampson, A.R., and Lewis, D.A. (2005). Lamina-specific reductions in dendritic spine density in the prefrontal cortex of subjects with schizophrenia. Am. J. Psychiatry *162*, 1200–1202.

Koukouli, F., Rooy, M., Tziotis, D., Sailor, K.A., O'Neill, H.C., Levenga, J., Witte, M., Nilges, M., Changeux, J.P., Hoeffer, C.A., et al. (2017). Nicotine reverses hypofrontality in animal models of addiction and schizophrenia. Nat. Med. *23*, 347–354.

Leicht, G., Vauth, S., Polomac, N., Andreou, C., Rauh, J., Mußmann, M., Karow, A., and Mulert, C. (2016). EEG-informed fMRI reveals a disturbed gamma-band-specific network in subjects at high risk for psychosis. Schizophr. Bull. *42*, 239–249.

Leslie, C.A., Robertson, M.W., Cutler, A.J., and Bennett, J.P., Jr. (1991). Postnatal development of D1 dopamine receptors in the medial prefrontal cortex, striatum and nucleus accumbens of normal and neonatal 6-hydroxydopamine treated rats: a quantitative autoradiographic analysis. Brain Res. Dev. Brain Res. *62*, 109–114.

Luzi, P., Abraham, R.M., Rafi, M.A., Curtis, M., Hooper, D.C., and Wenger, D.A. (2009). Effects of treatments on inflammatory and apoptotic markers in the CNS of mice with globoid cell leukodystrophy. Brain Res. *1300*, 146–158.

Marín, O. (2016). Developmental timing and critical windows for the treatment of psychiatric disorders. Nat. Med. *22*, 1229–1238.

Marques-Smith, A., Lyngholm, D., Kaufmann, A.K., Stacey, J.A., Hoerder-Suabedissen, A., Becker, E.B., Wilson, M.C., Molnár, Z., and Butt, S.J. (2016). A transient translaminar GABAergic interneuron circuit connects thalamocortical recipient layers in neonatal somatosensory cortex. Neuron *89*, 536–549.

Meyer, U., Feldon, J., Schedlowski, M., and Yee, B.K. (2005). Towards an immuno-precipitated neurodevelopmental animal model of schizophrenia. Neurosci. Biobehav. Rev. *29*, 913–947.

Miyamoto, A., Wake, H., Ishikawa, A.W., Eto, K., Shibata, K., Murakoshi, H., Koizumi, S., Moorhouse, A.J., Yoshimura, Y., and Nabekura, J. (2016). Microglia contact induces synapse formation in developing somatosensory cortex. Nat. Commun. *7*, 12540.

Miyaoka, T., Yasukawa, R., Yasuda, H., Hayashida, M., Inagaki, T., and Horiguchi, J. (2008). Minocycline as adjunctive therapy for schizophrenia: an open-label study. Clin. Neuropharmacol. *31*, 287–292.

Neniskyte, U., and Gross, C.T. (2017). Errant gardeners: glial-cell-dependent synaptic pruning and neurodevelopmental disorders. Nat. Rev. Neurosci. *18*, 658–670.

Rossant, C., Kadir, S.N., Goodman, D.F.M., Schulman, J., Hunter, M.L.D., Saleem, A.B., Grosmark, A., Belluscio, M., Denfield, G.H., Ecker, A.S., et al. (2016). Spike sorting for large, dense electrode arrays. Nat. Neurosci. *19*, 634–641.

Savitz, J.B., Teague, T.K., Misaki, M., Macaluso, M., Wurfel, B.E., Meyer, M., Drevets, D., Yates, W., Gleason, O., Drevets, W.C., and Preskorn, S.H. (2018). Treatment of bipolar depression with minocycline and/or aspirin: an adaptive, 2×2 double-blind, randomized, placebo-controlled, phase IIA clinical trial. Transl. Psychiatry *8*, 27.

Schafer, D.P., Lehrman, E.K., Kautzman, A.G., Koyama, R., Mardinly, A.R., Yamasaki, R., Ransohoff, R.M., Greenberg, M.E., Barres, B.A., and Stevens, B. (2012). Microglia sculpt postnatal neural circuits in an activity and complement-dependent manner. Neuron *74*, 691–705.

Selemon, L.D., and Zecevic, N. (2015). Schizophrenia: a tale of two critical periods for prefrontal cortical development. Transl. Psychiatry *5*, e623.

Sellgren, C.M., Gracias, J., Watmuff, B., Biag, J.D., Thanos, J.M., Whittredge, P.B., Fu, T., Worringer, K., Brown, H.E., Wang, J., et al. (2019). Increased synapse elimination by microglia in schizophrenia patient-derived models of synaptic pruning. Nat. Neurosci. *22*, 374–385.

Senkowski, D., and Gallinat, J. (2015). Dysfunctional prefrontal gamma-band oscillations reflect working memory and other cognitive deficits in schizophrenia. Biol. Psychiatry *77*, 1010–1019.

Shin Yim, Y., Park, A., Berrios, J., Lafourcade, M., Pascual, L.M., Soares, N., Yeon Kim, J., Kim, S., Kim, H., Waisman, A., et al. (2017). Reversing behavioural abnormalities in mice exposed to maternal inflammation. Nature *549*, 482–487.

Sirota, A., Montgomery, S., Fujisawa, S., Isomura, Y., Zugaro, M., and Buzsáki, G. (2008). Entrainment of neocortical neurons and gamma oscillations by the hippocampal theta rhythm. Neuron *60*, 683–697.

Uhlhaas, P.J., and Singer, W. (2015). Oscillations and neuronal dynamics in schizophrenia: the search for basic symptoms and translational opportunities. Biol. Psychiatry *77*, 1001–1009.

van Eden, C.G., Kros, J.M., and Uylings, H.B. (1990). The development of the rat prefrontal cortex. Its size and development of connections with thalamus, spinal cord and other cortical areas. Prog. Brain Res. *85*, 169–183.

van Os, J., Rutten, B.P., and Poulton, R. (2008). Gene-environment interactions in schizophrenia: review of epidemiological findings and future directions. Schizophr. Bull. *34*, 1066–1082.

Vinck, M., van Wingerden, M., Womelsdorf, T., Fries, P., and Pennartz, C.M. (2010). The pairwise phase consistency: a bias-free measure of rhythmic neuronal synchronization. Neuroimage *51*, 112–122.

Weinhard, L., di Bartolomei, G., Bolasco, G., Machado, P., Schieber, N.L., Neniskyte, U., Exiga, M., Vadisiute, A., Raggioli, A., Schertel, A., et al. (2018). Microglia remodel synapses by presynaptic trogocytosis and spine head filopodia induction. Nat. Commun. *9*, 1228.

Zhang, L., Zheng, H., Wu, R., Zhu, F., Kosten, T.R., Zhang, X.Y., and Zhao, J. (2018). Minocycline adjunctive treatment to risperidone for negative symptoms in schizophrenia: association with pro-inflammatory cytokine levels. Prog. Neuropsychopharmacol. Biol. Psychiatry *85*, 69–76.

STAR★METHODS

KEY RESOURCES TABLE

REAGENT or RESOURCE	SOURCE	IDENTIFIER
Antibodies		
mouse monoclonal Alexa Fluor-488 conjugated antibody against NeuN	Merck Millipore	Cat# MAB377X; RRID: AB_2149209
rabbit polyclonal primary antibody against GABA	Sigma-Aldrich	Cat#A2052; RRID: AB_477652
Alexa Fluor-488 goat anti-rabbit IgG secondary antibody	Merck Millipore	Cat# A-11008; RRID: AB_143165
rabbit monoclonal primary antibody against IBA-1	Wako	Cat# 019-19741; RRID: AB_839504
Deposited Data		
LFP and SUA data for all the non-anesthetized mice	This paper	https://gin.g-node.org/mchini/Resolving_and_rescuing_developmental_miswiring_in_a_mouse_model_of_cognitive_impairment
Chemicals, Peptides, and Recombinant Proteins		
Isoflurane	Abbott	B506
Urethane	Fluka analytical	94300
Minocycline	Sigma-Aldrich	M9511
Experimental Models: Organisms/Strains		
Mouse: C57BL/6J	Universitätsklinikum Hamburg-Eppendorf – Animal facility	N/A
Mouse: Disc1Tm1Kara /C57BL/6J	J. Gogos Lab	N/A
Recombinant DNA		
pAAV-CAG-ChR2(E123T/T159C)-2AtDimer2	T. G. Oertner Lab	http://www.oertner.com/
pAAV-CAG-tDimer2	T. G. Oertner Lab	http://www.oertner.com/
Software and Algorithms		
MATLAB R2016a	MathWorks	https://www.mathworks.com
Offline Sorter	Plexon	http://plexon.com/
ImageJ 2.0.0	ImageJ	https://imagej.nih.gov/ij/
R Statistical Software 3.5.1	RStudio	https://rstudio.com
Cheetah 6	Neuralynx	https://neuralynx.com/
Anaconda 1.9.6	Anaconda	https://www.anaconda.com
Spyder 3.3.2	Spyder	https://www.spyder-ide.org
Video Mot2	TSE Systems	https://www.tse-systems.com/product-details/videomot
Other		
Arduino Uno SMD	Arduino	A000073
Digital Lynx 4SX	Neuralynx	https://neuralynx.com/
Diode laser (473 nm)	Omicron	LuxX 473-100
Electroporation device	BEX	CUY21EX
Electroporation tweezer-type paddles	Protech	CUY650-P5
Recording electrode (1 shank, 16 channels)	Neuronexus	A1x16-3mm-703-A16
Recording optrode (1 shank, 16 channels)	Neuronexus	A1x16-5mm-703-OA16LP
Recording electrode (4 shanks, 16 channels)	Neuronexus	A4x4-3mm-100-125-703
Recording tetrode (4 shanks, 16 channels)	Neuronexus	A4x4-3mm-100-125-703-OA16LP

LEAD CONTACT AND MATERIALS AVAILABILITY

Further information and requests for resources and reagents should be directed to and will be fulfilled by the Lead Contact,Ileana L. Hanganu-Opatz (hangop@zmnh.uni-hamburg.de).

This study did not generate new unique reagents.

EXPERIMENTAL MODEL AND SUBJECT DETAILS

Mice

Experiments were performed in compliance with German laws and the guidelines of the European Community for the use of animals in research, and were approved by the local ethical committee (111/12, 132/12). Experiments were carried out on C57BL/6J mice of both sexes, at the age of P8–10. Heterozygous mutant DISC1 pups carrying a Disc1 allele (Disc1Tm1Kara) on a C57Bl6/J background and C57Bl6/J, whose dams were injected i.v. at embryonic day (E) 9 with the viral mimetic poly I:C (5 mg/kg) were used as dual-hit genetic-environmental model (dual-hit GE) (Hartung et al., 2016). Pups born from homozygous Disc1Tm1Kara dams and wild-type males and pups born from wild-type dams and homozygous Disc1Tm1Kara males were pooled together, as no difference between the two groups was found. Genotypes were assessed using genomic DNA (tail biopsies) and following primer sequences: forward primer 5′-TAGCCACTCTCATTGTCAGC-3′ and reverse primer 5′-CCTCATCCCTTCCACTCAGC-3′. Non-treated wild-type mice and the offspring of dams injected at E9 with saline (0.9%) were used as controls and combined together, as no difference between the two groups was found. For single-hit experiments, the offspring of wild-type E9 poly I:C injected dams (single-hit E) and heterozygous mutant Disc1Tm1Kara pups of E) saline injected dams (single-hit G) were used. Timed-pregnant mice from the animal facility of the University Medical Center Hamburg-Eppendorf, of both aforementioned conditions, were housed individually at a 12 h light/12 h dark cycle, and were given access to water and food *ad libitum*. The day of vaginal plug detection was considered as E0.5, while the day of birth as P0. In accordance with the three Rs guidelines of the European Community for the use of animals in research, we re-analyzed part of the mice used for one of our previous publications (Bitzenhofer et al., 2017).

Minocycline administration

Minocycline was administered to neonatal mice from either P1 to P8 or from P9 to P16 by adding it to the drinking water of the dam, which then passed it on to the offspring via lactation (Dansie et al., 2013). In line with previous studies (Dansie et al., 2013), the daily dosage of minocycline was 30 mg/kg body weight. To cover the taste of the antibiotic, sucrose was added to the solution. No difference in liquid intake was observed between dams receiving water and dams receiving water supplemented with minocycline. This administration route has been shown to result in detectable concentrations of the drug in the breast milk of the lactating dam (Luzi et al., 2009).

METHOD DETAILS

In utero electroporation

Additional wet food supplemented with 2-4 drops of Metacam (meloxicam; 0.5 mg ml−1; Boehringer-Ingelheim, Germany) was administered from one day before until two days after surgery. At E12.5 or E15.5 randomly assigned pregnant mice received a subcutaneous injection of buprenorphine (0.05 mg/kg body weight) at least 30 min before surgery. Surgery was performed on a heated surface; pain reflexes (toe and tail pinch) and breathing were monitored throughout. Under isoflurane anesthesia (induction: 5%; maintenance: 3.5%), the eyes of the dam were covered with eye ointment to prevent damage, before the uterine horns were exposed and moistened with warm sterile PBS (37 °C). Solution containing 1.25 μg/μl DNA (pAAV-CAG-ChR2(E123T/T159C)-2AtDimer2 or pAAV-CAG-tDimer2)) and 0.1% fast green dye at a volume of 0.75–1.25 μl were injected into the right lateral ventricle of individual embryos using pulled borosilicate glass capillaries with a sharp, long tip. Plasmid DNA was purified with NucleoBond (Macherey-Nagel, Germany). 2A encodes for a ribosomal skip sentence, splitting the fluorescent protein tDimer2 from the opsin during gene translation. Each embryo within the uterus was placed between the electroporation tweezer-type paddles (3 mm diameter for E12.5; 5 mm diameter for E14.5-15.5; Protech, TX, USA) that were roughly oriented at a 20° leftward angle from the midline and a 10° angle downward from anterior to posterior. By these means, neural precursor cells from the subventricular zone, which radially migrate into the medial PFC, were transfected. Electrode pulses (35 V, 50 ms) were applied five times at intervals of 950 ms controlled by an electroporator (CU21EX; BEX, Japan). Uterine horns were placed back into the abdominal cavity after electroporation, which was filled with warm sterile PBS (37 °C). Abdominal muscles and skin were sutured individually with absorbable and non-absorbable suture thread, respectively. After recovery, pregnant mice were returned to their home cages, which were half placed on a heating blanket for two days after surgery. Opsin expression was assessed with a portable fluorescent flashlight (Nightsea, MA, USA) through the intact skull and skin at P2–3 and confirmed post mortem by fluorescence microscopy in brain slices. Pups without expression in the PFC were excluded from the analysis.

Developmental milestones

Mouse pups were tested for their somatic development and reflexes at P2, P5 and P8. Weight, body and tail length were assessed. Surface righting reflex was quantified as time (max 30 s) until the pup turned over with all four feet on the ground after being placed on its back. Cliff aversion reflex was quantified as time (max 30 s) until the pup withdrew after snout and forepaws were positioned over an elevated edge. Vibrissa placing was rated positive if the pup turned its head after gently touching the whiskers with a toothpick.

Behavioral experiments

The behavioral experiments were carried out in pre-juvenile mice that did not experience IUE, using previously established experimental protocols. Briefly, all behavioral tests were conducted in a circular white arena, the size of which (D: 34 cm, H: 30 cm) maximized exploratory behavior, while minimizing incidental contact with testing objects. The objects used for testing of novelty recognition were five differently shaped, and colored, easy to clean items that were provided with magnets to fix them to the bottom of the arena. Object sizes (H: 3 cm, diameter: 1.5–3 cm) were smaller than twice the size of the mouse and did not resemble living stimuli (no eye spots, predator shape). The objects were positioned at 10 cm from the borders and 8 cm from the center of the arena. After every trial, the objects and arena were cleaned with 0.1% acetic acid to remove all odors. A black and white CCD camera (VIDEOR TECHNICAL E. Hartig GmbH) was mounted 100 cm above the arena and connected to a PC via PCI interface serving as frame grabber for video tracking software (Video Mot2 software, TSE Systems GmbH).

Exploratory Behavior in the Open Field

Pre-juvenile mice (P16-17) were allowed to freely explore the testing arena for 10 min. The floor area of the arena was digitally subdivided in 8 zones (4 center zones and 4 border zones) using the zone monitor mode of the VideoMot 2 analysis software (VideoMot 2, TSE Systems GmbH). The time spent by pups in center and border zones as well as the running distance and velocity were quantified. Mice that did not exit the center area for >1 min (n = 2) were excluded from further analysis.

Novelty Recognition Paradigms

All protocols for assessing item recognition memory in pre-juvenile mice consisted of familiarization and testing trials. During the familiarization trial, each mouse was placed into the arena containing two identical objects. The mice were released against the center of the opposite wall with the back to the objects. After 10 min of free exploration of objects, the mice were returned to a temporary holding cage. In the novel object recognition (NOR) task, tested in P17-P18 mice, the familiarization trial was followed 5 min later by a test trial in which one object used in the familiarization and one new object were placed in the arena at the same positions as during the familiarization trials. The mice were allowed to investigate the familiar and the novel object, with different shape and color, for 5 min. Object interaction during the first five minutes and the length of single interaction with the objects were analyzed and compared between the groups. In the recency recognition (RR) task, tested at P19–22, mice experienced two 10 min familiarization trials with two different sets of identical objects that were separated by a delay of 30 min. The second familiarization trial was followed after 5 min by a test trial in which one object used in the first and one object used in the second more recent familiarization trial were placed in the arena in the same positions as during the familiarization trials. Object interaction during the first five minutes and the length of single interaction with the objects were analyzed and compared between the groups. All trials were video-tracked using the Video Mot2 analysis software. The object recognition module of the software was used and a 3-point tracking method identified the head, the rear end and the center of gravity of the mouse. Digitally, a square zone was created around each object and every entry of the head point into this area was considered as object interaction. Climbing or sitting on the object, defined as having both head and center of gravity points within the square zone, were not counted as interactions. Data were imported and analyzed offline using custom-written tools in MATLAB software (MathWorks). Discrimination ratios were calculated as (Time spent interacting with novel object – time spent interacting with less recent object) / (Time spent interacting with novel object + time spent interacting with less recent object). Single interaction time ratios were analogously calculated.

In vitro electrophysiology and optogenetics

As previously described (Bitzenhofer et al., 2017), whole-cell patch-clamp recordings were performed from t-Dimer expressing superficial and deep layers prelimbic neurons in brain slices of P8–10 mice after IUE at E15.5 and E12.5, respectively. Briefly, pups were decapitated, brains were removed and immediately sectioned coronally at 300 μm in ice-cold oxygenated high sucrose-based artificial cerebral spinal fluid (ACSF) (in mM: 228 sucrose, 2.5 KCl, 1 NaH2PO4, 26.2 NaHCO3, 11 glucose, 7 MgSO4; 320 mOsm). Slices were incubated in oxygenated ACSF (in mM: 119 NaCl, 2.5 KCl, 1 NaH2PO4, 26.2 NaHCO3, 11 glucose, 1.3 MgSO4; 320 mOsm) at 37 °C for 45 min before cooling to room temperature and superfused with oxygenated ACSF in the recording chamber. tDimer2-positive neurons were patched under optical control using pulled borosilicate glass capillaries (tip resistance of 4-7 MΩ) filled with pipette solution (in mM: 130 K-gluconate, 10 HEPES, 0.5 EGTA, 4 Mg-ATP, 0.3 Na-GTP, 8 NaCl; 285 mOsm, pH 7.4). Recordings were controlled with the Ephus software in the MATLAB environment (MathWorks, MA, USA). Capacitance artifacts and series resistance were minimized using the built-in circuitry of the patch-clamp amplifier (Axopatch 200B; Molecular devices, CA, USA). Responses of neurons to hyper- and depolarizing current injections, as well as blue light pulses (473 nm, 5.2 mW/mm^2) were digitized at 5 kHz in current-clamp mode.

In vivo electrophysiology and optogenetics

Surgery

Multisite extracellular recordings were performed in the PL of P8–10 mice. For recordings in non-anesthetized state, 0.5% bupivacain / 1% lidocaine was locally applied on the neck muscles. For recordings in anesthetized state, mice were injected i.p. with urethane (1 mg/g body weight; Sigma-Aldrich) before surgery. For both groups, the surgery was performed under isoflurane anesthesia (induction: 5%; maintenance: 1.5%). The head of the pup was fixed into a stereotaxic apparatus using two plastic bars mounted on the nasal and occipital bones with dental cement. The bone above the PFC (0.5 mm anterior to bregma, 0.1 mm right to the midline for superficial layers, 0.5 mm for deep layers) was carefully removed by drilling a hole of < 0.5 mm in diameter. Before recordings, mice were allowed to recover for 10–20 min on a heating blanket.

One- or four-shank multisite optoelectrodes (NeuroNexus, MI, USA) were inserted 2.4 or 1.9 mm (respectively) deep into PFC, perpendicular to the skull surface. One-shank optoelectrodes contained 1 × 16 recordings sites (0.4-0.8 MΩ impedance, 100 μm spacing) aligned with an optical fiber (105 μm diameter) ending 200 μm above the top recording site. Four-shank optoelectrodes contained 4 × 4 recording sites (0.4-0.8 MΩ impedance, 100 μm spacing, 125 μm intershank spacing) aligned with optical fibers (50 μm diameter) ending 200 μm above the top recording sites. A silver wire was inserted into the cerebellum and served as ground and reference electrode. Before signal acquisition, a recovery period of 15 min after electrode insertion was provided.

Signal acquisition

Extracellular signals were band-pass filtered (0.1–9,000 Hz) and digitized (32 kHz) with a multichannel extracellular amplifier (Digital Lynx SX; Neuralynx, Bozeman, MO, USA) and the Cheetah acquisition software (Neuralynx). Spontaneous (i.e., not induced by light stimulation) activity was recorded for 15 min at the beginning of each recording session.

Light stimulation

Ramp (i.e., linearly increasing power) light stimulations were performed with an arduino uno (Arduino, Italy) controlled diode laser (473 nm; Omicron, Austria). Laser power was adjusted to trigger neuronal spiking in response to >25% of 3-ms-long light pulses at 16 Hz. Resulting light power was in the range of 20–40 mW/mm^2 at the fiber tip.

Post mortem assessment of electrode position

Wide field fluorescence images were acquired to reconstruct the recording electrode position in brain slices of electrophysiologically investigated pups and to localize tDimer2 expression in pups after IUE. Only pups with correct electrode and transfection position were considered for further analysis.

Histology

Perfusion

P8–10 mice were anesthetized with 10% ketamine (aniMedica, Germany) / 2% xylazine (WDT, Germany) in 0.9% NaCl solution (10 μg/g body weight, intraperitoneally (i.p.)) and transcardially perfused with Histofix (Carl Roth, Germany) containing 4% paraformaldehyde.

Immunohistochemistry

Brains were postfixed in Histofix for 24 h and sectioned coronally at 50 μm (immunohistochemistry) or 100 μm (Sholl and spine analysis). For anti-NeuN, anti-CamKII and anti-GABA stainings, free-floating slices were permeabilized and blocked with PBS containing 0.8% Triton X-100 (Sigma-Aldrich, MO, USA) and 5% normal bovine serum (Jackson Immuno Research, PA, USA). For IBA-1 and VGLUT1 stainings, slices were permeabilized and blocked with PBS containing 0.3% Triton X-100 and 3% normal bovine serum. Subsequently, slices were incubated overnight with mouse monoclonal Alexa Fluor-488-conjugated antibody against NeuN (1:100, MAB377X; Merck Millipore, MA, USA), rabbit polyclonal primary antibody against CaMKII (1:200, PA5-38239; Thermo Fisher Scientific, MA, USA), rabbit polyclonal primary antibody against GABA (1:1,000, no. A2052; Sigma-Aldrich), rabbit monoclonal primary antibody against IBA-1 (1:500, catalog #019-19741, Wako), or polyclonal guinea-pig antibody against VGLUT1 (1:1000, Synaptic Systems, Germany) followed by 2 h incubation with Alexa Fluor-488 goat anti-rabbit IgG secondary antibody (1:500, A11008; Merck Millipore), Alexa Fluor-568 donkey anti-rabbit (1:500, Life Technologies, CA, USA) or Alexa Fluor-488 goat anti-guinea pig (1:500, Molecular Probes, OR, USA). Finally, slices were transferred to glass slides and covered with Vecta-Shield (Vector Laboratories).

Imaging

Sections were examined with a confocal microscope (DM IRBE, Leica Microsystems, Zeiss LSN700 and Olympus FX-100). To quantify the t-Dimer overlap with NeuN, CaMKII and GABA, microscopic fields over PFC were acquired as 1024 × 1024 pixel images (pixel size, 1465 nm) using a 10X objective (numerical aperture, 0.3). The same settings were used to quantify the number of CaMKII positive neurons (n = 4 fields per section, 3 sections per mouse). For IBA-1, 20-images microscopic stacks (n = 8 stacks per section, 3 sections per mouse) were acquired as 512 × 512 pixel images (pixel size, 732 nm; Z-step, 1000 nm) using a 40X objective (numerical aperture, 1.25). For analysis of IBA-1$^+$-cells and VGLUT1 vesicle overlay, microscopic stacks (n = 5 stacks per sections, 3 sections per mouse) were acquired as 1024x1024 pixels images (pixel size, 103 nm; Z-step, 750 nm) using a 60X objective (numerical aperture, 1.35). Microscopic stacks used for Sholl and spine analysis were acquired as 2048 × 2048 pixel images (pixel size, 156 nm; Z-step, 1000 and 500 nm, respectively).

QUANTIFICATION AND STATISTICAL ANALYSIS

Image analysis

CaMKII$^+$ cells quantification

The number of CaMKII-positive neurons was semi-automatically assessed with a custom-written algorithm in the ImageJ environment. Briefly, a Region of Interest (ROI) was manually placed over either superficial or deep prefrontal layers. The image contrast was enhanced (*enhance contrast* function, 0.5% of saturated pixels) and a *median filter* was applied (radius = 1.5). To reduce background noise, we used the *subtract background* function, with a radius of 30 pixels. The image was then binarized (*convert to mask*) and segmented using the *watershed* function. To identify the neurons we used the *extended maxima* function of the MorphoLibJ plugin (dynamic = 30, connectivity = 4). We subtracted the regional maxima with the lowest intensity (i.e., the objects with bigger area) using *area opening* (pixel = 150) and counted the remaining objects (*analyze particles*).

Neuronal morphological analysis

Sholl analysis and spine density quantification were carried out in the ImageJ environment. For Sholl analysis, images were binarized (*auto threshold*) and dendrites were traced using the semi-automatical plugin *Simple Neurite Tracer*. The traced dendritic tree was analyzed with the plugin *Sholl Analysis*, after the geometric center was identified using the *blow/lasso tool*. For spine density quantification, we first traced the dendrite of interest (apical, basal, proximal oblique or secondary apical) and measured its length (*line*) and then manually counted the number of spines (*point picker*).

Iba-1$^+$-cells quantification

To quantify the number of Iba-1 stained cells we used a custom-written algorithm in ImageJ. The image stacks were collapsed to a maximum intensity Z-projection, and background noise was subtracted (*despeckle*). To facilitate automatic thresholding, the image was passed through a Gaussian filter (*Gaussian blur*, sigma = 2) before being binarized (*auto threshold* with the *triangle* method). The number of cells was counted using *analyze particles* (size >150 pixels).

Iba-1$^+$-cells morphological analysis

The morphology of microglial cells was assessed on maximum intensity Z-projections in the MATLAB environment, using previously reported criteria (Bellesi et al., 2017). Images (n = 64 for each group of mice) were automatically thresholded (*graythresh* and *im2bw* functions) and putative microglial cells were identified as objects between 200 and 1500 pixels (*bwareaopen*). Around the center of mass of each of the isolated cells, a region of interest (ROI) of 110x110 pixels was computed and visually examined. If the ROI contained a properly segmented microglia cells, its features (area, perimeter, eccentricity) were quantified (*regionprops*). ROIs in which the microglial cell touched the boundaries of the image or in which more than one cell was included were discarded. Further, cell spread (analogous to process length) was computed as the average distance between the center of mass and the "extrema" of the cell; roundness was defined as the ratio between 4*pi*area and the square of the perimeter of the cell. Only mice that did not experience IUE were used for this analysis.

Iba-1$^+$-cells and VGLUT1$^+$ puncta overlay

Overlay of Iba-1$^+$ cells and VGLUT1$^+$ puncta was assessed according to previously reported criteria (Bellesi et al., 2017). Briefly, background noise of VGLUT1 stacks was reduced in the ImageJ environment using the subtract background (rolling ball radius, 2 pixels) and despeckle functions. Stacks were passed through a maximum filter (radius, 2 pixels), thresholded (auto threshold) and segmented (watershed). Further processing was carried out in the MATLAB environment. Puncta were labeled (bwlabeln, connectivity = 8) and their volume was measured (histcounts). Puncta <100 pixel or >500 pixel were discarded. Microglia stacks were entirely processed in the MATLAB environment. Stacks were passed through a 3D hysteresis filter (hysteresis3d function; lower threshold = 0.1, upper threshold = 0.5, connectivity = 26) and a 3D median filter (ordfilt3D function). VGLUT1 positive puncta showing 100% overlap with the processed Iba1 signal were then quantified. VGLUT-1 puncta were considered to be phagocyted if they showed a 100% overlap in xyz with the imaged microglial cell. Microglia cells were also quantified in their distal cell volume (volume computed starting from 7 μm of distance from the center of mass of the cell).

In vitro electrophysiology

As previously described (Bitzenhofer et al., 2017), data were imported and analyzed offline using custom-written tools in the MATLAB environment (MathWorks). For *in vitro* data, all potentials were corrected for liquid junction potentials (−10 mV) for the gluconate-based electrode solution. The RMP was measured immediately after obtaining the whole-cell configuration. To assess input resistance, hyperpolarizing current pulses of 200 ms duration were applied. Active membrane properties and current–voltage relationships were determined by unsupervised analysis of responses to a series of 600 ms long hyper- and depolarizing current pulses. Amplitude of APs was measured from threshold to peak.

In vivo electrophysiology

In vivo data were analyzed with custom-written algorithms in the MATLAB environment. Data were processed as following: band-pass filtered (500–5,000 Hz) to analyze MUA and band-pass filtered (4-100 Hz) using a third-order Butterworth filter before downsampling to 3.2 kHz to analyze LFP. All filtering procedures were performed in a phase preserving manner. In recordings of non-anesthetized mice, to reduce the influence of movement-related artifacts, the signal was processed according to the *common-average and rereference* method before power spectral analysis and spike detection.

Detection of oscillatory activity

The detection of discontinuous patterns of activity in the neonatal PL was performed using a modified version of the previously developed algorithm for unsupervised analysis of neonatal oscillations (Cichon et al., 2014). Briefly, deflections of the root mean square of band-pass filtered signals (1–100 Hz) exceeding a variance-depending threshold were considered as network oscillations. The threshold was determined by a Gaussian fit to the values ranging from 0 to the global maximum of the root-mean-square histogram. If two oscillations occurred within 200 ms of each other they were considered as one. Only oscillations lasting >1 s were included, and their occurrence, duration, and amplitude were computed.

Power spectral density

For power spectral density analysis, 1 s-long windows of network oscillations were concatenated and the power was calculated using Welch's method with non-overlapping windows. For optical stimulation, we compared the average power during the 1.5 s-long time window preceding the stimulation to the last 1.5 s-long time window of light-evoked activity.

Multi-unit activity

MUA was detected as the peak of negative deflections exceeding five times the standard deviation of the filtered signal and having a prominence larger than half the peak itself.

Single unit activity

SUA was detected and clustered using klusta (Rossant et al., 2016) and manually curated using phy (https://github.com/cortex-lab/phy). Data were imported and analyzed using custom-written tools in the MATLAB software (MathWorks).

Firing rate

The firing rate was computed by dividing the total number of spikes by the duration of the analyzed time window.

Inter-spike-interval

Inter-spike interval (ISI) was calculated at 2 ms resolution and was normalized to all the detected ISI. For plotting and statistics only the 10-500 ms range was considered.

Pairwise phase consistency

Pairwise phase consistency (PPC) was computed as previously described (Vinck et al., 2010). Briefly, the phase in the band of interest was extracted as mentioned above, and the mean of the cosine of the absolute angular distance (dot product) among all pairs of phases was calculated.

Spike-triggered LFP power

Spike-triggered LFP spectra were calculated as

$$\left(\mathrm{Power_{spike}} - \mathrm{Power_{baseline}}\right) \big/ \mathrm{Power_{baseline}},$$

where the spike-triggered power spectrum ($\mathrm{Power_{spike}}$) was calculated using Welch's method for a 200 ms-log time window centered on each spike, and the power spectrum of baseline LFP ($\mathrm{Power_{baseline}}$) was averaged for two time windows, 100-300 ms and 200-400 ms before each spike.

K-nearest neighbors classifiers

Machine learning analyses were performed using Python (Python Software Foundation, Wolfeboro Falls, New Hampshire, USA) in the Spyder (Pierre Raybaut, The Spyder Development Team) development environment. Model training and performance evaluation were carried out using the scikit-learn toolbox. The set was iteratively (n = 500) divided in a training (2/3 of the set) and a cross-validation (1/3) set. Hyper-parameter of the model were tuned on the training set, which was further split using the standard 3-fold cross-validation split implemented by the function "GridSearchCV," to which a "pipeline" object was passed. The "pipeline" object was built using the "Pipeline" function, and concatenating quantile transformation of the input features ("Quantile Transformer," tuning the number of quantiles), feature selection ("Select Percentile," using mutual information and tuning the percentage of features to select) and K-nearest neighbors classification (tuning the number of neighbors, the weight function to use for prediction, the algorithm used to compute the nearest neighbors, and the size of the leaf). Performance assessment was then computed on the cross-validation set (to which it had not been exposed during hyper-parameters tuning). Performance was stable across a wide range of parameters. To plot the classifier decision space, we used t-sne to reduce the feature space to two dimensions, while preserving the hyper-dimensional structure of the data. The decision space was then approximated by imposing a Voronoi tessellation on the 2d plot, using k-nearest regression on the t-sne coordinates of the predicted classes of the mice.

Statistical analysis

Statistical analyses were performed using R Statistical Software (Foundation for Statistical Computing, Vienna, Austria). Normally distributed, homoscedastic, having equal variance and non-nested data were tested for significant differences (*$p < 0.05$, **$p < 0.01$ and ***$p < 0.001$) using paired t test, unpaired t test, one-way repeated-measures ANOVA, or one-way ANCOVA with age as a covariate (only if age had a significant effect) and with Bonferroni-Tukey corrected post hoc analysis. Not normally distributed, heteroskedastic or not having equal variance data were tested with yuen's bootstrap test (n = 5000 repetitions), yuen's paired sample robust t test, or bootstrapped (n = 5000 repetitions) heteroscedastic one way ANOVA for trimmed means (yuenbt, yuend, t1waybt, mcppb20, glht, lsmeans functions of the WRS2, multcomp and lsmeans R package). A standard 20% level of trimming for the mean

was selected for these tests. Such tests were preferred to more traditional non-parametric tests in virtue of the (sometimes) high levels of unequal variance in our data. To account for the commonly ignored increased false positive rate inherent in nested design (Aarts et al., 2014), nested data were analyzed with linear mixed-effect models. Parameter estimation was done using the *lmer* function implemented in the lme4-R package. Model selection was performed using the Akaike Information Criterion (AIC) and/or the Bayesian information criterion (BIC), as differences between the two criteria were minimal. To test the significance of condition in our model, we performed a likelihood ratio test against a reduced model in which we removed condition (aov R function). Post hoc analysis with Tukey multiple comparison correction was carried out using the glhp function of the multcomp R package. For analysis of phagocytic activity of microglia cells, we used bootstrapped heteroscedastic one-way ANOVA for trimmed means instead of linear mixed-effect model in virtue of the highly non-normality of the data. No statistical measures were used to estimate sample size since effect size was unknown. Investigators were blinded to the group allocation when Sholl, spine analyses, microglia morphology, and engulfment were assessed. For other investigations, unsupervised analysis software was used to preclude investigator biases. Statistical parameters can be found in the main text and/or in the figure legends. More information about test used, its values, and its parameters are provided in Data S1.

DATA AND CODE AVAILABILITY

The authors declare that all data and code supporting the findings of this study are included in the manuscript and its Supplementary Information or are available from the corresponding authors on request. LFP and SUA data for all the non-anesthetized mice is available at the following open-access repository:

https://gin.g-node.org/mchini/Resolving_and_rescuing_developmental_miswiring_in_a_mouse_model_of_cognitive_impairment

Neuron, Volume *105*

Supplemental Information

Resolving and Rescuing Developmental Miswiring in a Mouse Model of Cognitive Impairment

Mattia Chini, Jastyn A. Pöpplau, Christoph Lindemann, Laura Carol-Perdiguer, Marilena Hnida, Victoria Oberländer, Xiaxia Xu, Joachim Ahlbeck, Sebastian H. Bitzenhofer, Christoph Mulert, and Ileana L. Hanganu-Opatz

Resolving and rescuing developmental miswiring in a mouse model of cognitive impairment

Mattia Chini[1], Jastyn A. Pöpplau[1], Christoph Lindemann[1], Laura Carol-Perdiguer[1], Marilena Hnida[1], Victoria Oberländer[1,†], Xiaxia Xu[1], Joachim Ahlbeck[1,††], Sebastian H. Bitzenhofer[1,†††], Christoph Mulert[2,§] & Ileana L. Hanganu-Opatz[1,3*]

Inventory of Supplemental Information	page
Figure S1, related to Fig. 1	2
Figure S2, related to Fig. 1	4
Figure S3, related to Fig. 2-5	6
Figure S4, related to Fig. 2-3	8
Figure S5, related to Fig. 2	10
Figure S6, related to Fig. 3	11
Figure S7, related to Fig. 4	12
Figure S8, related to Fig. 5-6	14
Figure S9, related to Fig. 8	16
Data Files S1	17
Table S1	18

SUPPLEMENTARY INFORMATION

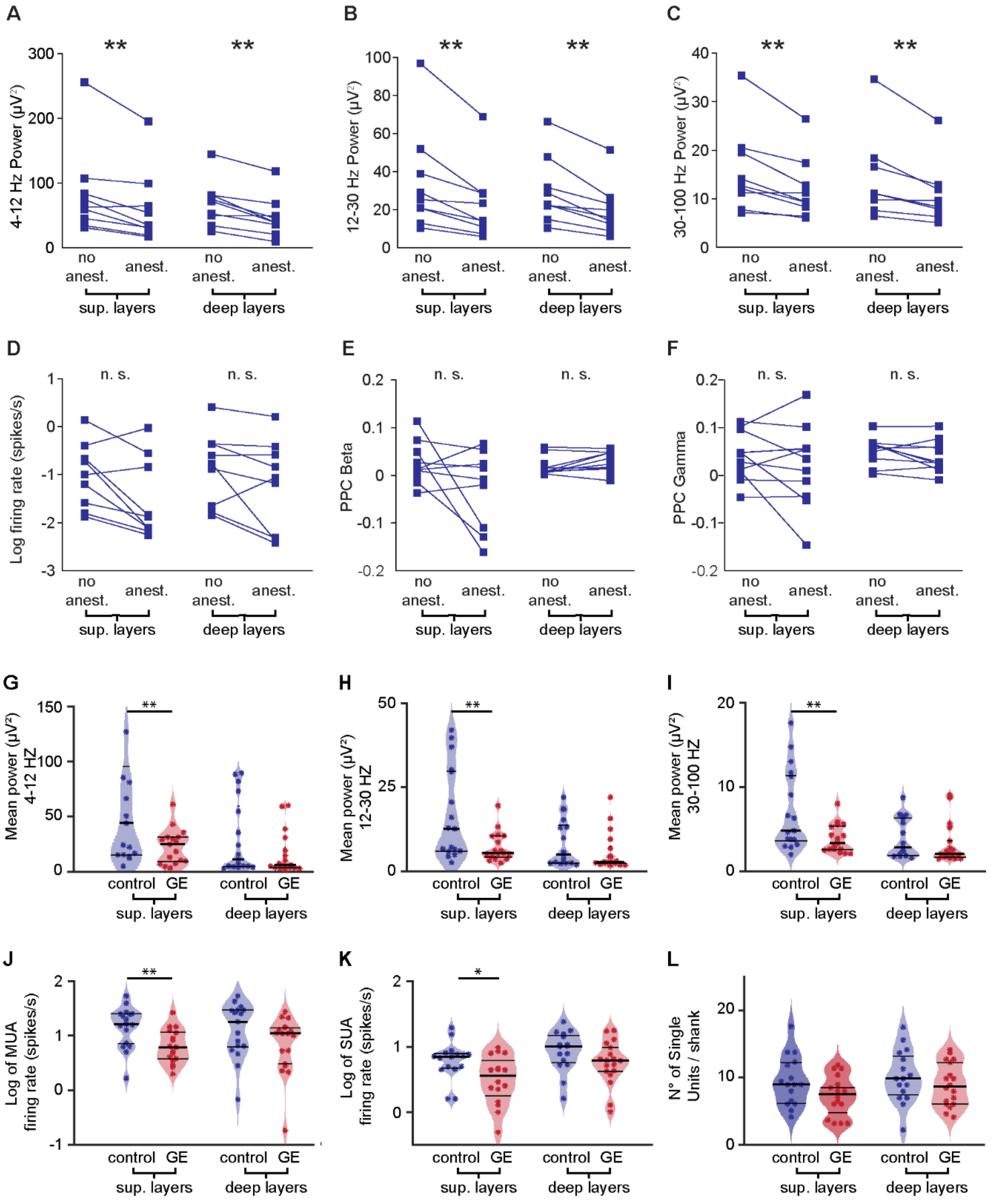

Figure S1. Related to Figure 1. Effect of urethane anesthesia on network oscillations and firing of neonatal control and GE mice. (A) Scatter plot displaying the oscillatory power in theta frequency band for superficial and deep layers of the prelimbic cortex before (left, n=9 mice) and after urethane anesthesia (right, n=9 mice). **(B-F)** Same as (A) for power in beta (B) and gamma (C) frequency bands, MUA firing rate (D), as well as pairwise phase consistency for beta (E) and gamma (F) oscillations. **(G)** Violin plot displaying the power for theta frequency band in superficial and deep layers of the prelimbic cortex of non-anesthetized control (blue, n=16) and GE (red, n=18) mice. **(H-L)** Same as (G) for power in beta (H) and gamma (I)

frequency bands, MUA (J) and SUA (K) firing rate, and number of single units per shank (L). In scatter plots (A-F), single data points are presented as squares. In violin plots (G-L), data is presented as median with 25th and 75th percentile and single data points are shown as asterisks. *P<0.05, **P<0.01 and ***P<0.001, Wilcoxon signed-rank test (A-F), ANCOVA with age as covariate (G-L), and yuen's bootstrap test (J-L) with 20% level of trimming for the mean.

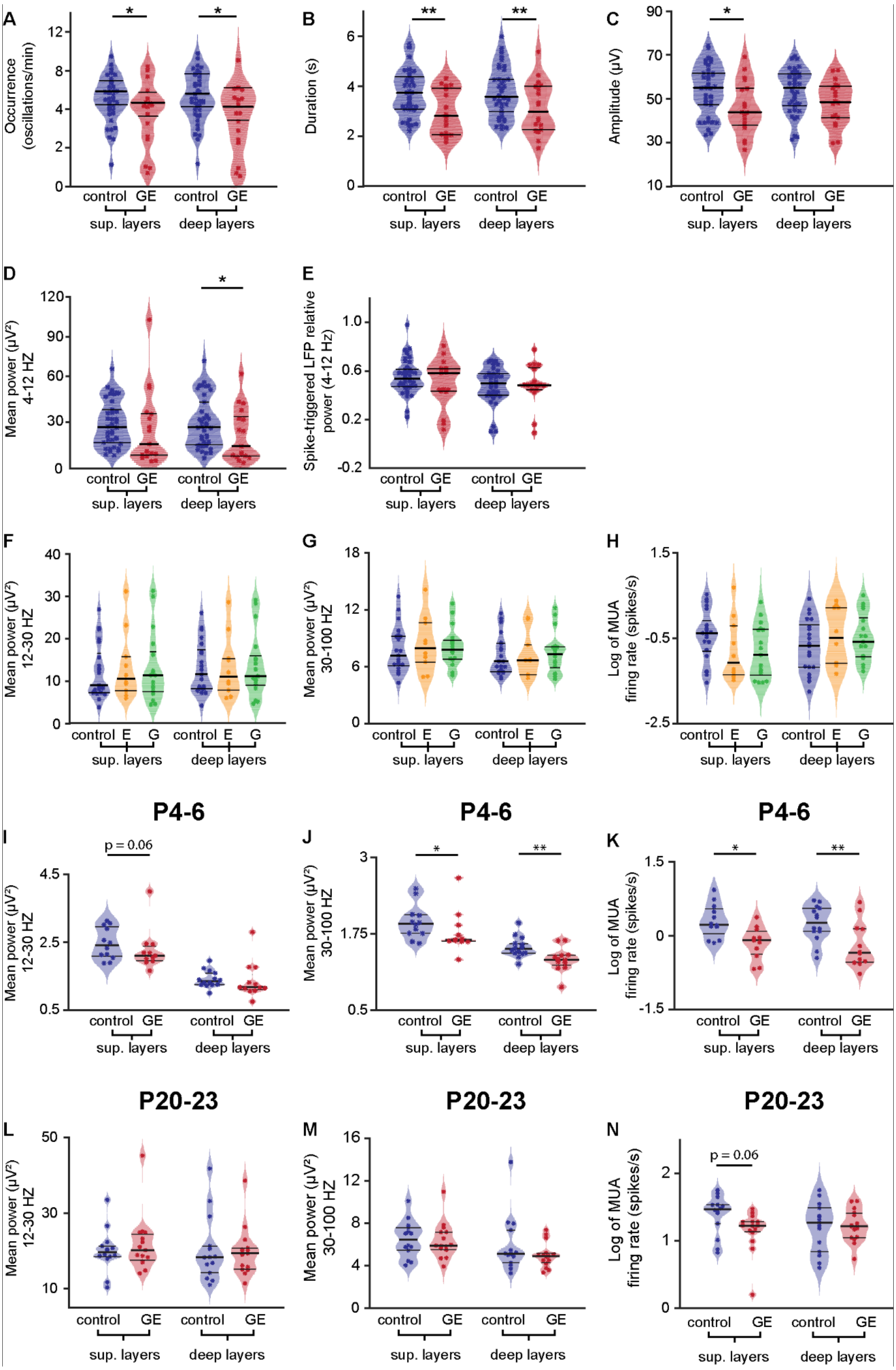
A
Occurrence (oscillations/min)
*
*
control GE control GE
sup. layers deep layers
B
Duration (s)
**
**
control GE control GE
sup. layers deep layers
C
Amplitude (µV)
*
control GE control GE
sup. layers deep layers
D
Mean power (µV²) 4-12 HZ
*
control GE control GE
sup. layers deep layers
E
Spike-triggered LFP relative power (4-12 Hz)
control GE control GE
sup. layers deep layers
F
Mean power (µV²) 12-30 HZ
control E G control E G
sup. layers deep layers
G
Mean power (µV²) 30-100 HZ
control E G control E G
sup. layers deep layers
H
Log of MUA firing rate (spikes/s)
control E G control E G
sup. layers deep layers
P4-6
I
Mean power (µV²) 12-30 HZ
p = 0.06
control GE control GE
sup. layers deep layers
P4-6
J
Mean power (µV²) 30-100 HZ
*
**
control GE control GE
sup. layers deep layers
P4-6
K
Log of MUA firing rate (spikes/s)
*
**
control GE control GE
sup. layers deep layers
P20-23
L
Mean power (µV²) 12-30 HZ
control GE control GE
sup. layers deep layers
P20-23
M
Mean power (µV²) 30-100 HZ
control GE control GE
sup. layers deep layers
P20-23
N
Log of MUA firing rate (spikes/s)
p = 0.06
control GE control GE
sup. layers deep layers

Figure S2. Related to Figure 1. Properties of discontinuous oscillatory activity and neuronal firing over the layers of PL from neonatal one-hit and dual-hit GE mice as well as P4-6 and P20-23 control and dual-hit GE mice. (A) Violin plot displaying the occurrence of oscillations in prelimbic superficial and deep layers of control (blue, n=38) and GE (red, n=18) mice. **(B-E)** Same as (A) for oscillation duration (B), amplitude (C), power in theta frequency band (D), the relative power of spike-triggered LFP in theta band (E). **(F)** Violin plot displaying the mean power (12-30 Hz) of oscillations in prelimbic superficial and deep layers of control (blue, n=21), E (yellow, n=10) and G (green, n=17) neonatal mice. (**G,H**) Same as (F) for mean power (30-100 Hz) (G) and MUA firing rate (H). (**I**) Violin plot displaying the mean power (12-30 Hz) in prelimbic superficial and deep layers of control (blue, n=14) and GE (red, n=13) P4-6 mice. (**J,K**) Same as (I) for mean power (30-100 Hz) (J) and MUA firing rate (K). (**L**) Violin plot displaying the mean power (12-30 Hz) in prelimbic superficial and deep layers of control (blue, n=14) and GE (red, n=15) P20-23 mice. (**M,N**) Same as (L) for mean power (30-100 Hz) (M) and MUA firing rate (N). Data is presented as median with 25th and 75th percentile and single data points are shown as asterisks. The shaded area represents the probability distribution of the variable. *P<0.05, **P<0.01, ANCOVA with age as covariate (A-D, F-K), and yuen's bootstrap test (E, I-N) with 20% level of trimming for the mean.

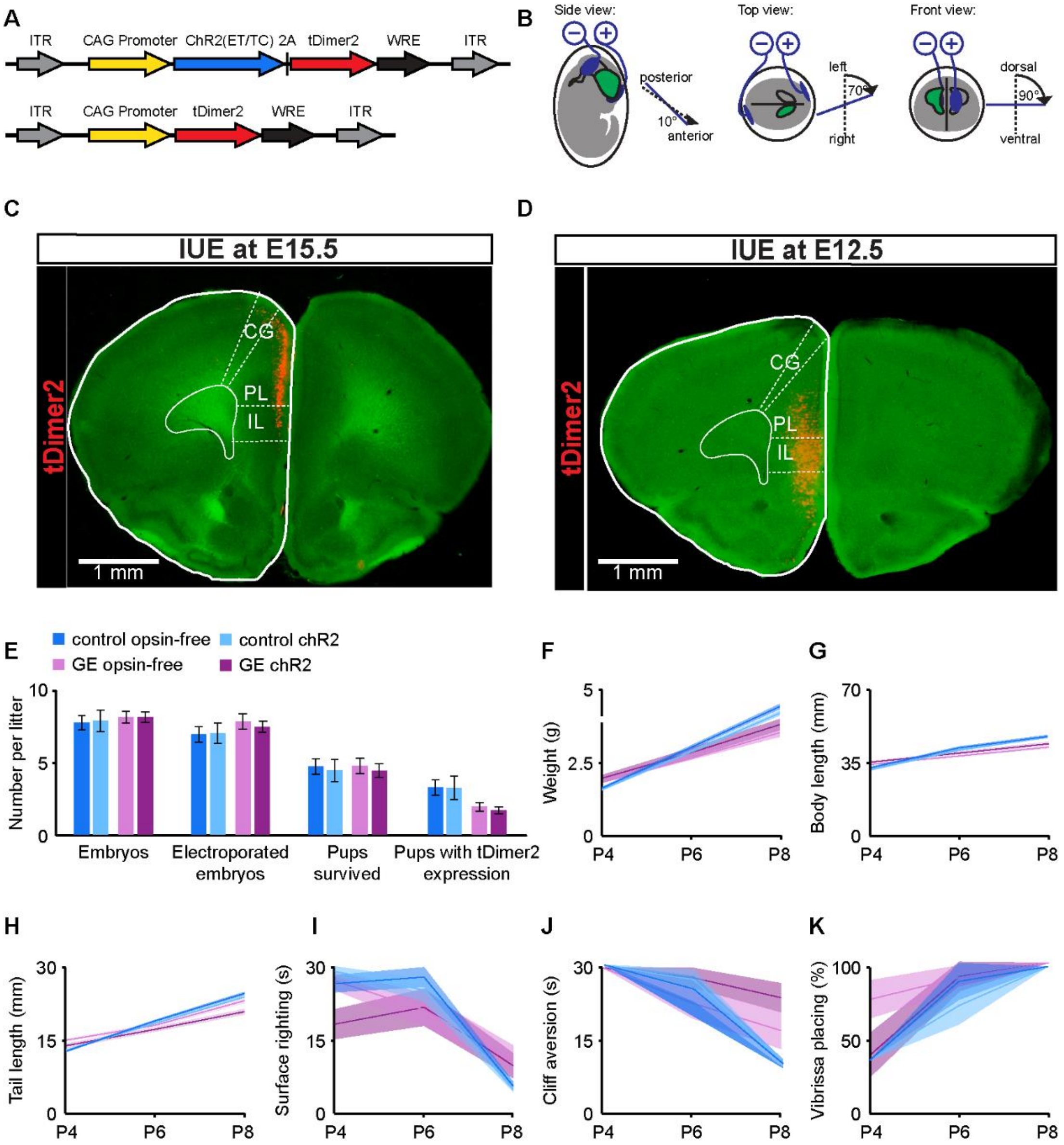

Figure S3. Related to Figures 2-5. Cell- and layer-specific transfection of neonatal prelimbic cortex by site-directed in utero electroporation. (a) Structure of the ChR2(ET/TC)-containing and opsin-free plasmids. **(B)** Schematic drawing illustrating the orientation of electrode paddles for specific targeting of $PYRs_{SUP}$ and $PYRs_{DEEP}$. **(C)** TDimer2-expressing cells (red) in a 50 µm-thick coronal section of a P10 mouse at the level of PFC after IUE at E15.5. Note that the transfection is restricted to neurons located in superficial layers. **(D)** Same as (C) after IUE at E12.5. Note that the transfection is mainly restricted to neurons located in deep layers. **(E)** Bar diagram displaying the mean number of embryos, electroporated embryos, surviving pups, and positively transfected pups when ChR2(ET/TC)-containing (control, dark blue, n=13 litters; GE, dark purple, n=10 litters) and opsin-free constructs (control, light blue, n=5 litters; GE, light purple, n=8 litters) were transfected by IUE at E15.5. **(F-H)** Line plots displaying the developmental profile of somatic growth: weight (F), body length (G), tail length (H) of P2-8 pups expressing ChR2(ET/TC) (control, dark blue, n=31; GE, dark purple, n=26) or opsin-free constructs (control, light blue, n=11; GE, light purple, n=7) in $PYRs_{SUP}$. **(I-K)** Line plots displaying the development profile of reflexes: surface righting (I), cliff aversion (J), and vibrissa placing (K) of P2-8 pups expressing ChR2(ET/TC)

(control, dark blue, n=31; GE, dark purple, n=26) or opsin-free constructs (control, light blue, n=11; GE, light purple, n=7) in $PYRs_{SUP}$. Data are displayed as mean ± SEM. *$p < 0.05$, one-way repeated-measures analysis of variance (ANOVA) with Bonferroni-corrected post hoc analysis.

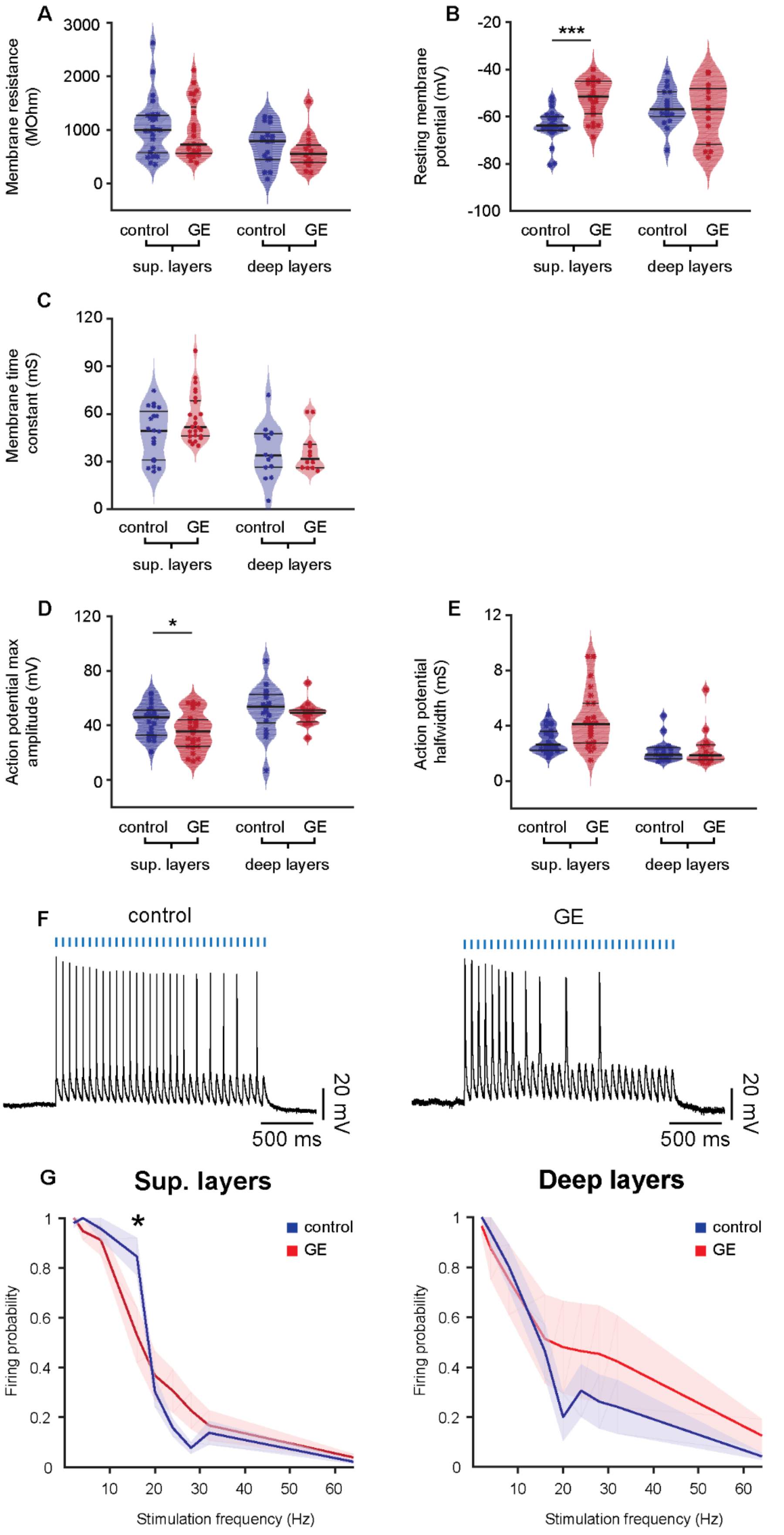

Figure S4. Related to Figures 2-3. Optogenetic activation of $PYRs_{SUP}$ and $PYRs_{DEEP}$ in control and dual-hit GE mice in vitro. (A) Violin plot displaying the membrane resistance for

$PYRs_{SUP}$ and $PYRs_{DEEP}$ of control (blue, n=25 and n=24, respectively) and GE mice (red, n=17 and n=14, respectively). **(B-E)** Same as (A) for resting membrane potential (B), membrane time constant (C), maximum light-triggered action potential amplitude (D), and light-triggered action potential half-width (E). **(F)** Representative voltage responses of a transfected $PYRs_{SUP}$ from a P9 control (left) and a P9 GE mouse (right) to repetitive trains of 3 ms-long light pulses at 16 Hz. **(G)** Graph displaying the mean firing probability of superficial (left) and deep layers (right) transfected control and GE neurons in response to repetitive light stimuli of different frequencies. In (G) data is presented as mean ± sem. In (A-E) data is presented as median with 25th and 75th percentile and single data points are shown as asterisks. The shaded area represents the probability distribution of the variable. *P<0.05, **P<0.01 and ***P<0.001, one-way repeated-measures analysis of variance (ANOVA) with Bonferroni-corrected post hoc analysis (F), and bootstrap yuen test with 20% level of trimming for the mean and 5000 bootstrap samples.

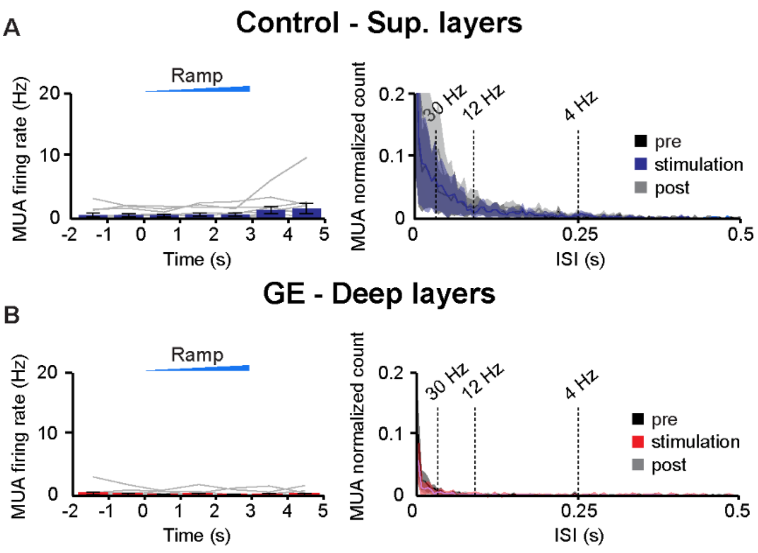

Figure S5. Related to Figure 2. In vivo spiking of PYRs$_{SUP}$ transfected by IUE with opsin-free constructs. (A) Left, bar diagram displaying the mean MUA firing rate in transfected PYRs$_{SUP}$ of control mice in response to ramp illumination. Right, occurrence rate of interspike intervals averaged for 3 s before light stimulation (pre, black), 3 s during ramp stimulation (stimulation, blue) and 3 s after light stimulation (post, grey, n=11 recording sites from 11 pups). **(B)** Same as (A) for PYRs$_{SUP}$ of GE mice (n=10 recording sites from 8 pups). Data is presented as mean ± s.e.m. P>0.05, one-way repeated-measures analysis of variance (ANOVA) with Bonferroni-corrected post hoc analysis.

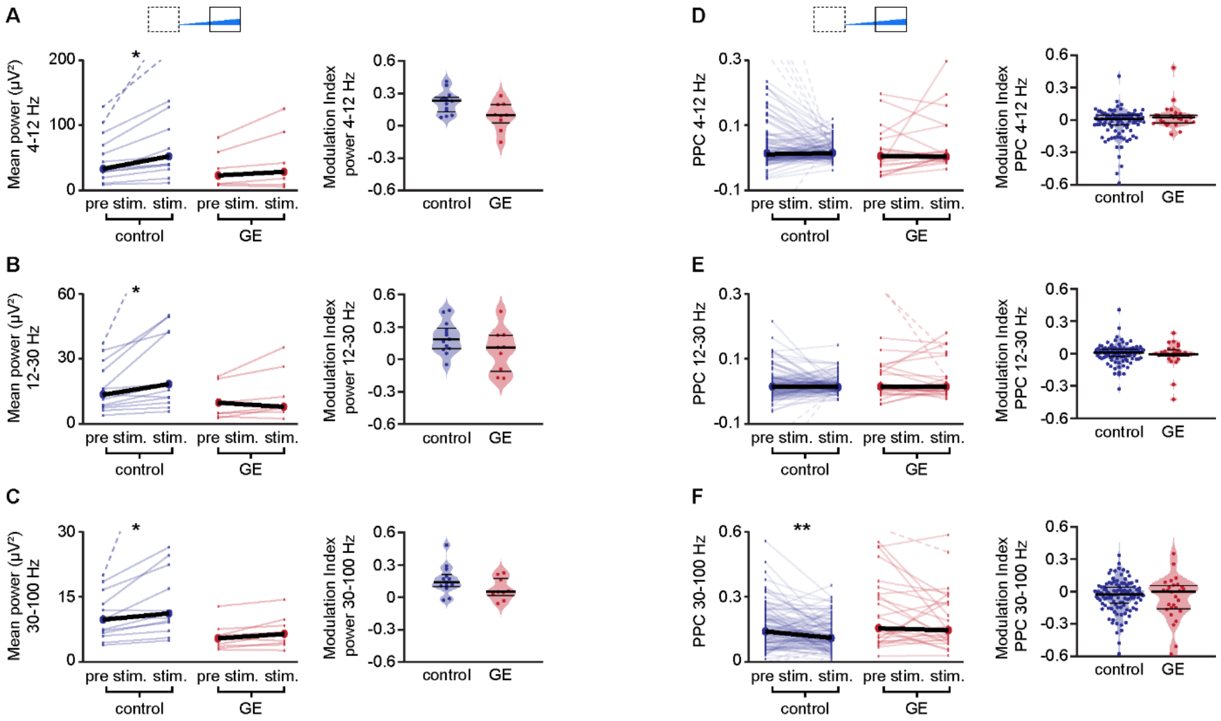

Figure S6. Related to Figure 3. Network activity after optogenetic activation of PYRs$_{DEEP}$ in control and dual-hit GE mice in vivo. (A) Left, scatter plot displaying the LFP power in the theta (4–12 Hz) frequency band for control (blue, n=13) and GE (red, n=9) mice before (pre stim., 1.5 s) and during the second half (stim., 1.5 s) of ramp stimulation. Right, violin plot displaying the stimulation modulation index of LFP power in the theta frequency band for control and GE mice. **(B,C)** Same as (A) for beta (12-30 Hz) and gamma (30-100) frequency bands. **(D-F)** Same as (A-C) for PPC of PYRs$_{DEEP}$ in control (n=116 recording sites from 13 pups) and GE (n=27 recording sites from 6 pups) mice. In scatter plots (A-F) data is presented as median, and individual values are displayed as thin dots and lines. In violin plots (A-F) data is presented as median with 25th and 75th percentile and single data points are shown as asterisks. The shaded area represents the probability distribution of the variable. *P<0.05, **P<0.01 and ***P<0.001, yuen's bootstrap test (A-F) with 20% level of trimming for the mean and linear mixed-effect model with animal as a random effect (D-F).

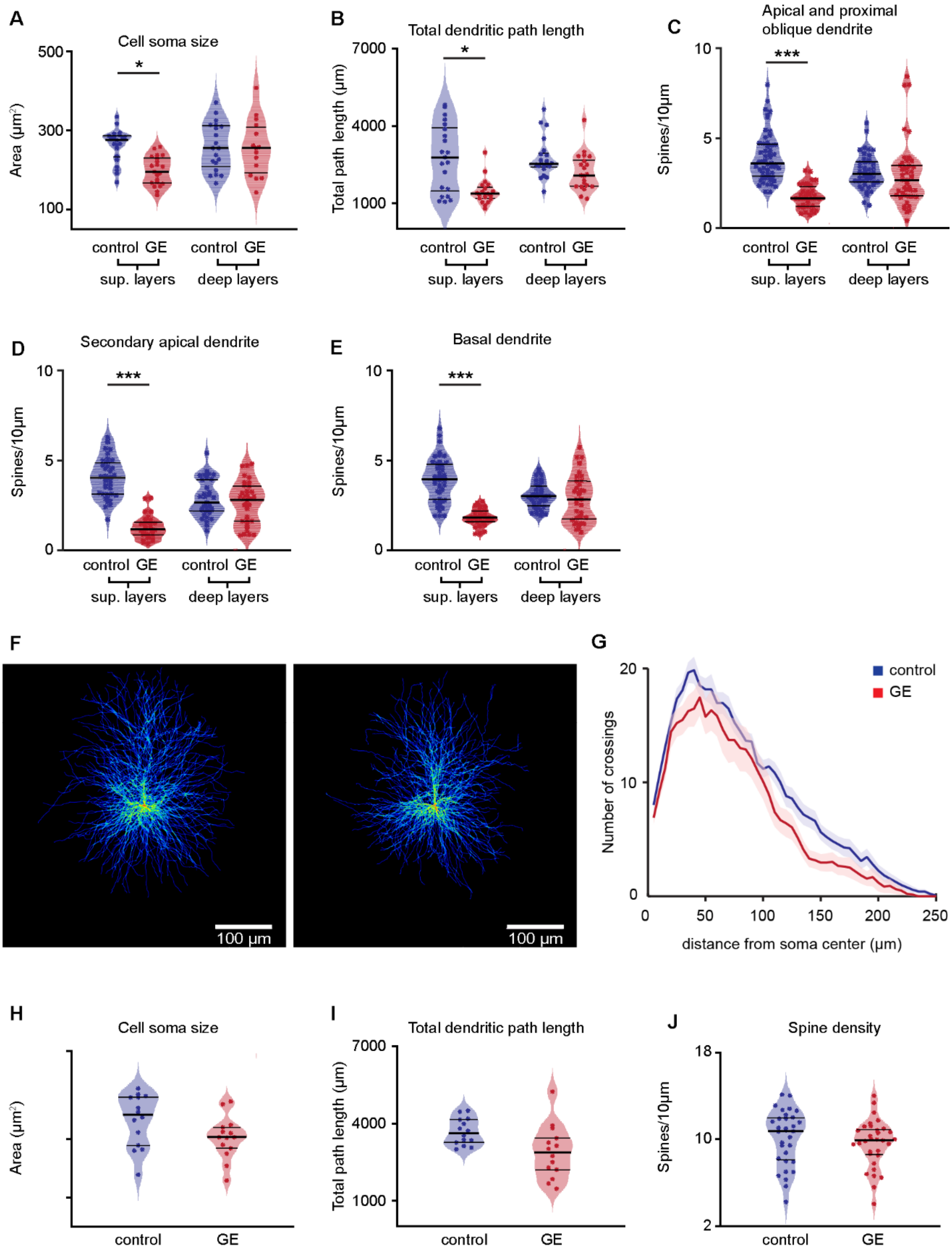

Figure S7. Related to Figure 4. Reduced soma size, dendritic path length and spine density in PYRs$_{SUP}$ of neonatal but not pre-juvenile dual-hit GE mice. (A) Left, violin plot displaying the average soma size from PYRs$_{SUP}$ of control (blue, n=21 neurons) and GE (red, n=21 neurons) mice. Right, same as left for PYRs$_{DEEP}$ from control (blue, n=21 neurons) and GE (red, n=15 neurons) mice. **(B)** Same as (A) for total dendritic path length. **(C)** Same as (A) for spine density on apical and proximal oblique dendrites from PYRs$_{SUP}$ of control (blue, n=53 dendrites from 13 neurons) and GE (red, n=35 dendrites from 10 neurons) mice. Right, same as left for PYRs$_{DEEP}$ from control (blue, n=43 dendrites from 12 neurons) and GE (red, n=44

dendrites from 12 neurons) mice. **(D,E)** same as (C) for secondary apical (B) and basal dendrites (C). **(F)** Heatmaps displaying an overlay of all traced dendrites of transfected $PYRs_{SUP}$ in pre-juvenile control (left) and GE (right) mice. **(G)** Graph displaying the average number of dendritic intersections within a 250 µm radius from the soma center of $PYRs_{SUP}$ in pre-juvenile control (blue, n=14 neurons from 2 pups) and GE (red, n=14 neurons from 2 pups) mice. **(H)** Violin plot displaying the average soma size from $PYRs_{SUP}$ of pre-juvenile control (blue, n=14 neurons) and GE (red, n=21 neurons) mice. (**I**) Same as (H) for total dendritic path length. (**J**) Violin plot displaying the average spine density on dendrites from $PYRs_{SUP}$ of pre-juvenile control (blue, n=24 dendrites from 8 neurons) and GE (red, n=24 dendrites from 8 neurons) mice. In (G) data is presented as mean ± s.e.m. In (A-E, H-J) data is presented as median with 25th and 75th percentile and single data points are shown as asterisks. The shaded area represents the probability distribution of the variable. ***P<0.001, linear mixed-effect model with animal as a random effect.

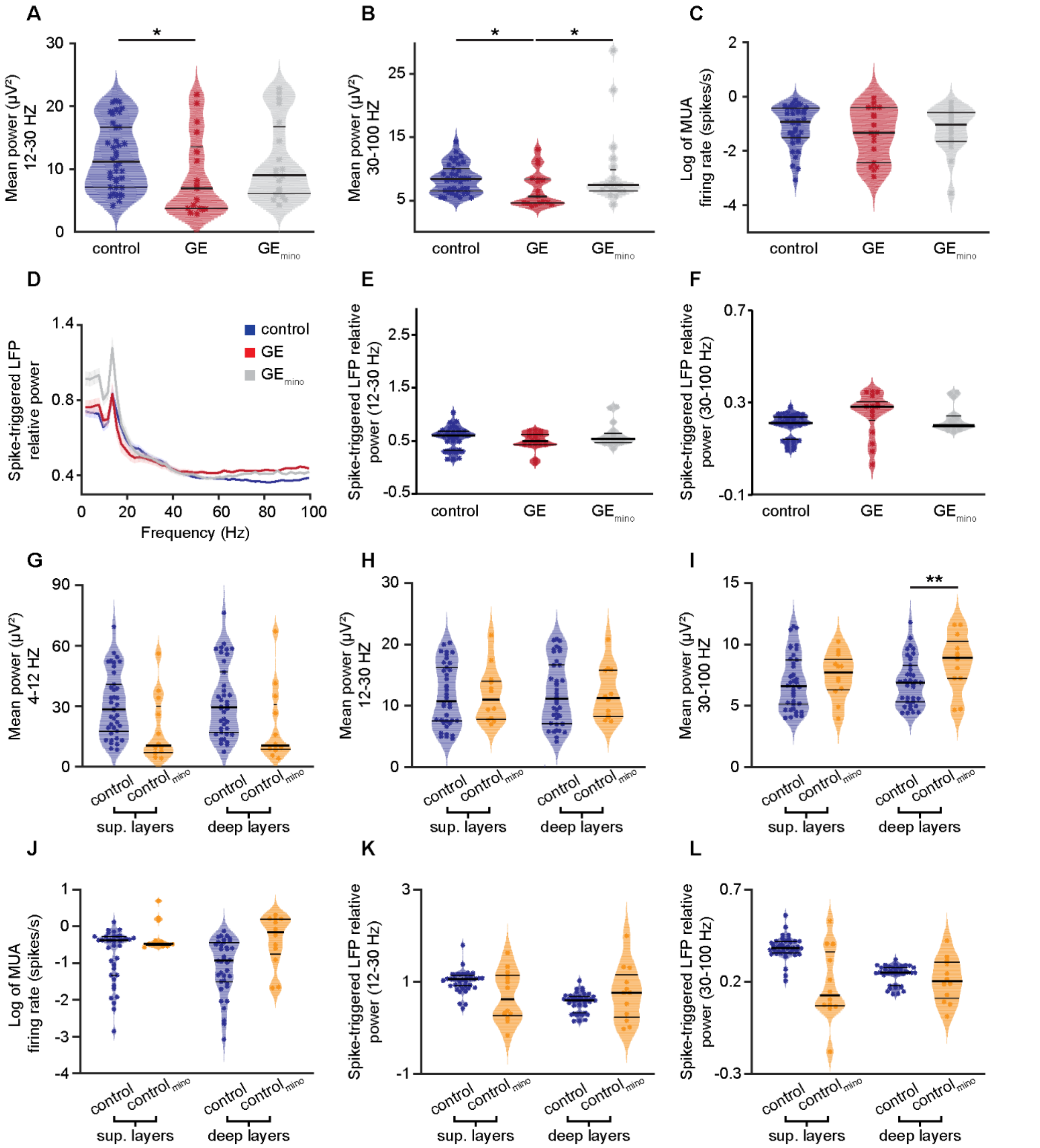

Figure S8. Related to Figure 5-6. Minocycline effect on neonatal control and dual-hit GE mice. (A) Violin plot displaying the power in beta frequency band of oscillations in deep layers of the PL of control (blue, n=38), GE (red, n=18), and GE_{mino} mice (gray, n=18). **(B-C)** Same as (A) for power in gamma (B) frequency bands and MUA firing rate (C). **(D)** Plots of frequency-dependent relative power of spike-triggered LFP in deep layers (top) of control (blue), GE (red), and GE_{mino} mice (gray). **(E)** Violin plot displaying the relative power of spike-triggered LFP in beta band for deep layers of control (blue, n=38), GE (red, n=18), and GE_{mino} mice (gray, n=18). **(F)** Same as (E) for the LFP in gamma band. **(G)** Violin plot displaying the power in theta frequency band of oscillations in superficial and deep layers of the prelimbic cortex of control (blue, n=38) and $control_{mino}$ (yellow, n=12) mice. **(H-L)** Same as (G) for the power in beta (H) and gamma (I) frequency band, MUA firing rate (J), relative power of spike-triggered LFP in beta (K) and gamma (L) band. In (D) data is presented as mean ± sem. In (A-C,E-L) data is presented as median with 25th and 75th percentile and single data points are shown as

asterisks. The shaded area represents the probability distribution of the variable. *P<0.05, **P<0.01, ANCOVA with age as covariate and 20% level of trimming for the mean (A-C), robust ANOVA with 20% level of trimming for the mean (E-F) with Bonferroni-Holm post-hoc analysis, and yuen's bootstrap test (G-L) with 20% level of trimming for the mean.

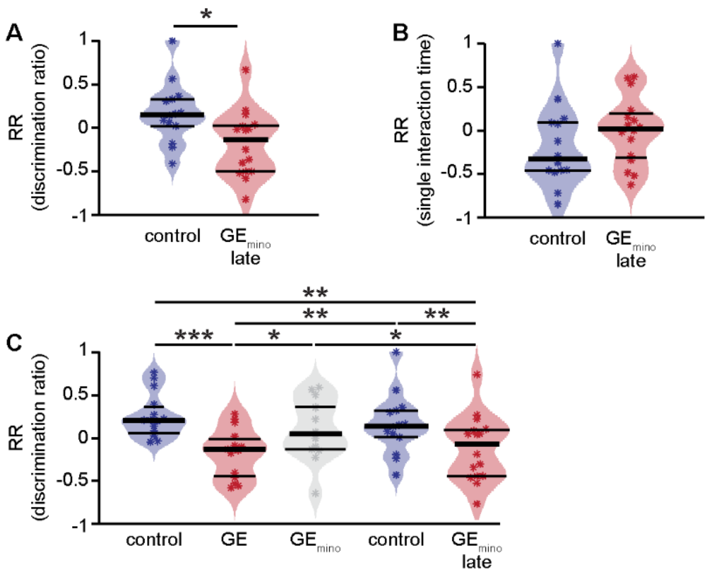

Figure S9. Related to Figure 8. Late minocycline treatment does not rescue behavioral deficits in dual-hit GE mice. (A) Violin plot displaying RR discrimination ratio of control (blue, n=14) and GE_{mino}late (red, n=15). **(B)** Same as (A) for single interaction time. **(C)** Same as (A) for control (blue, n=16), GE (red, n=15), GE_{mino} (gray, n=16), control (blue, n=14) and GE_{mino}late (red, n=15). Data is presented as median with 25th and 75th percentile and single data points are shown as asterisks. The shaded area represents the probability distribution of the variable. *P<0.05, robust, bootstrapped ANOVA with 20% level of trimming for the mean.

Data files S1. Related to Figures 1-8. Detailed statistical results.

	Superficial layers			*Deep layers*		
	**Control**	**GE**	**GE+mino**	**Control**	**GE**	**GE+mino**
Occurrence (oscillations/min)	6.6 ± 0.02	5.4 ± 0.2	6.7 ± 0.1	6.5 ± 0.05	5.2 ± 0.3 *	7 ± 0.1
Duration (s)	3.6 ± 0.1	3 ± 0.3 *	3.2 ± 0.03	3.7 ± 0.01	3 ± 0.26 *	3.2 ± 0.02
Amplitude (μV)	54.5 ± 1.1	45.7 ± 2.3 *	51.2 ± 0.4	54.3 ± 0.9	48.3 ± 1.6 *	49.8 ± 0.6
Theta power (μV^2)	28.9 ± 2.2	19.5 ± 4.4 **	23.6 ± 3.8	29.7 ± 2.5	19.3 ± 3.6 **	25.0 ± 4.8
Beta power (μV^2)	13.7 ± 0.7	7.0 ± 1.3 */°	10.2 ± 1.3	15.2 ± 0.7	6.9 ± 1.2 **	10.1 ± 1.4
Gamma power (μV^2)	6.6 ± 0.3	4.8 ± 0.4 **/°°	6.6 ± 0.7	6.7 ± 0.3	5.2 ± 0.5 **/°°	6.2 ± 0.5
Log firing rate (spikes/s)	-0.61 ± 0.04	-2-1 ± 0.1 ***/°°°	-0.9 ± 0.1	-0.95 ± 0.05	-1.3 ± 0.2	-0.96 ± 0.2
Spike triggered LFP relative power (4-12 Hz)	0.81 ± 0.02	0.89 ± 0.05 °°	1.3 ± 0.07 ^^	0.73 ± 0.03	0.67 ± 0.05 °	0.89 ± 0.05 ^
Spike triggered LFP relative power (12-30 Hz)	1.1 ± 0.03	0.64 ± 0.06 ***/°°	1.4 ± 0.17 ^	0.60 ± 0.04	0.50 ± 0.03	0.53 ± 0.03
Spike triggered LFP relative power (30-100 Hz)	0.38 ± 0.07	0.26 ± 0.03 ^/ */°	0.39 ± 0.02	0.25 ± 0.08	0.32 ± 0.02	0.24 ± 0.08

Table S1. Related to Figures 1, 6. Properties of oscillations in superficial and deep layers of PL in control, GE and GE_{mino} mice, as well as of their MUA firing and its timing to network oscillations. Asterisks (*) indicate significance level between control and GE mice. Circles (°) indicate significance level between GE and GE_{mino} mice. Carets (^) indicate significance level between control and GE_{mino} mice. ^/ */° $p<0.05$, ^^/ **/°° $p<0.005$, ^^^/ ***/°°° $p<0.001$.
